# Supplementary material for: Precursor-Directed Biosynthesis Mediated Amplification of Minor Aza Phenylpropanoid Piperazines in an Australian Marine Fish-Gut-Derived Fungus, Chrysosporium sp. CMB-F214
Source: Mar Drugs. 2021 Aug 25;19(9):478. doi: 10.3390/md19090478 (PMC8472109; doi:10.3390/md19090478)
Supplement: Supplementary file 1 [file marinedrugs-19-00478-s001.zip › marinedrugs-1354283-supplementary.pdf]

Supplementary Material

Precursor-directed biosynthesis mediated amplification of minor aza phenylpropanoid piperazines in an Australian marine fish-gut derived fungus, *Chrysosporium* sp. CMB-F214

Ahmed H. Elbanna,[1,2‡] Amila Agampodi Dewa,[1,‡] Zeinab G. Khalil[1] and Robert J. Capon[1,*]

[1] Institute for Molecular Bioscience, The University of Queensland, St Lucia, QLD, Australia

[2] Current Address: Department of Pharmacognosy, Faculty of Pharmacy, Cairo University, Cairo, Egypt.

[‡] These authors contributed equally

**** Correspondence:** Professor Robert Capon

Tel +61 7 3346 2979. Facsimile +61 7 3346 2090. Email: r.capon@uq.edu.au

Table of content

Table of content ... i

List of figures ... iii

List of tables ... viii

List of schemes ... viii

1 General experimental ... 1

2 *Chrysosporium* sp. CMB-F214 media MATRIX study ... 2

3 Analytical precursor directed (nicotinate) feeding study ... 4

4 M2 agar scale up culture and production of chrysosporazine Q (15) ... 5

5 *Chrysosporium* sp. CMB-F214 culture supplemented with sodium nicotinate leading to amplified production of the minor natural products, azachrysosporazines 1–6, new chrysosporazines 7–9 and spirochrysosporazine A (10) ... 5

6 Spectroscopic characterization of metabolites ... 6

6.1 Azachrysosporazine A1 (1) ... 6

6.2 Azachrysosporazine A2 (2) ... 9

6.3 Azachrysosporazine B1 (3) ... 13

6.4 Azachrysosporazine C1 (4) ... 17

6.5 Azachrysosporazine C2 (5) ... 21

6.6 Azachrysosporazine D1 (6) ... 25

6.7 chrysosporazine N (7) ... 26

6.8 Chrysosporazine O (8) ... 30

6.9 Chrysosporazine P (9) ... 34

6.10 Spirochrysosporazine A (10) ... 35

6.11 Chrysosporazine Q (15) ... 39

7 Comparative NMR analysis of natural chrysosporazines to corresponding azachrysosporazines ... 43

8 CD spectra for chrysosporazines A–D (11–14) and 1–11 ... 47

9 Acid hydrolysis of azachrysosporazine D1 (6) and chrysosporazine P (9) 50
9.1 Azachrysosporazine D1 (6) 50
9.2 Chrysosporazine P (9) 50
10 UPLC-QTOF-SIE study 52
11 Antimicrobial assay 53
12 References 53

List of figures

Figure S1. Analytical cultivation of CMB-F214 under different conditions in microbioreactors (MATRIX study). ... 2

Figure S2. UPLC-DAD (210 nm) chromatograms for cultivation of CMB-F214 on different culture conditions (MATRIX) and production of chrysosporazines A–D (blue peaks), new azachrysosporazies (red peaks) and new chrysosporazine Q (15, orange peak) across different media: (A) M1; (B) M2; (C) ISP-2; (D) IMA; (E) CG; (F) GY; (G) R2YE; (H) YES; (I) PD; (J) PY; (K) TS; (L) SD, each media under different conditions: (i) solid agar; (ii) static broth; (iii) shaken broth, (iv) media blank, *internal calibrant. ... 3

Figure S3. UPLC-DAD (210 nm) of CMB-F214 cultures on (i) solid rice and (ii–vi) static M1 broth and M1 agar in absence and presence of sodium nicotinate, and production of new azachrysosporazines 1–6 (red peaks) and their corresponding known chrysosporazines 11–14 (blue peaks). ii) M1 broth culture; iii) M1 broth culture supplemented with sodium nicotinate (4 mg/mL); iv) M1 broth culture supplemented with sodium nicotinate (2 mg/mL); v) M1 agar culture; vi) M1 agar culture supplemented with sodium nicotinate (2 mg/mL), and vii) M1 media blank. Top trace: UV-vis spectra of 1–6 and 11–14. ... 4

Figure S4. ^{1}H NMR (600 MHz, DMSO-d_6) spectrum for azachrysosporazine A1 (1) ... 7

Figure S5. ^{13}C NMR (150 MHz, DMSO-d_6) spectrum for azachrysosporazine A1 (1) ... 7

Figure S6. ROESY NMR (600 MHz, DMSO-d_6) spectrum for azachrysosporazine A1 (1) ... 8

Figure S7. HMBC NMR (600 MHz, DMSO-d_6) spectrum for azachrysosporazine A1 (1) ... 8

Figure S8. ^{1}H NMR (600 MHz, DMSO-d_6) spectrum for azachrysosporazine A2 (2) ... 10

Figure S9. ^{13}C NMR (150 MHz, DMSO-d_6) spectrum for azachrysosporazine A2 (2) ... 10

Figure S10. ^{1}H NMR (600 MHz, DMSO-d_6) spectra for a) chrysosporazine A (11), b) azachrysosporazine A1 (1) and c) azachrysosporazine A2 (2) ... 11

Figure S11. ROESY NMR (600 MHz, DMSO-d_6) spectrum for azachrysosporazine A2 (2) 11

Figure S12. HMBC NMR (600 MHz, DMSO-d_6) spectrum for azachrysosporazine A2 (2) . 12

Figure S13. ^{1}H NMR (600 MHz, DMSO-d_6) spectrum for azachrysosporazine B1 (3) (major rotamer). ... 14

Figure S14. ^{13}C NMR (150 MHz, DMSO-d_6) spectrum for azachrysosporazine B1 (3) (major rotamer). 14

Figure S15. Expanded HSQC NMR (600 MHz, DMSO-d_6) spectrum (part 1) for azachrysosporazine B1 (3), major rotamer (labelled black); minor rotamer (labelled green). 15

Figure S16. Expanded HSQC NMR (600 MHz, DMSO-d_6) spectrum (part 2) for azachrysosporazine B1 (3), major rotamer (labelled black); minor rotamer (labelled green). 15

Figure S17. ROESY NMR (600 MHz, DMSO-d_6) spectrum for azachrysosporazine B1 (3), major rotamer (labelled black); minor rotamer (labelled green). 16

Figure S18. HMBC NMR (600 MHz, DMSO-d_6) spectrum for azachrysosporazine B1 (3).. 16

Figure S19. ^{1}H NMR (600 MHz, DMSO-d_6) spectrum for azachrysosporazine C1 (4) (major rotamer) 18

Figure S20. ^{13}C NMR (150 MHz, DMSO-d_6) spectrum for azachrysosporazine C1 (4) (major rotamer). 18

Figure S21. Expanded HSQC NMR (600 MHz, DMSO-d_6) spectrum (part 1) for azachrysosporazine C1 (4), major rotamer (labelled black); minor rotamer (labelled green). 19

Figure S22. Expanded HSQC NMR (600 MHz, DMSO-d_6) spectrum (part 2) for azachrysosporazine C1 (4), major rotamer (labelled black); minor rotamer (labelled green). 19

Figure S23. ROESY NMR (600 MHz, DMSO-d_6) spectrum for azachrysosporazine C1 (4), major rotamer (labelled black); minor rotamer (labelled green). 20

Figure S24. HMBC NMR (600 MHz, DMSO-d_6) spectrum for azachrysosporazine C1 (4).. 20

Figure S25. ^{1}H NMR (600 MHz, DMSO-d_6) spectrum for azachrysosporazine C2 (5) (major rotamer). * residual MeOH solvent. 22

Figure S26. ^{13}C NMR (150 MHz, DMSO-d_6) spectrum for azachrysosporazine C2 (5) (major rotamer). * residual MeOH solvent. 22

Figure S27. Expanded HSQC NMR (600 MHz, DMSO-d_6) spectrum (part 1) for azachrysosporazine C2 (5), major rotamer (labelled black); minor rotamer (labelled green). 23

Figure S28. Expanded HSQC NMR (600 MHz, DMSO-d_6) spectrum (part 2) for azachrysosporazine C2 (5), major rotamer (labelled black); minor rotamer (labelled green). 23

Figure S29. ROESY NMR (600 MHz, DMSO-d_6) spectrum for azachrysosporazine C2 (5), major rotamer (labelled black); minor rotamer (labelled green). 24

Figure S 30. HMBC NMR (600 MHz, DMSO-d_6) spectrum for azachrysosporazine C2 (5). 24

Figure S31. ^{1}H NMR (600 MHz, DMSO-d_6) spectrum for azachrysosporazine D1 (6). 25

Figure S32. ^{1}H NMR (600 MHz, DMSO-d_6) spectra for a) chrysosporazine D and b) azachrysosporazine D1 (6) 25

Figure S33. ^{1}H NMR (600 MHz, DMSO-d_6) spectrum for chrysosporazine N (7) (major rotamer). 27

Figure S34. ^{13}C NMR (150 MHz, DMSO-d_6) spectrum for chrysosporazine N (7) (major rotamer). 27

Figure S35. Expanded HSQC NMR (600 MHz, DMSO-d_6) spectrum (part 1) for chrysosporazine N (7), major rotamer (labelled black); minor rotamer (labelled green)........ 28

Figure S36. Expanded HSQC NMR (600 MHz, DMSO-d_6) spectrum (part 2) for chrysosporazine N (7), major rotamer (labelled black); minor rotamer (labelled green)........ 28

Figure S37. ROESY NMR (600 MHz, DMSO-d_6) spectrum for chrysosporazine N (7), major rotamer (labelled black); minor rotamer (labelled green). 29

Figure S38. HMBC NMR (600 MHz, DMSO-d_6) spectrum for chrysosporazine N (7)......... 29

Figure S39. ^{1}H NMR (600 MHz, DMSO-d_6) spectrum for chrysosporazine O (8) (major rotamer). 31

Figure S40. ^{13}C NMR (150 MHz, DMSO-d_6) spectrum for chrysosporazine O (8) (major rotamer). 31

Figure S41. Expanded HSQC NMR (600 MHz, DMSO-d_6) spectrum (part 1) for chrysosporazine O (8), major rotamer (labelled black); minor rotamer (labelled green)........ 32

Figure S42. Expanded HSQC NMR (600 MHz, DMSO-d_6) spectrum (part 2) for chrysosporazine O (8), major rotamer (labelled black); minor rotamer (labelled green)........ 32

Figure S43. ROESY NMR (600 MHz, DMSO-d_6) spectrum for chrysosporazine O (8), major rotamer (labelled black); minor rotamer (labelled green). 33

Figure S44. HMBC NMR (600 MHz, DMSO-d_6) spectrum for chrysosporazine O (8)......... 33

Figure S45. ^{1}H NMR (600 MHz, DMSO-d_6) spectrum for chrysosporazine P (9)................ 34

Figure S46. ^{1}H NMR (600 MHz, DMSO-d_6) spectra for a) chrysosporazine D (14) and b) chrysosporazine P (9). 34

Figure S47. ^{1}H NMR (600 MHz, DMSO-d_6) spectrum for spirochrysosporazine A (10). 36

Figure S48. ^{13}C NMR (150 MHz, DMSO-d_6) spectrum for spirochrysosporazine A (10). 36

Figure S49. Expanded HSQC NMR (600 MHz, DMSO-d_6) spectrum (part 1) for spirochrysosporazine A (10), major rotamer (labelled black); minor rotamer (labelled green). 37

Figure S50. Expanded HSQC NMR (600 MHz, DMSO-d_6) spectrum (part 2) for spirochrysosporazine A (10), major rotamer (labelled black); minor rotamer (labelled green). 37

Figure S51. ROESY NMR (600 MHz, DMSO-d_6) spectrum for spirochrysosporazine A (10), major rotamer (labelled black); minor rotamer (labelled green). 38

Figure S52. HMBC NMR (600 MHz, DMSO-d_6) spectrum for spirochrysosporazine (10)... 38

Figure S53. ^{1}H NMR (600 MHz, DMSO-d_6) spectrum for chrysosporazine Q (15). 40

Figure S54. ^{13}C NMR (150 MHz, DMSO-d_6) spectrum for chrysosporazine Q (15). 40

Figure S55. Expanded HSQC NMR (600 MHz, DMSO-d_6) spectrum (part 1) for chrysosporazine Q (15), major rotamer (labelled black); minor rotamer (labelled green)...... 41

Figure S56. Expanded HSQC NMR (600 MHz, DMSO-d_6) spectrum (part 2) for chrysosporazine Q (15), major rotamer (labelled black); minor rotamer (labelled green)...... 41

Figure S57. ROESY NMR (600 MHz, DMSO-d_6) spectrum for chrysosporazine Q (15), major rotamer (labelled black); minor rotamer (labelled green). 42

Figure S58. HMBC NMR (600 MHz, DMSO-d_6) spectrum for chrysosporazine Q (15)....... 42

Figure S59. CD (top) and UV (bottom) spectra for chrysosporazine A (11), azachrysosporazine A1 (1) and azachrysosporazine A2 (2) in MeOH (0.05 g%). 47

Figure S60. CD (top) and UV (bottom) spectra for chrysosporazine B (12), azachrysosporazine B1 (3), chrysosporazine N (7) and chrysosporazine O (8) in MeOH (0.05 g%). 47

Figure S61. CD (top) and UV (bottom) spectra for chrysosporazine C (13), azachrysosporazine C1 (4) and azachrysosporazine C2 (5) in MeOH (0.05 g%). 48

Figure S62. CD (top) and UV (bottom) spectra for chrysosporazine D (14), azachrysosporazine D1 (6) and chrysosporazine P (9) in MeOH (0.05 g%)...48

Figure S63. CD (top) and UV (bottom) spectra for chrysosporazines Q (15) and spirochrysosporazine A (10) in MeOH (0.05 g%). 49

Figure S64. UPLC-DAD (210 nm) of acid hydrolysis of azachrysosporazine D1 (6). (i) purified 6; acid hydrolysis of 6 at (ii) 12 hr, (iii) 24 hr and (iv) 36 hr; (v) acid hydrolysis of 6 at 12 hr co-injected with purified 16 obtained from acid hydrolysis of 14; (vi) purified 16................50

Figure S65. UPLC-DAD (210 nm) of acid hydrolysis of chrysosporazine P (9). (i) purified 9; acid hydrolysis of 9 at (ii) 12 hr, (iii) 24 hr and (iv) 36 hr. 51

Figure S66. UPLC-QTOF-SIE analysis of an M1 agar culture of CMB-F214 (without sodium nicotinate feeding), with SIE at i) *m/z* 394, 1–2; ii) *m/z* 500, 3; iii) *m/z* 486, 4–5; iv) *m/z* 488, 6; v) *m/z* 501, 7–8; vi) *m/z* 489, 9; vii) *m/z* 371, 10, along with a co-injection of authentic 1–4 and 6–10. * refers to $[M+H+1]^+$ of corresponding natural chrysosporazines A–D (11–14)..........52

Figure S67. Growth inhibitory activity of 1-15...53

List of tables

Table S1. 1D and 2D NMR (600 MHz, DMSO-d_6) data for azachrysosporazine A1 (1) 6

Table S2. 1D and 2D NMR (600 MHz, DMSO-d_6) data for azachrysosporazine A2 (2) 9

Table S3. 1D and 2D NMR (600 MHz, DMSO-d_6) data for azachrysosporazine B1 (3) (major rotamer) 13

Table S4. 1D and 2D NMR (600 MHz, DMSO-d_6) data for azachrysosporazine C1 (4) (major rotamer) 17

Table S5. 1D and 2D NMR (600 MHz, DMSO-d_6) data for azachrysosporazine C2 (5) (major rotamer) 21

Table S6. 1D and 2D NMR (600 MHz, DMSO-d_6) data for chrysosporazine N (7) (major rotamer) 26

Table S7. 1D and 2D NMR (600 MHz, DMSO-d_6) data for chrysosporazine O (8) (major rotamer) 30

Table S8. 1D and 2D NMR (600 MHz, DMSO-d_6) data for spirochrysosporazine A (10) (major rotamer) 35

Table S9. 1D and 2D NMR (600 MHz, DMSO-d_6) data for chrysosporazine Q (15) (major rotamer) 39

Table S10. Chrysosporazine A (11), azachrysosporazines A1 (1) and A2 (2) 43

Table S11. Chrysosporazine B (12) and azachrysosporazine B1 (3) 44

Table S12. Chrysosporazine C (13), azachrysosporazines C1 (4) and C2 (5) 45

Table S13. chrysosporazine N (7) and chrysosporazine O (8) 46

List of schemes

Scheme S1. Isolation scheme of chrysosporazine Q (15): a) trituration of crude extract with *n*-hexane (-1) to obtain defatted crude extract (-2); b) gel chromatography: Sephadex LH-20 (2.5 cm × 70 cm) for 200 mg, elution with MeOH (100 %); c) semi-preparative HPLC purification for 20 mg. 5

Scheme S2. Isolation scheme of azachrysosporazines 1–6, chrysosporazines 7–9 and spirochrysosporazine A (10): a) trituration of crude extract with *n*-hexane (-1) and DCM (-2); b) preparative HPLC fractionation for 380 mg; c) semi-preparative HPLC purification. 5

Scheme S3. Acid hydrolysis schemes of azachrysosporazine D1 (6) in comparison to chrysosporazine D (14)..50

Scheme S4. Acid hydrolysis scheme of chrysosporazine P (9) in comparison to chrysosporazine D (14)..51

1 General experimental

Chemicals were purchased from Sigma-Aldrich or Merck unless otherwise specified. Analytical-grade solvents were used for solvent extractions. Solvents used for HPLC, UPLC and HPLC-MS purposes were of HPLC grade supplied by Labscan or Sigma-Aldrich and filtered/degassed through 0.45 μm polytetrafluoroethylene (PTFE) membrane prior to use. Deuterated solvents were purchased from Cambridge Isotopes (Tewksbury, MA, USA).

Microorganism was manipulated under sterile conditions providing by a Laftech class II biological safety cabinet and incubated in either a MMM Friocell incubators (Lomb Scientific, NSW, Australia) or an Innova 42R incubator shaker (John Morris, NSW, Australia) with temperature set at 26.5 °C.

Semi-preparative and preparative HPLCs were performed using Agilent 1100 series HPLC instruments with corresponding detectors, fraction collectors and software inclusively. UPLC chromatograms were obtained on Agilent 1290 infinity UPLC system equipped with diode array multiple wavelength detector (Zorbax C_8 RRHD 1.8 μm column, 50 × 2.1 mm, eluting with 0.417 mL/min 90% H_2O/MeCN to 100% MeCN (with isocratic 0.01% TFA modifier) over 2.50 min). UPLC-QTOF analysis was performed on UPLC-QTOF instrument comprising an Agilent 1290 Infinity II UHPLC (Zorbax C_8 RRHD 1.8 μm column, 50 × 2.1 mm, eluting with 0.417 mL/min of gradient elution of 90% H_2O/MeCN to 100% MeCN over 2.5 min (with isocratic 0.1% formic acid modifier) coupled to an Agilent 6545 Q-TOF.

Chiroptical measurements ($[\alpha]_D$) were obtained on a JASCO P-1010 polarimeter in a 100 × 2 mm cell at specified temperatures. Nuclear magnetic resonance (NMR) spectra were acquired on a Bruker Avance 600 MHz spectrometer with either a 5 mm PASEL 1H/D-13C Z-Gradient probe or 5 mm CPTCI 1H/19F-13C/15N/DZ-Gradient cryoprobe, controlled by TopSpin 2.1 software. In all cases spectra were acquired at 25 °C in DMSO-d_6 with referencing to residual ^{1}H or ^{13}C signals (δ_H 2.50 and δ_C 39.51 ppm) in the deuterated solvent. High-resolution ESIMS spectra were obtained on a Bruker micrOTOF mass spectrometer by direct injection in MeOH at 3 μL/min using sodium formate clusters as an internal calibrant.

2 *Chrysosporium* sp. CMB-F214 media MATRIX study

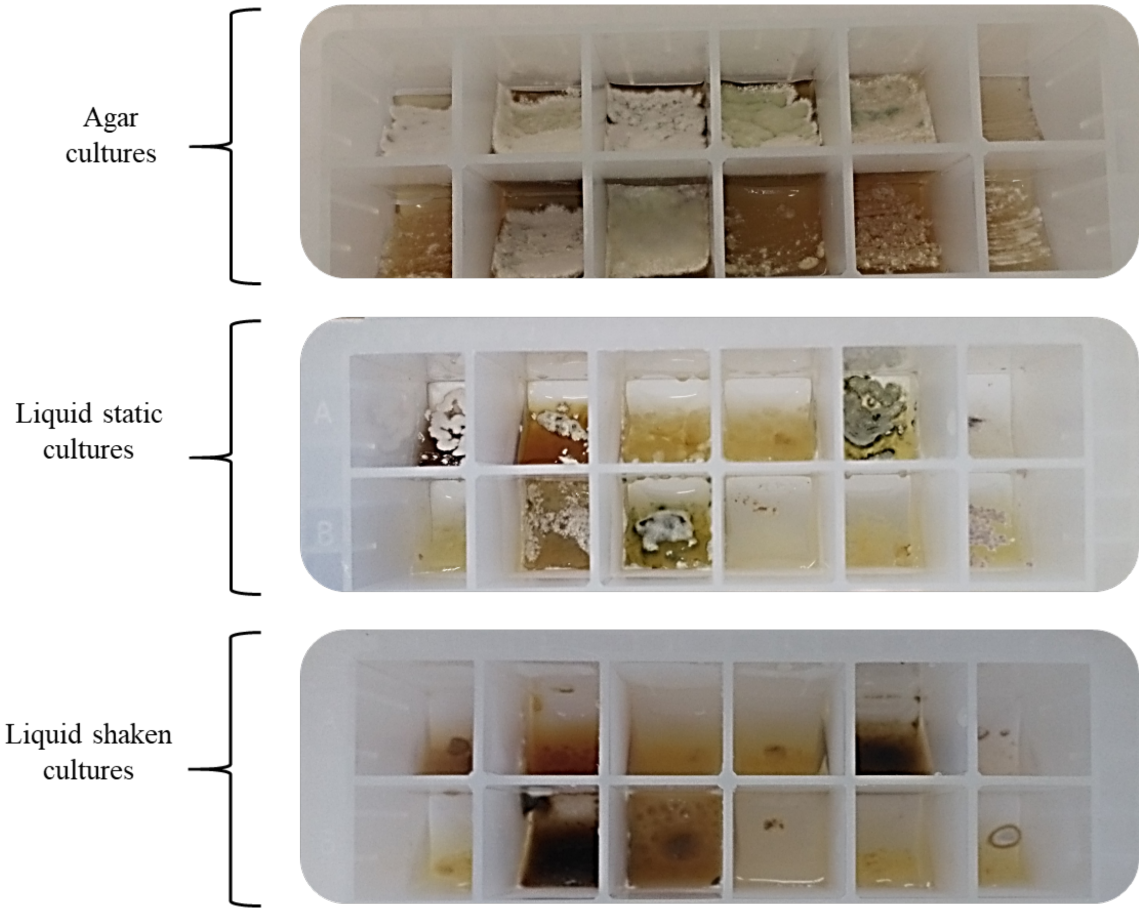

Figure S1. Analytical cultivation of CMB-F214 under different conditions in microbioreactors (MATRIX study).

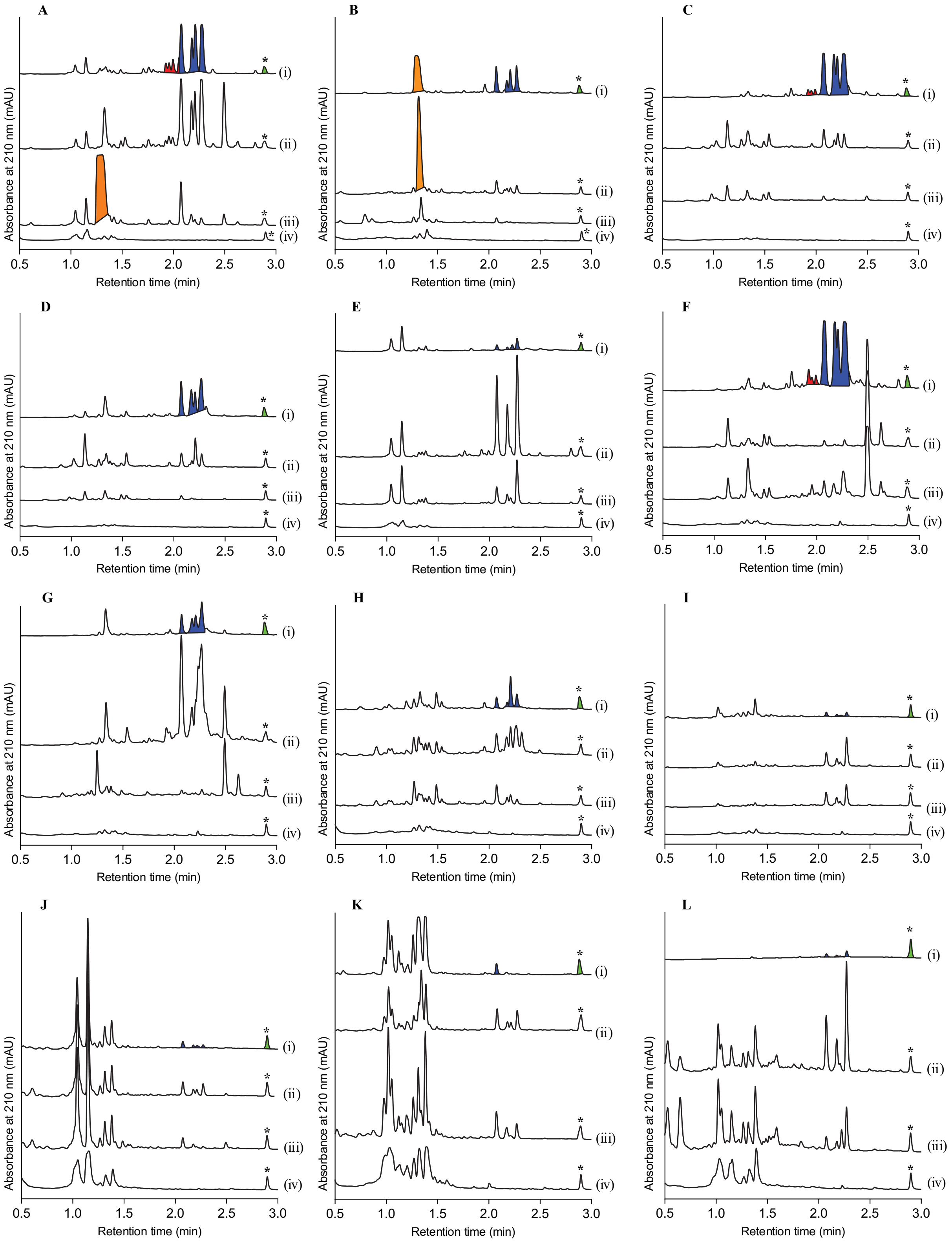

Figure S2. UPLC-DAD (210 nm) chromatograms for cultivation of CMB-F214 on different culture conditions (MATRIX) and production of chrysosporazines A–D (blue peaks), new azachrysosporazies (red peaks) and new chrysosporazine Q (**15**, orange peak) across different media: (A) M1; (B) M2; (C) ISP-2; (D) IMA; (E) CG; (F) GY; (G) R2YE; (H) YES; (I) PD; (J) PY; (K) TS; (L) SD, each media under different conditions: (i) solid agar; (ii) static broth; (iii) shaken broth, (iv) media blank, *internal calibrant.

3 Analytical precursor directed (nicotinate) feeding study

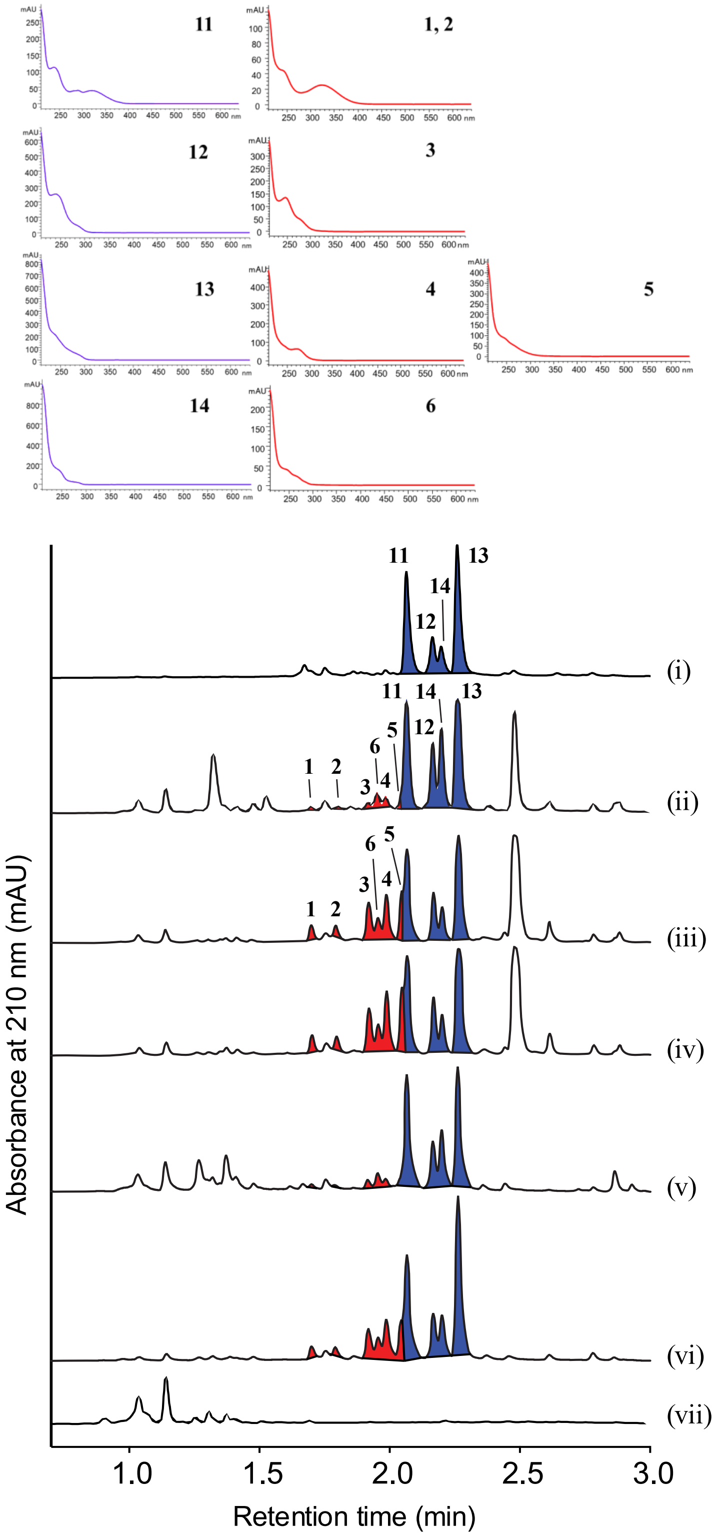

Figure S3. UPLC-DAD (210 nm) of CMB-F214 cultures on (i) solid rice and (ii–vi) static M1 broth and M1 agar in absence and presence of sodium nicotinate, and production of new azachrysosporazines **1–6** (red peaks) and their corresponding known chrysosporazines **11–14** (blue peaks). ii) M1 broth culture; iii) M1 broth culture supplemented with sodium nicotinate (4 mg/mL); iv) M1 broth culture supplemented with sodium nicotinate (2 mg/mL); v) M1 agar culture; vi) M1 agar culture supplemented with sodium nicotinate (2 mg/mL), and vii) M1 media blank. Top trace: UV-vis spectra of **1–6** and **11–14**.

4 M2 agar scale up culture and production of chrysosporazine Q (15)

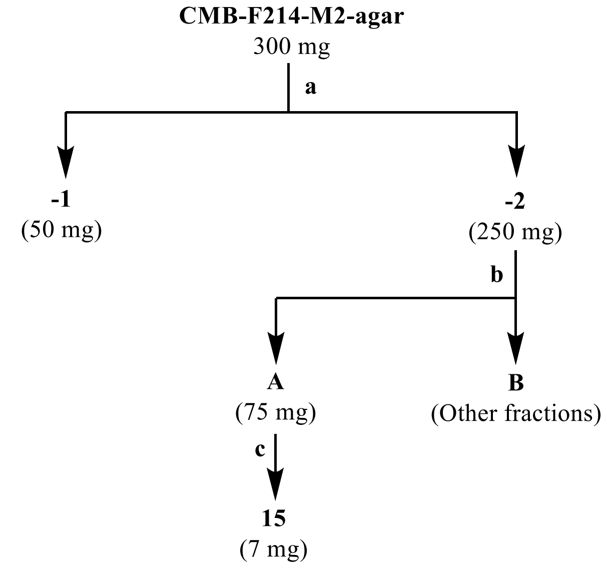

Scheme S1. Isolation scheme of chrysosporazine Q (**15**): a) trituration of crude extract with *n*-hexane (-1) to obtain defatted crude extract (-2); b) gel chromatography: Sephadex LH-20 (2.5 cm × 70 cm) for 200 mg, elution with MeOH (100 %); c) semi-preparative HPLC purification for 20 mg.

5 *Chrysosporium* sp. CMB-F214 culture supplemented with sodium nicotinate leading to amplified production of the minor natural products, azachrysosporazines 1–6, new chrysosporazines 7–9 and spirochrysosporazine A (10)

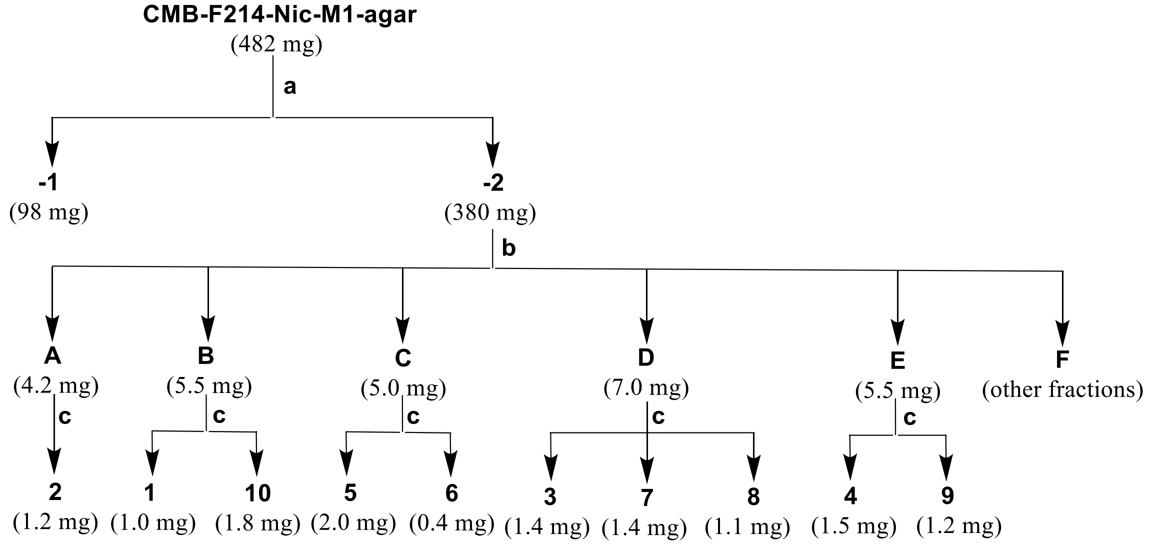

Scheme S2. Isolation scheme of azachrysosporazines **1–6**, chrysosporazines **7–9** and spirochrysosporazine A (**10**): a) trituration of crude extract with *n*-hexane (-1) and DCM (-2); b) preparative HPLC fractionation for 380 mg; c) semi-preparative HPLC purification.

6 Spectroscopic characterization of metabolites

6.1 Azachrysosporazine A1 (1)

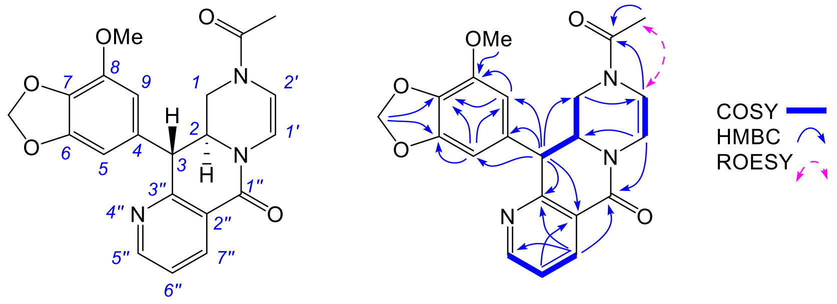

Table S1. 1D and 2D NMR (600 MHz, DMSO-d_6) data for azachrysosporazine A1 (**1**)

Position	δ_H, mult (*J* in Hz)	δ_C	COSY	^{1}H-^{13}C HMBC	ROESY
1	a 4.26, ddd (*13.3,3.0, 1.2*)	42.3	1b	2′	1b
	b 2.99, dd (*13.3, 9.8*)		1a, 2	2	1a, 3
2	4.47, ddd (*12.3, 9.8, 3.0*)	56.9	1b, 3	---	1b, 5, 9
3	4.51, d (*12.3*)	48.8	2	1, 2, 4, 5, 9, 2″, 3″	1b, 5, 9
4	---	132.8	---	---	---
5	6.57, d (*1.4*)	103.4	---	3, 6, 7, 9	2/3
6	---	148.5	---	---	---
7	---	134.0	---	---	---
8	---	143.1	---	---	---
9	6.65, d (*1.4*)	110.1	---	3, 5, 7, 8	2/3, 8-OCH$_3$
1′	6.80, d (*6.8*)	106.7	2′	2, 2′	---
2′	6.55, dd (*6.8, 1.2*)	112.6	1′	1, 1′, 1-NCO	1-NCOCH$_3$
1″	---	158.5	---	---	---
2″	---	123.2	---	---	---
3″	---	158.6	---	---	---
**N**					
5″	8.56, dd (*4.8, 1.8*)	152.4	6″	6″, 7″	---
6″	7.43, ddd (*7.8, 4.8, 0.8*)	122.9	5″, 7″	2″, 5″	---
7″	8.26, dd (*7.8, 1.8*)	135.4	6″	1″, 3″, 5″	---
1-NCO	---	166.7	---	---	---
1-NCOCH$_3$	2.10, s	20.8	---	2′, 1-NCO	2′
6-OCH$_2$	6.02/6.00, AB$_q$	101.2	---	6, 7	---
8-OCH$_3$	3.80, s	56.4	---	8	9

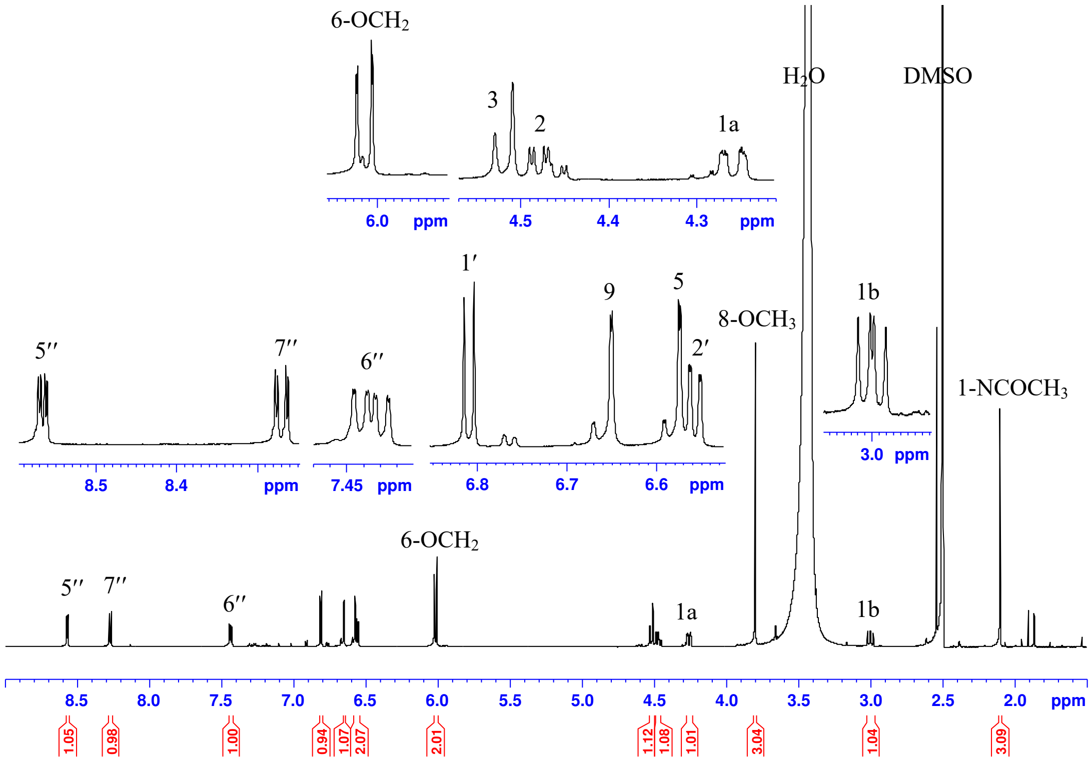

Figure S4. ^{1}H NMR (600 MHz, DMSO-d_6) spectrum for azachrysosporazine A1 (**1**)

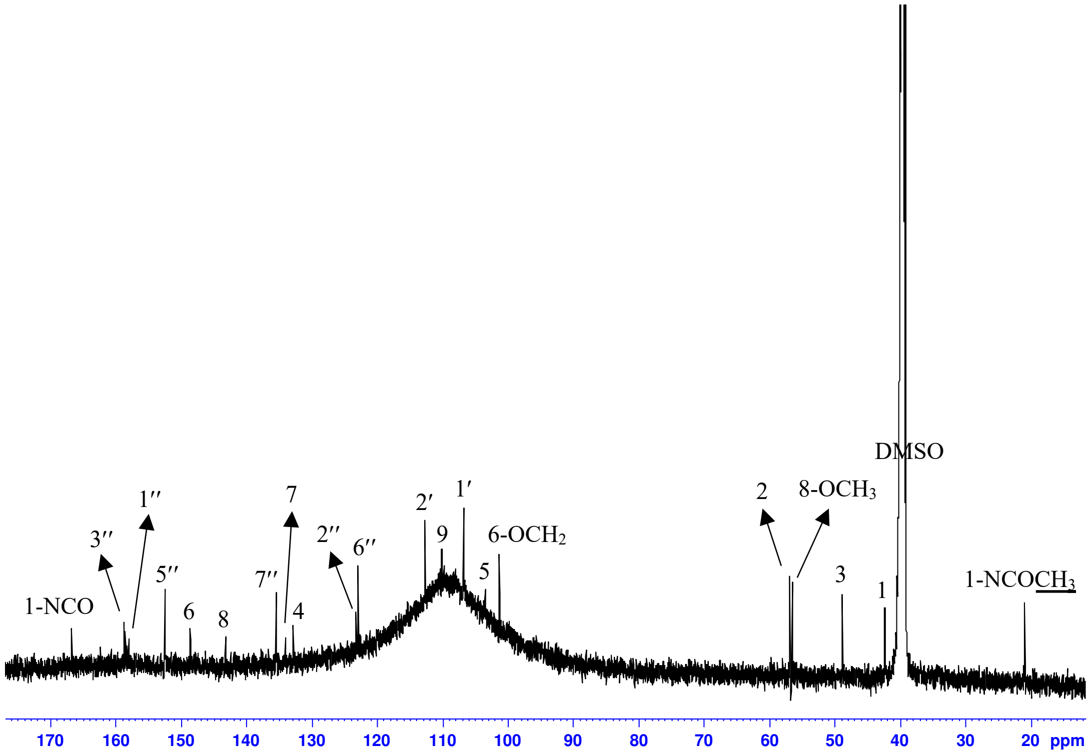

Figure S5. ^{13}C NMR (150 MHz, DMSO-d_6) spectrum for azachrysosporazine A1 (**1**)

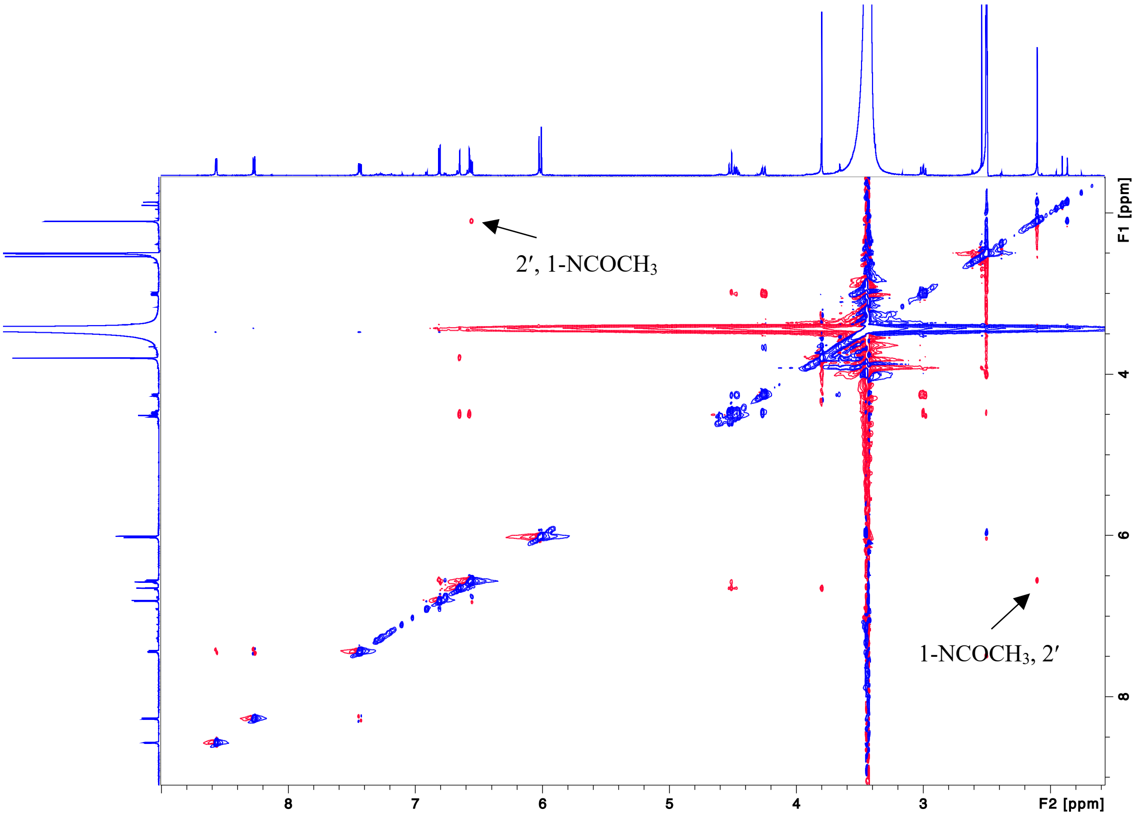

Figure S6. ROESY NMR (600 MHz, DMSO-d_6) spectrum for azachrysosporazine A1 (**1**)

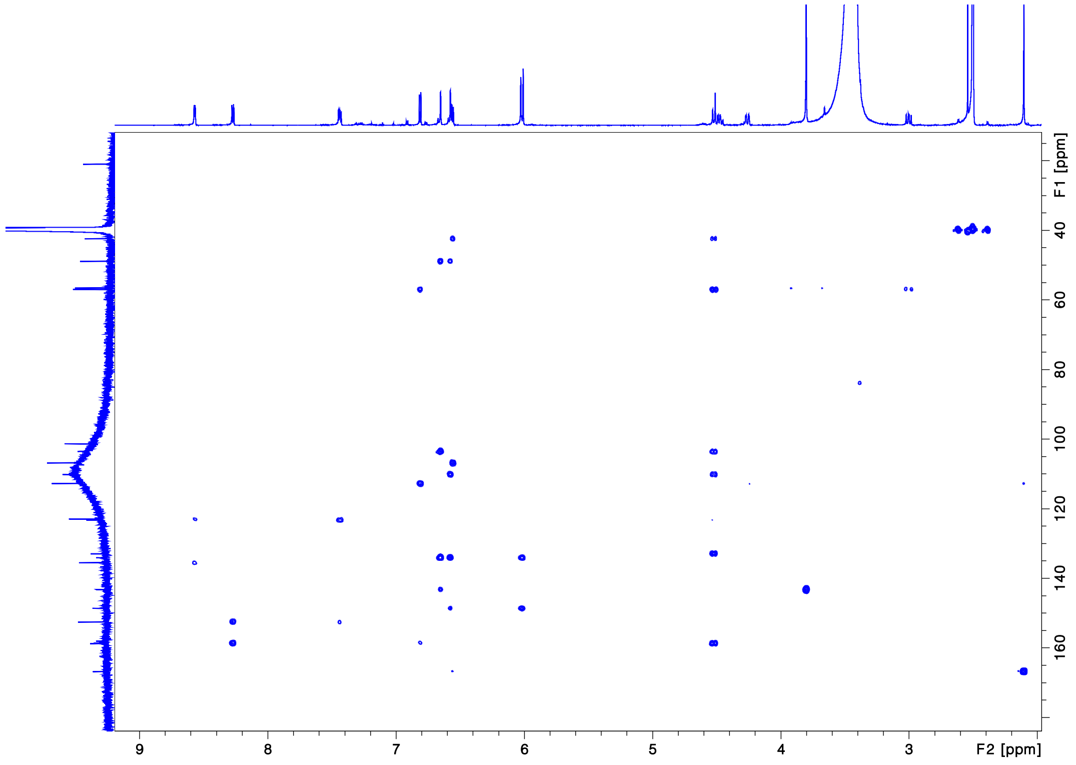

Figure S7. HMBC NMR (600 MHz, DMSO-d_6) spectrum for azachrysosporazine A1 (**1**)

6.2 Azachrysosporazine A2 (2)

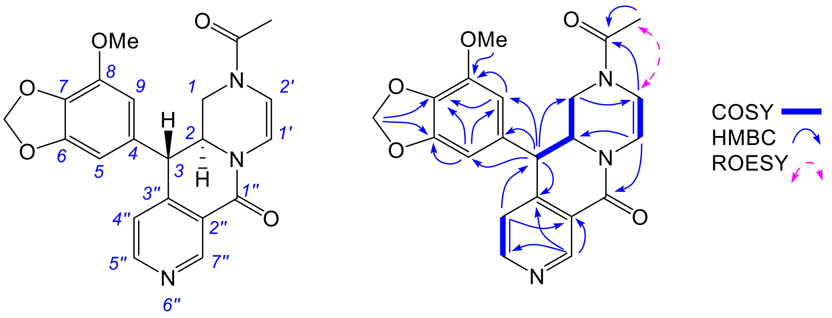

Table S2. 1D and 2D NMR (600 MHz, DMSO-d_6) data for azachrysosporazine A2 (**2**)

**Position**	δ_H, mult (*J* in Hz)	δ_C	COSY	^{1}H-^{13}C HMBC	ROESY
1	a 4.21, ddd (*13.1,3.0, 1.2*)	42.1	1b, 2	2′	1b, 2
	b 2.97, dd (*13.1, 9.8*)		1a, 2	2	1a, 3
2	4.49, ddd (*12.1, 9.8, 3.0*)	56.5[a]	1, 3	---	1a, 5, 9
3	4.46, d (*12.1*)	45.4	2	1, 2, 4, 5, 9, 3″	1b, 5, 9
4	---	131.1	---	---	---
5	6.68, d (*0.9*)	103.0[b]	---	3, 6, 7, 9	2, 3
6	---	149.0	---	---	---
7	---	134.6	---	---	---
8	---	143.6	---	---	---
9	6.74, d (*0.9*)	109.6[b]	---	3, 5, 7, 8	2, 3, 8-OCH_3
1′	6.81, d (*6.8*)	106.4	2′	2, 2′, 1″	---
2′	6.56, dd (*6.8, 1.2*)	112.7	1′	1, 1′, 1-NCO	1-$NCOCH_3$
1″	---	157.4	---	---	---
2″	---	123.1	---	---	---
3″	---	149.8	---	---	---
4″	6.66, d (*5.0*)	121.5	5″	3, 2″, 5″	5″
5″	8.59, d (*5.0*)	152.5	4″	3″, 4″, 7″	4″
**N**					
7″	9.03, s	147.9	---	2″, 3″, 5″	---
1-N<u>CO</u>	---	166.7	---	---	---
1-NCO<u>CH_3</u>	2.10, s	20.8	---	2′, 1-NCO	2′
6-OCH_2	6.06/6.05, AB_q	101.6	---	6, 7	---
8-OCH_3	3.81, s	56.5[a]	---	8	9

(a) signals are interchangeable within same letters, (b) signals determined from HMBC correlations

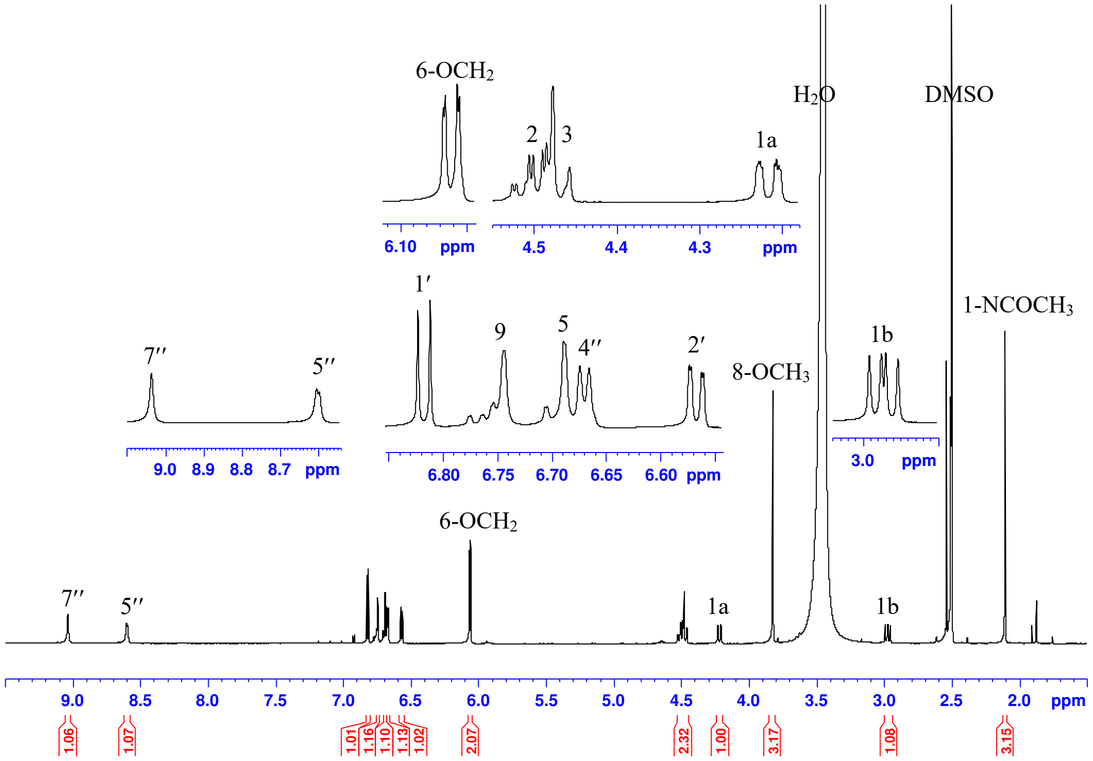

Figure S8. ^{1}H NMR (600 MHz, DMSO-d_6) spectrum for azachrysosporazine A2 (**2**)

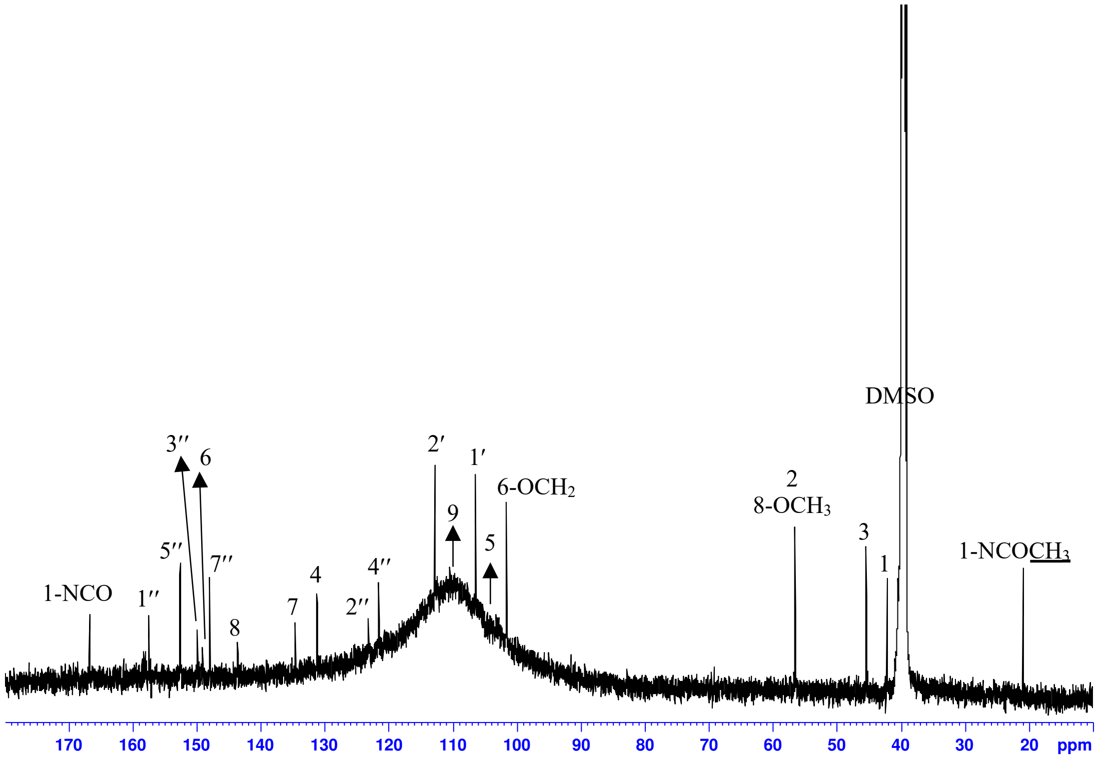

Figure S9. ^{13}C NMR (150 MHz, DMSO-d_6) spectrum for azachrysosporazine A2 (**2**)

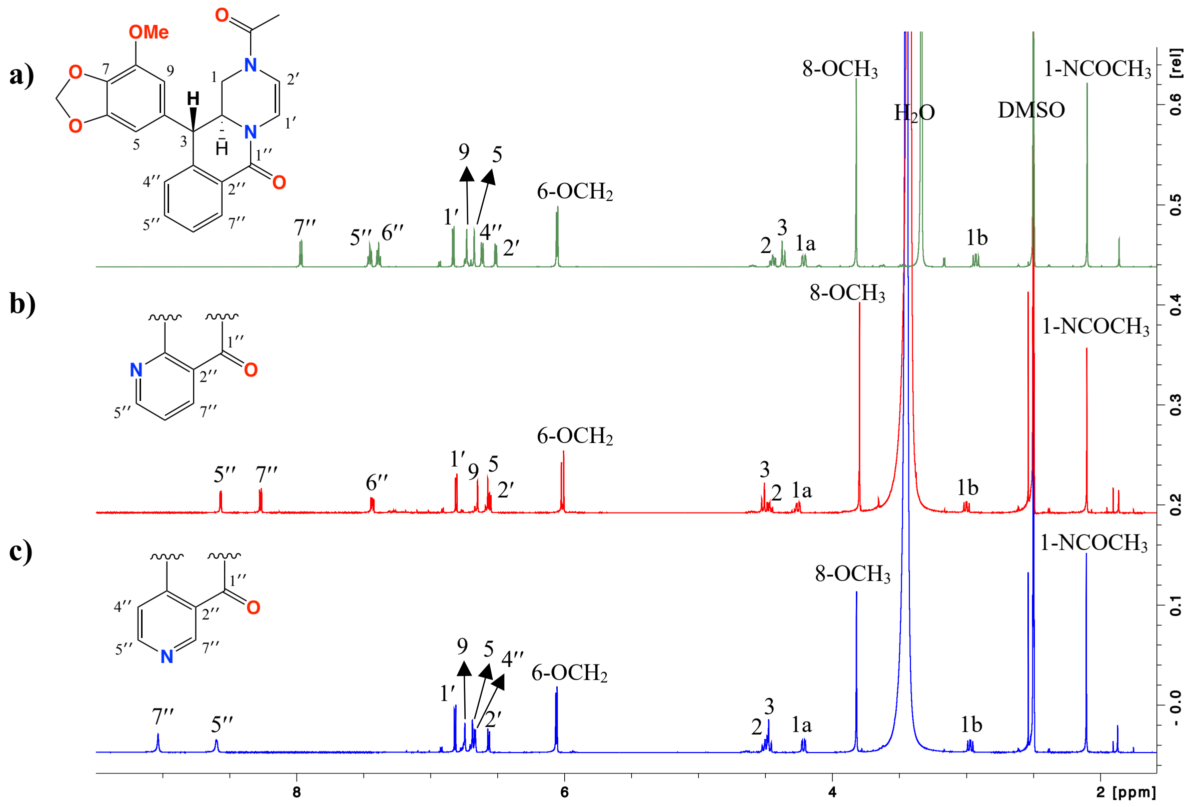

Figure S10. ^{1}H NMR (600 MHz, DMSO-d_6) spectra for a) chrysosporazine A (**11**), b) azachrysosporazine A1 (**1**) and c) azachrysosporazine A2 (**2**)

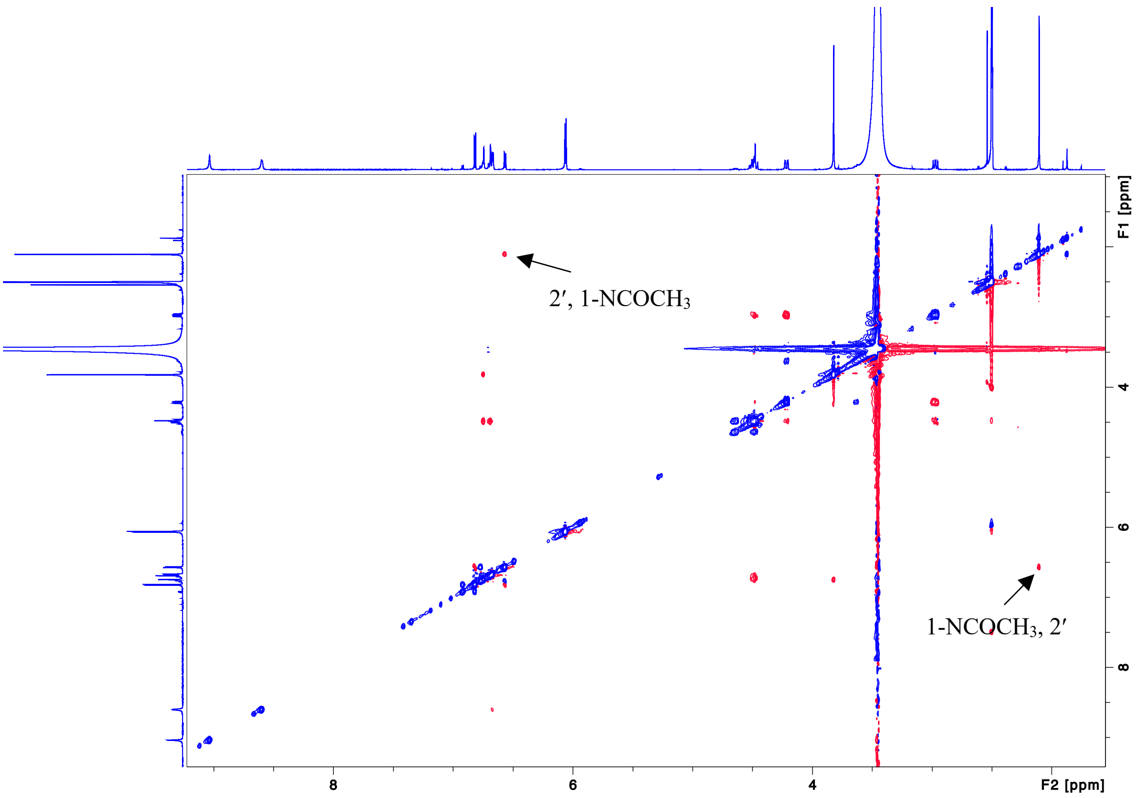

Figure S11. ROESY NMR (600 MHz, DMSO-d_6) spectrum for azachrysosporazine A2 (**2**)

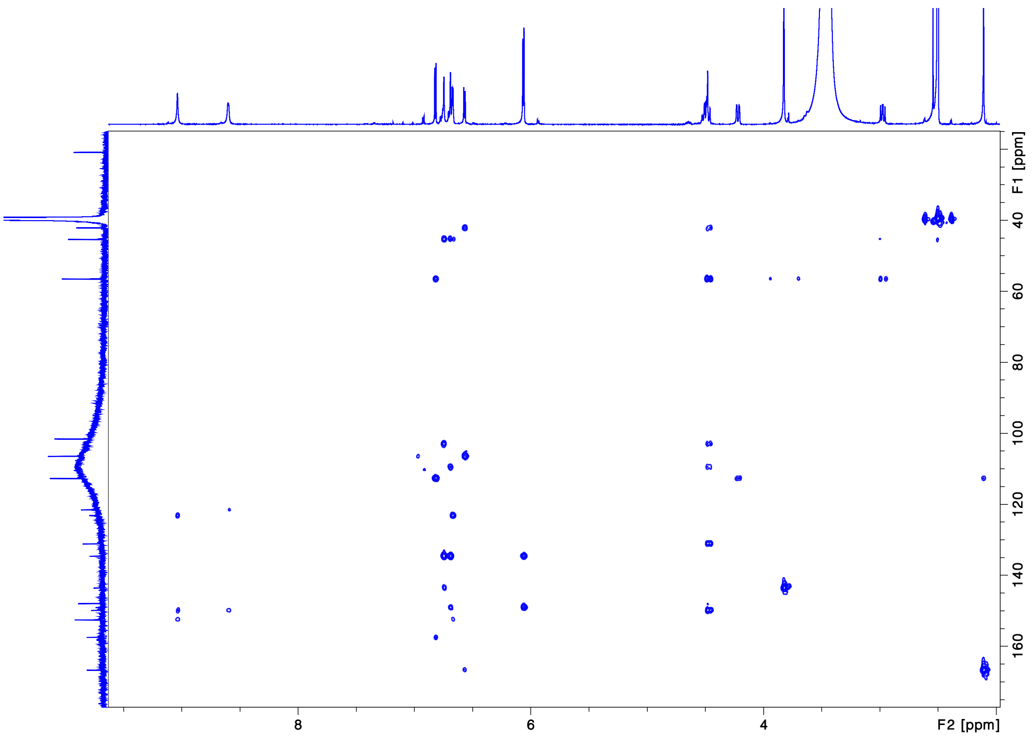

Figure S12. HMBC NMR (600 MHz, DMSO-d_6) spectrum for azachrysosporazine A2 (**2**)

6.3 Azachrysosporazine B1 (3)

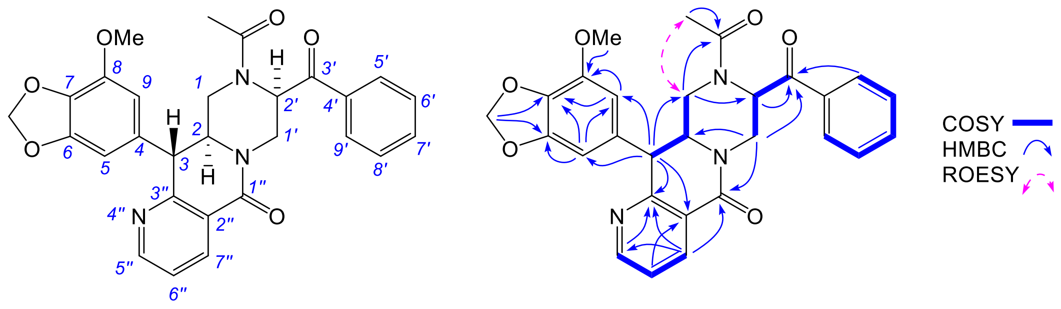

Table S3. 1D and 2D NMR (600 MHz, DMSO-d_6) data for azachrysosporazine B1 (**3**) (major rotamer)

**Position**	δ_H, mult (*J* in Hz)	δ_C	COSY	^{1}H-^{13}C HMBC	ROSEY
1	a 3.98, dd (*12.9, 3.8*)	47.3	1b, 2	2, 2′	2, 3, 1-NCOCH$_3$
	b 3.51[a]		1a, 2	---	---
2	4.21, ddd (*9.8, 5.4, 3.8*)	59.2	1b, 3	1	1a, 5, 9
3	4.46, d (*5.4*)	47.2	2	1, 2, 4, 5, 9, 2″, 3″	1a, 5, 9
4	---	135.3	---	---	---
5	6.31, d (*1.4*)	102.0	9	3, 6, 7, 9	2, 3
6	---	148.6	---	---	---
7	---	133.8	---	---	---
8	---	143.1	---	---	---
9	6.55, d (*1.4*)	108.6	---	3, 4, 5, 7, 8	2, 3, 8-OCH$_3$
1′	a 4.79, dd (*14.1, 1.3*)	42.6	1′b, 2′	2, 3′, 1″	1′b, 2′, 5′/9′
	b 3.51[a]		1′a, 2′	2, 2′, 3′, 1″	1′a
2′	5.89, dd (*5.7, 1.3*)	54.3	1′	1, 1′, 3′, 1-NCO	1′a, 1′b, 5′/9′
3′	---	197.6	---	---	---
4′	---	134.7	---	---	---
5′/ 9′	7.97, m	128.2	6′/ 8′	3′, 5′/ 9′, 6′/ 8′, 7′	1′a, 2′
6′/ 8′	7.56, m	128.9	5′/ 9′, 7′	4′, 5′/ 9′, 6′/ 8′	---
7′	7.68, m	133.5	6′/ 8′	5′/ 9′, 6′/ 8′	---
1″	---	161.9	---	---	---
2″	---	122.8	---	---	---
3″	---	158.1	---	---	---
**N**					
5″	8.64, dd (*4.7, 1.8*)	153.0	6″	3″, 6″, 7″	6″
6″	7.44, dd (*7.8, 4.7*)	123.1	5″, 7″	2″, 5″	5″
7″	8.18, dd (*7.8, 1.8*)	135.6	6″	1″, 3″, 5″	---
1-N<u>CO</u>	---	170.5	---	---	---
1-NCO<u>CH$_3$</u>	2.00, s	21.4	---	1, 1-NCO	1a
6-OCH$_2$	5.95/5.94, AB$_q$	101.3	---	6, 7	---
8-OCH$_3$	3.78, s	56.4	---	8	9

(a) overlapping resonances and obscured by solvent signal

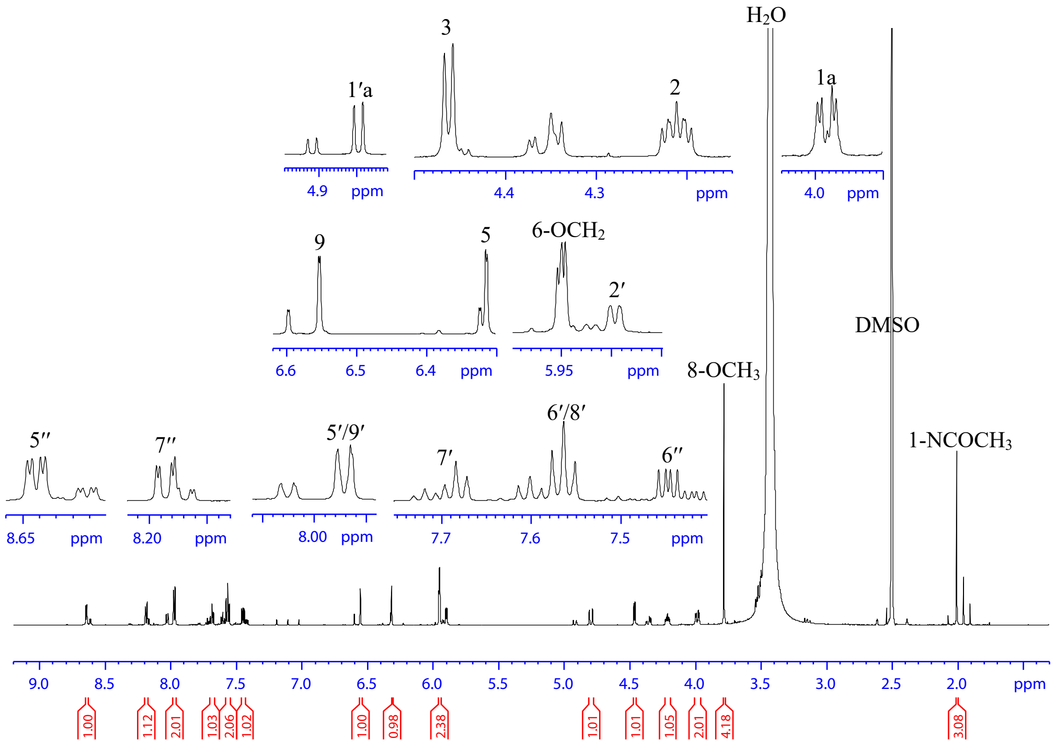

Figure S13. ^{1}H NMR (600 MHz, DMSO-d_6) spectrum for azachrysosporazine B1 (**3**) (major rotamer).

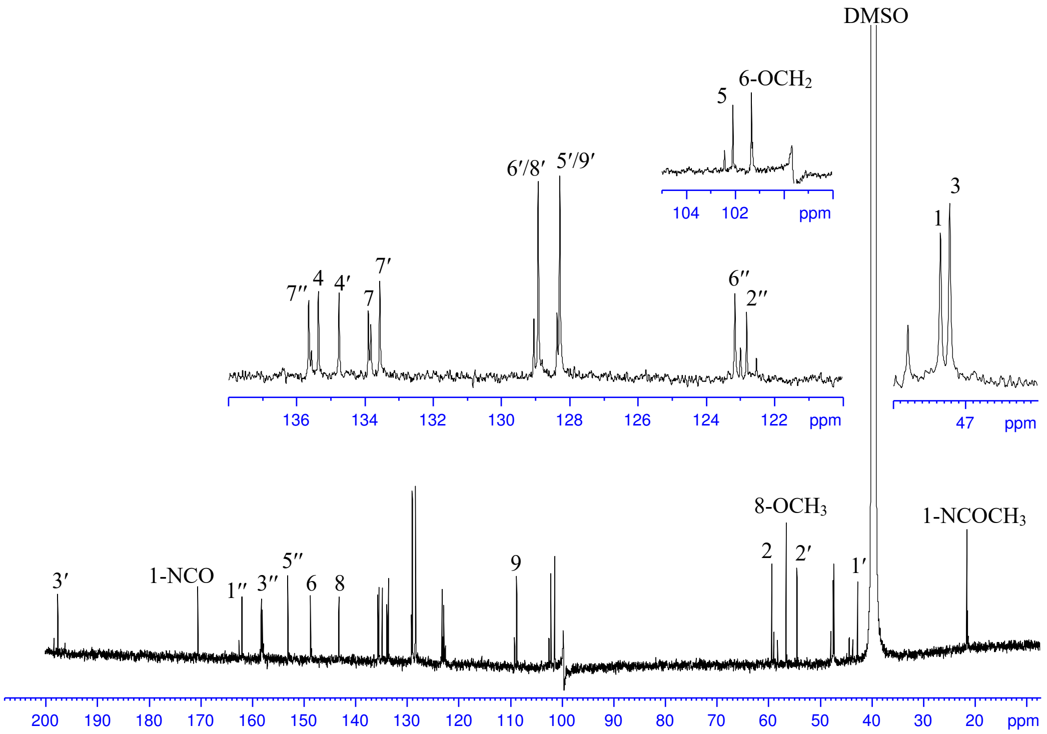

Figure S14. ^{13}C NMR (150 MHz, DMSO-d_6) spectrum for azachrysosporazine B1 (**3**) (major rotamer).

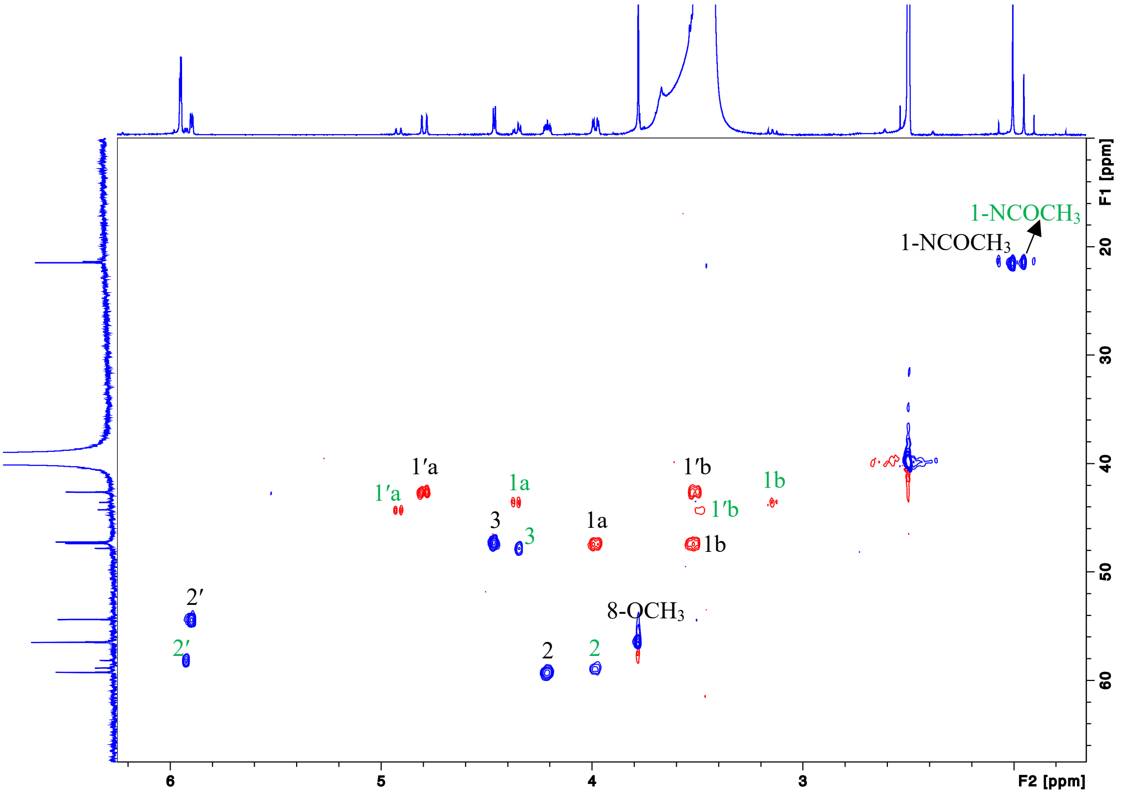

Figure S15. Expanded HSQC NMR (600 MHz, DMSO-d_6) spectrum (part 1) for azachrysosporazine B1 (**3**), major rotamer (labelled black); minor rotamer (labelled green).

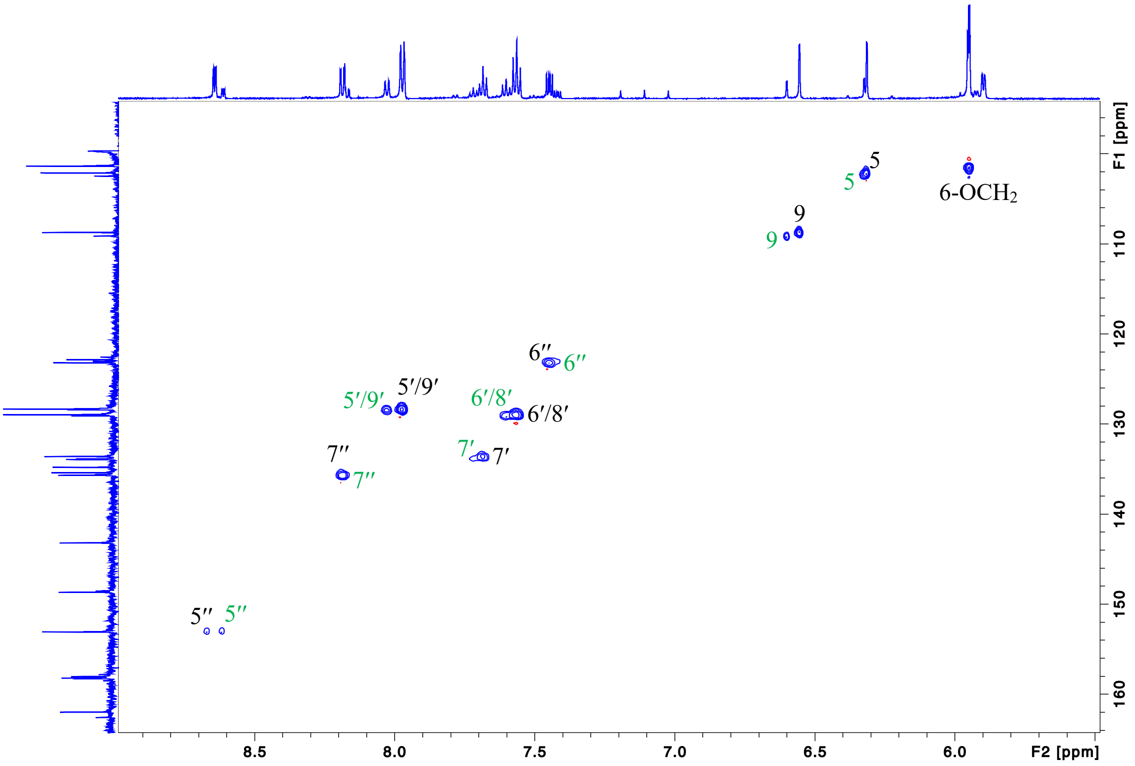

Figure S16. Expanded HSQC NMR (600 MHz, DMSO-d_6) spectrum (part 2) for azachrysosporazine B1 (**3**), major rotamer (labelled black); minor rotamer (labelled green).

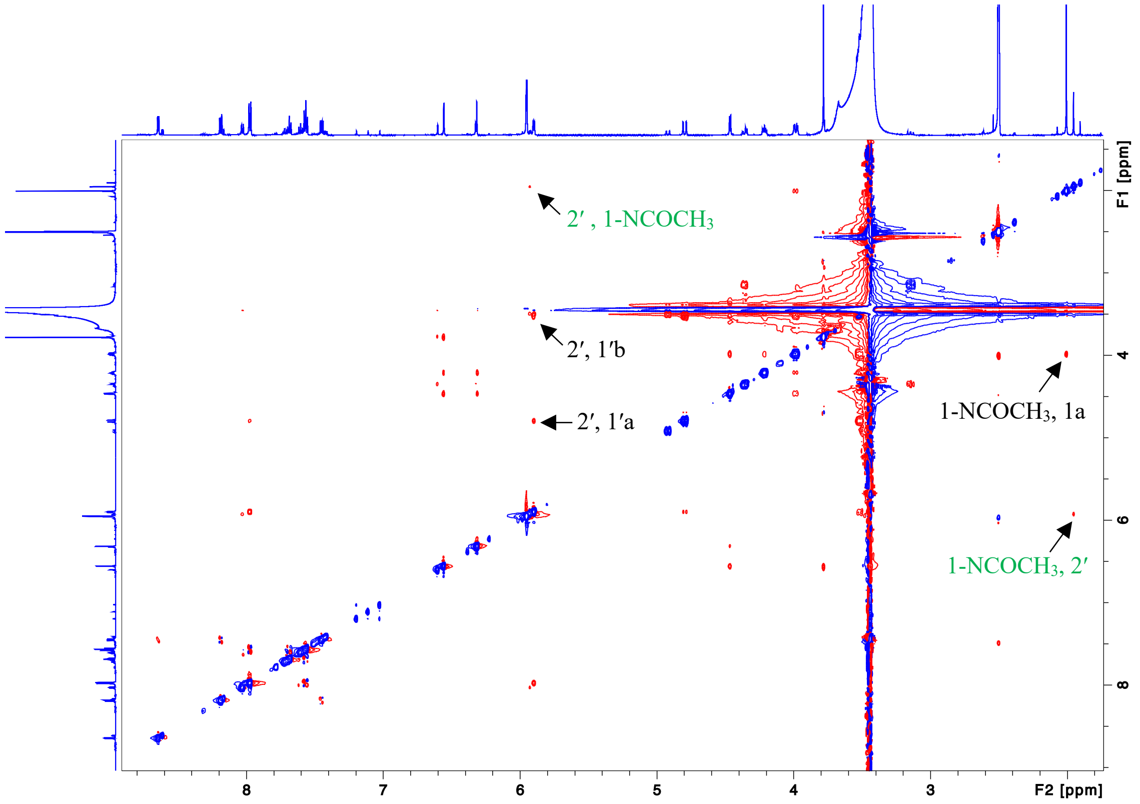

Figure S17. ROESY NMR (600 MHz, DMSO-d_6) spectrum for azachrysosporazine B1 (**3**), major rotamer (labelled black); minor rotamer (labelled green).

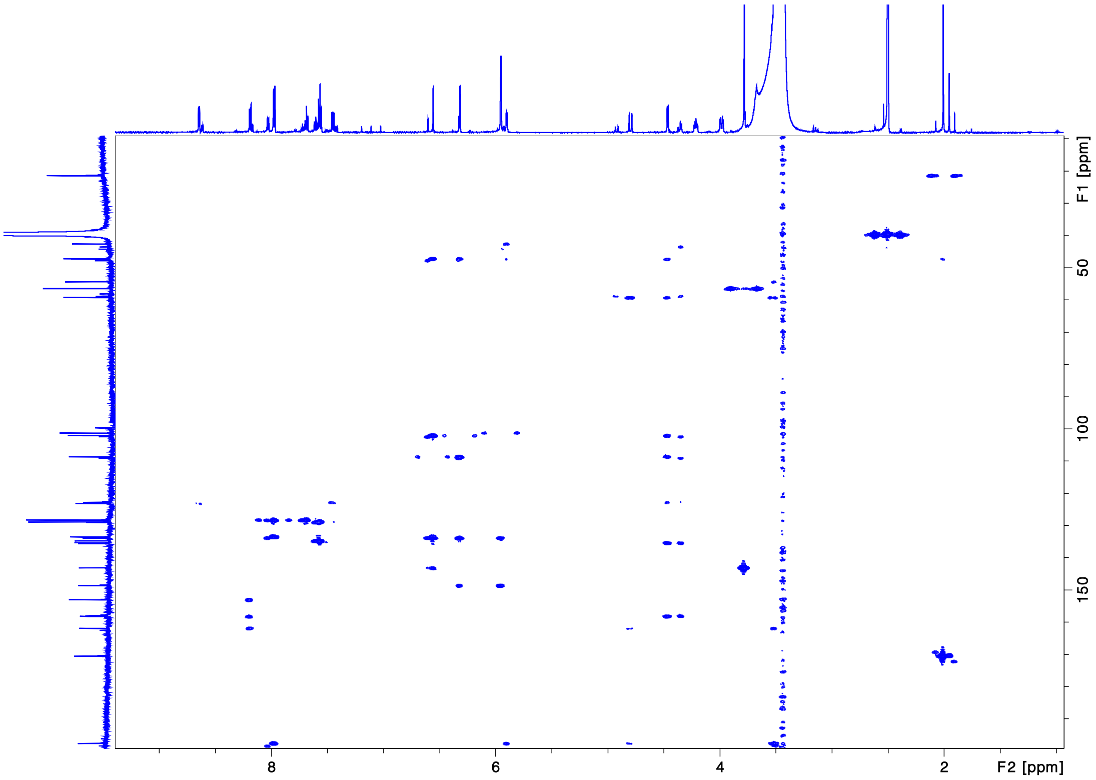

Figure S18. HMBC NMR (600 MHz, DMSO-d_6) spectrum for azachrysosporazine B1 (**3**)

6.4 Azachrysosporazine C1 (4)

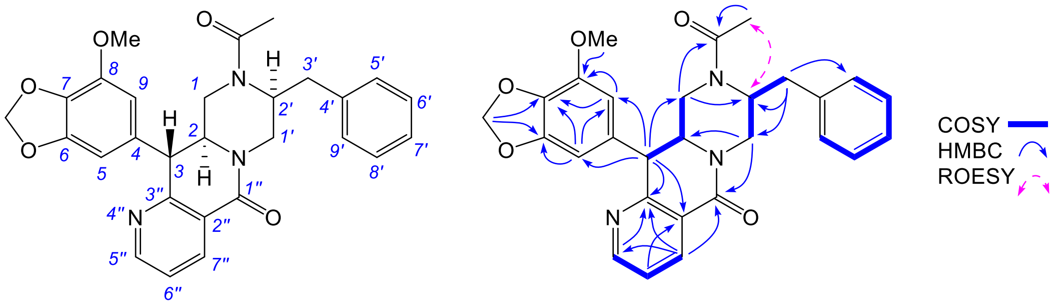

Table S4. 1D and 2D NMR (600 MHz, DMSO-d_6) data for azachrysosporazine C1 (**4**) (major rotamer)

**Position**	δ_H, mult (*J* in Hz)	δ_C	COSY	^{1}H-^{13}C HMBC	ROSEY
1	a 4.29, dd (*13.8, 3.8*)	40.1[b]	1b, 2	2′, 1-NCO	1b, 2
	b 3.04[a], dd (*13.8, 11.4*)		1a, 2	2′	1a, 3
2	3.88, ddd (*11.4, 10.0, 3.8*)	58.0	1, 3	ND	1a, 1b, 5, 9
3	4.49, d (*10.0*)	48.8	2	1, 2, 4, 5, 9, 2″, 3″	1b, 5, 9
4	---	134.6	---	---	---
5	6.49, d (*1.4*)	103.1	9	3, 6, 7, 9	2, 3
6	---	148.5	---	---	---
7	---	133.8	---	---	---
8	---	143.1	---	---	---
9	6.63, d (*1.4*)	109.6	5	3, 5, 7, 8	2, 3, 8-OCH_3
1′	a 4.56, dd (*13.4, 1.1*)	45.0	1′b, 2′	2, 2′, 3′, 1″	1′b, 2′
	b 3.00, m		1′a, 2′	2, 2′, 3′	1′a
2′	4.25, m	54.6	1′b, 3′	ND	1′a, 1′b, 3′a, 1-$NCOCH_3$
3′	a 3.03[a], m	34.9	2′, 3′b	1′, 2′, 4′, 5′/9′	3′b, 5′/9′
	b 2.90, dd (*13.4, 5.8*)		2′, 3′a	1′, 2′, 4′, 5′/9′	3′a, 5′/9′
4′	---	138.2	---	---	---
5′/ 9′	7.26, m	129.4	6′/ 8′	3′, 5′/9′, 7′	2′, 3′a, 3′b
6′/ 8′	7.30, m	128.4	5′/ 9′, 7′	4′, 6′/8′	---
7′	7.22, m	126.5	6′/ 8′	5′/9	---
1″	---	163.5	---	---	---
2″	---	122.9	---	---	---
3″	---	158.4	---	---	---
**N**					
5″	8.60, dd (*4.7, 1.8*)	152.5	6″	3″, 6″, 7″	6″
6″	7.45, dd (*7.8, 4.7*)	122.7	5″, 7″	2″, 5″	5″
7″	8.18, dd (*7.8, 1.8*)	135.5	6″	1″, 3″, 5″	---
1-N<u>CO</u>	---	168.4	---	---	---
1-NCO<u>CH_3</u>	1.60, s	20.7	---	2′, 1-NCO	2′
6-OCH_2	5.99/5.99, AB_q	101.2	---	6, 7	---
8-OCH_3	3.80, s	56.4	---	8	9

(a) overlapping signals within same letter, (b) obscured by solvent signal

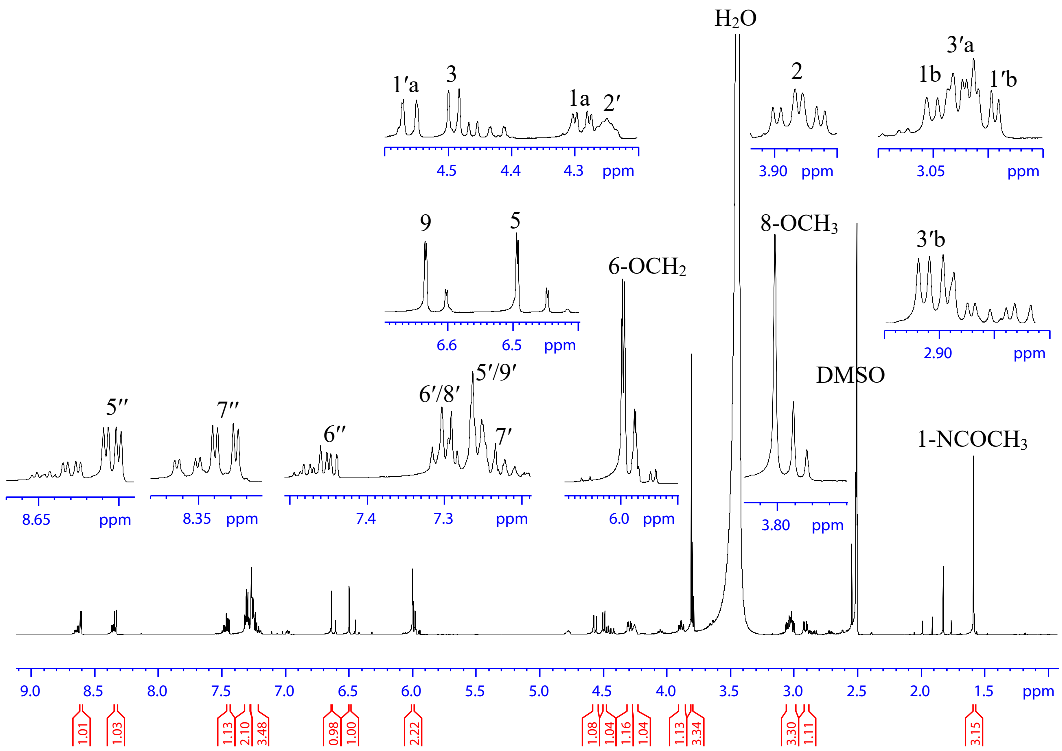

Figure S19. ^{1}H NMR (600 MHz, DMSO-d_6) spectrum for azachrysosporazine C1 (**4**) (major rotamer)

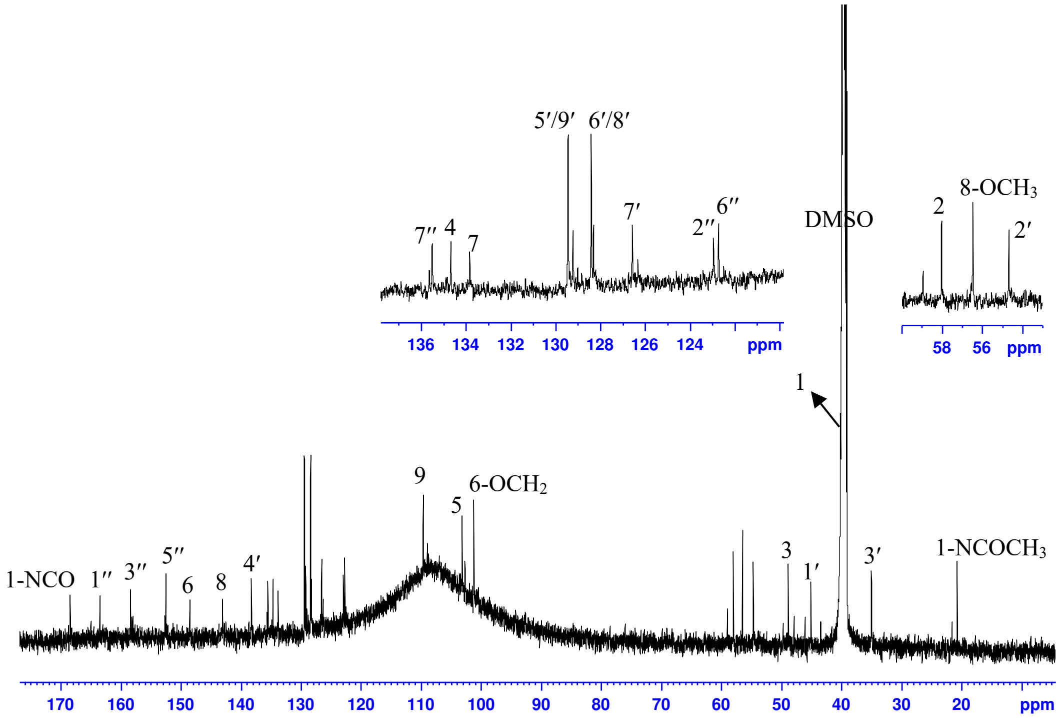

Figure S20. ^{13}C NMR (150 MHz, DMSO-d_6) spectrum for azachrysosporazine C1 (**4**) (major rotamer).

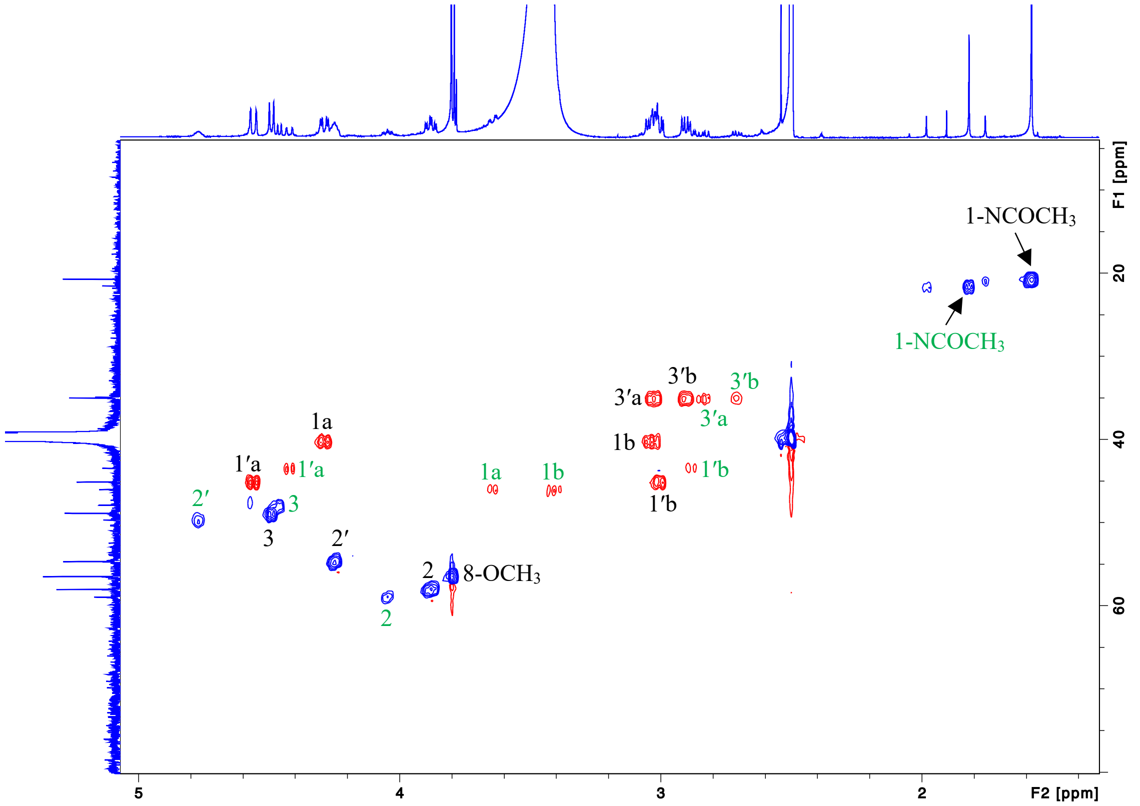

Figure S21. Expanded HSQC NMR (600 MHz, DMSO-d_6) spectrum (part 1) for azachrysosporazine C1 (**4**), major rotamer (labelled black); minor rotamer (labelled green).

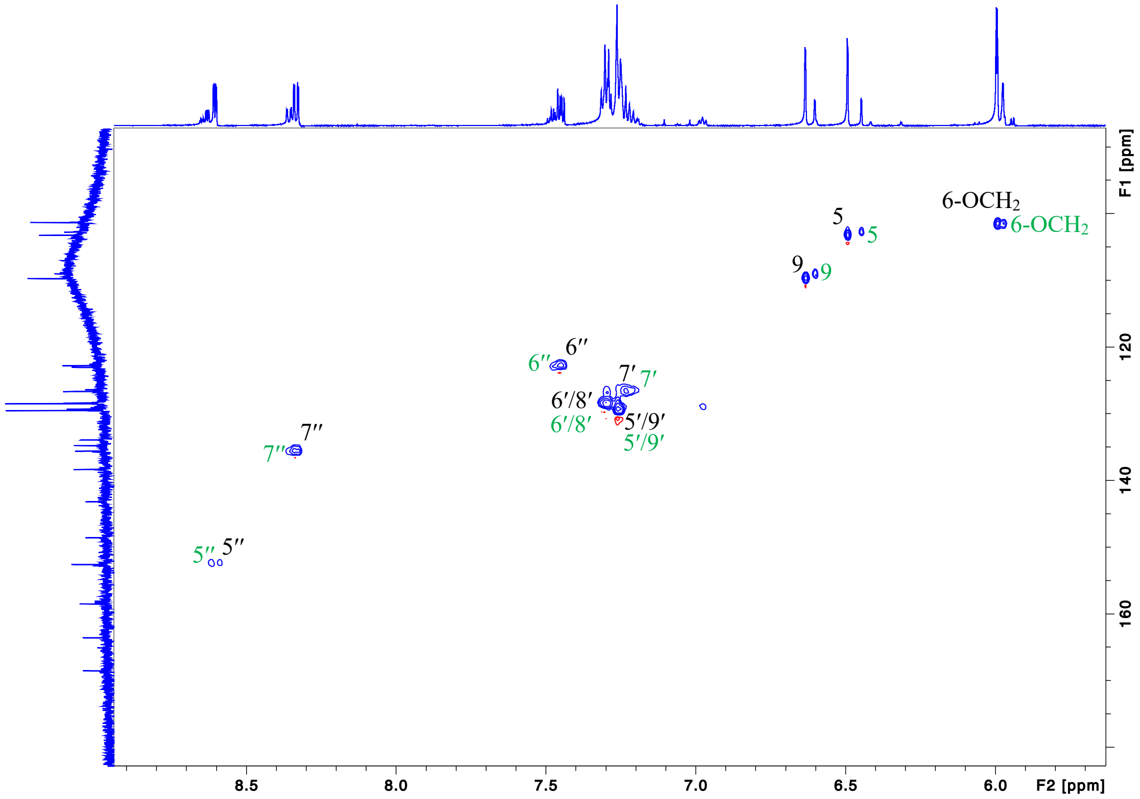

Figure S22. Expanded HSQC NMR (600 MHz, DMSO-d_6) spectrum (part 2) for azachrysosporazine C1 (**4**), major rotamer (labelled black); minor rotamer (labelled green).

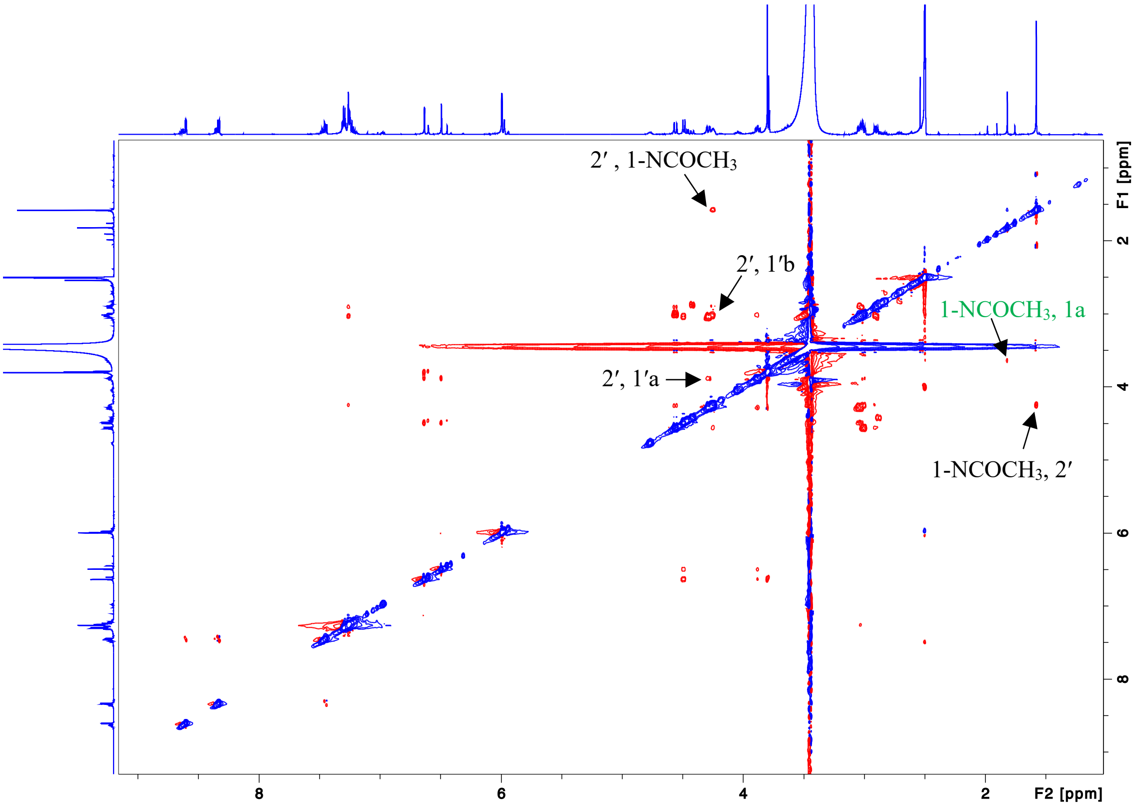

Figure S23. ROESY NMR (600 MHz, DMSO-d_6) spectrum for azachrysosporazine C1 (**4**), major rotamer (labelled black); minor rotamer (labelled green).

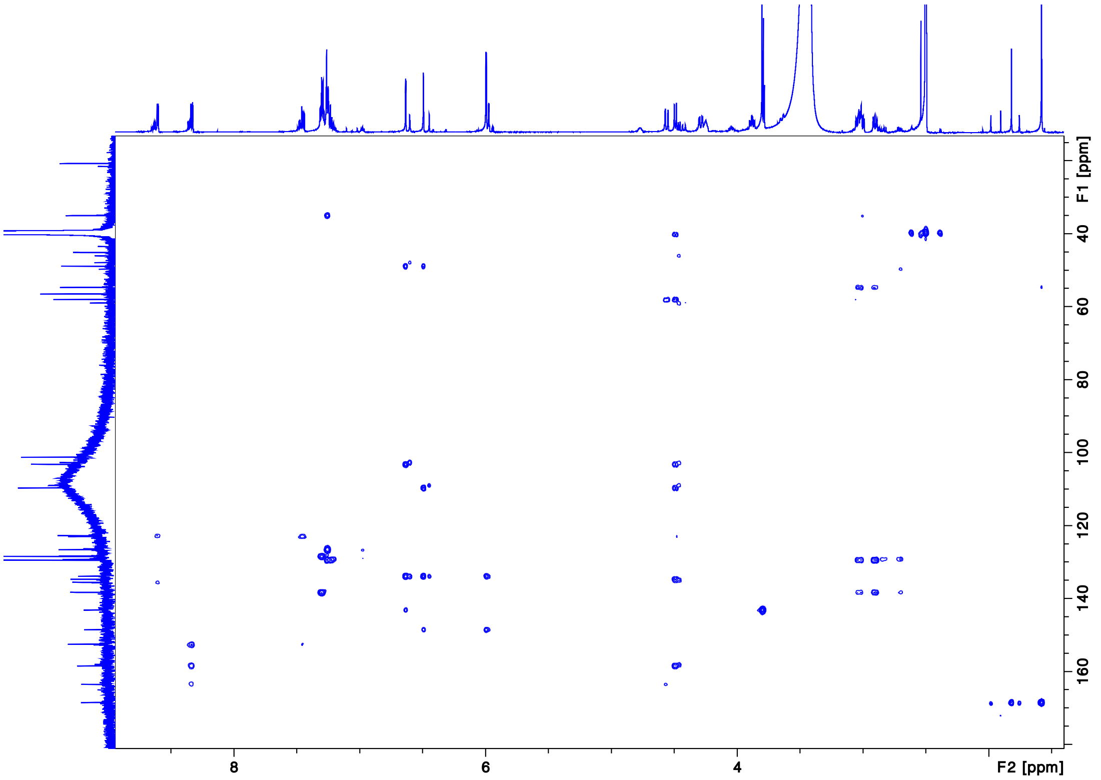

Figure S24. HMBC NMR (600 MHz, DMSO-d_6) spectrum for azachrysosporazine C1 (**4**)

6.5 Azachrysosporazine C2 (5)

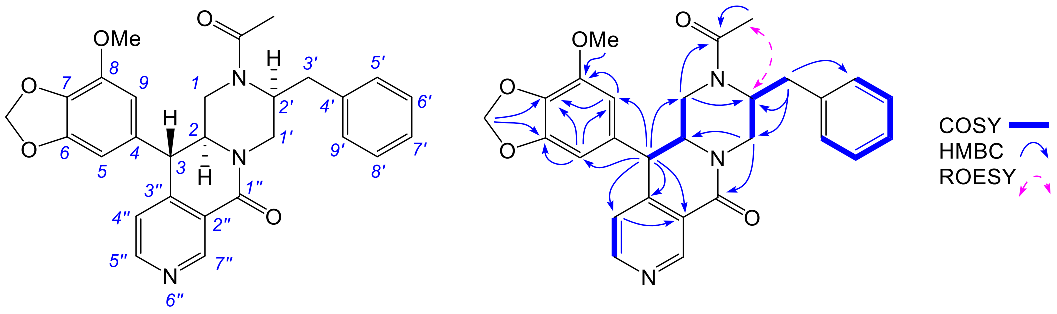

Table S5. 1D and 2D NMR (600 MHz, DMSO-d_6) data for azachrysosporazine C2 (**5**) (major rotamer)

Position	δ_H, mult (*J* in Hz)	δ_C	COSY	^{1}H-^{13}C HMBC	ROESY
1	a 4.19, dd (*13.8, 3.9*)	39.8[a]	1b, 2	2′	1b, 2
	b 2.99, dd (*13.8, 11.0*)		1a, 2	2, 3	1a, 3
2	3.91, ddd (*11.2, 11.0, 3.9*)	56.9	1, 3	---	1a, 1′b, 5, 9
3	4.46, d (*11.2*)	45.7	2	1, 2, 4, 5, 9, 2″, 3″, 4″	1b, 5, 9, 4″
4	---	132.6	---	---	---
5	6.64, d (*1.3*)	103.0	9	3, 6, 7, 9	2, 3
6	---	148.9	---	---	---
7	---	134.4	---	---	---
8	---	143.5	---	---	---
9	6.73, d (*1.3*)	109.5	5	3, 5, 7, 8	2, 3, 8-OCH$_3$
1′	a 4.56, dd (*13.5, 1.2*)	44.5	1′b, 2′	2, 1″	1′b, 2′
	b 2.95, dd (*13.5, 4.1*)		1′a, 2′	3′	1′a, 2′
2′	4.27, m	54.3	1′b, 3′	---	1′a, 1′b, 5′/9′, 1-NCOCH$_3$
3′	a 3.06, dd (*13.4, 8.8*)	34.8	2′, 3′b	1′, 2′, 4′, 5′/9′	3′b, 5′/9′
	b 2.93, dd (*13.4, 5.8*)		2′, 3′a	4′, 5′/9′	3′a, 5′/9′
4′	---	138.2	---	---	---
5′/9′	7.26, m	129.4	6′/8′	3′, 5′/9′, 7′	1′a, 2′, 3′a, 3′b
6′/8′	7.30, m	128.4	5′/9′, 7′	4′, 6′/8′	---
7′	7.23, m	126.5	6′/8′	5′/9′	---
1″	---	162.9	---	---	---
2″	---	123.0	---	---	---
3″	---	149.3	---	---	---
4″	6.72, br s	121.5	5″	3, 2″	---
5″	8.60, br s	152.4	4″	**ND**	---
N					
7″	9.08, br s	148.3	---	**ND**	---
1-N<u>CO</u>	---	168.3	---	---	---
1-NCO<u>CH$_3$</u>	1.59, s	20.6	---	2′, 1-NCO	2′
6-OCH$_2$	6.05, br s	101.5	---	6, 7	---
8-OCH$_3$	3.82, s	56.4	---	8	9

(a) obscured by solvent signal

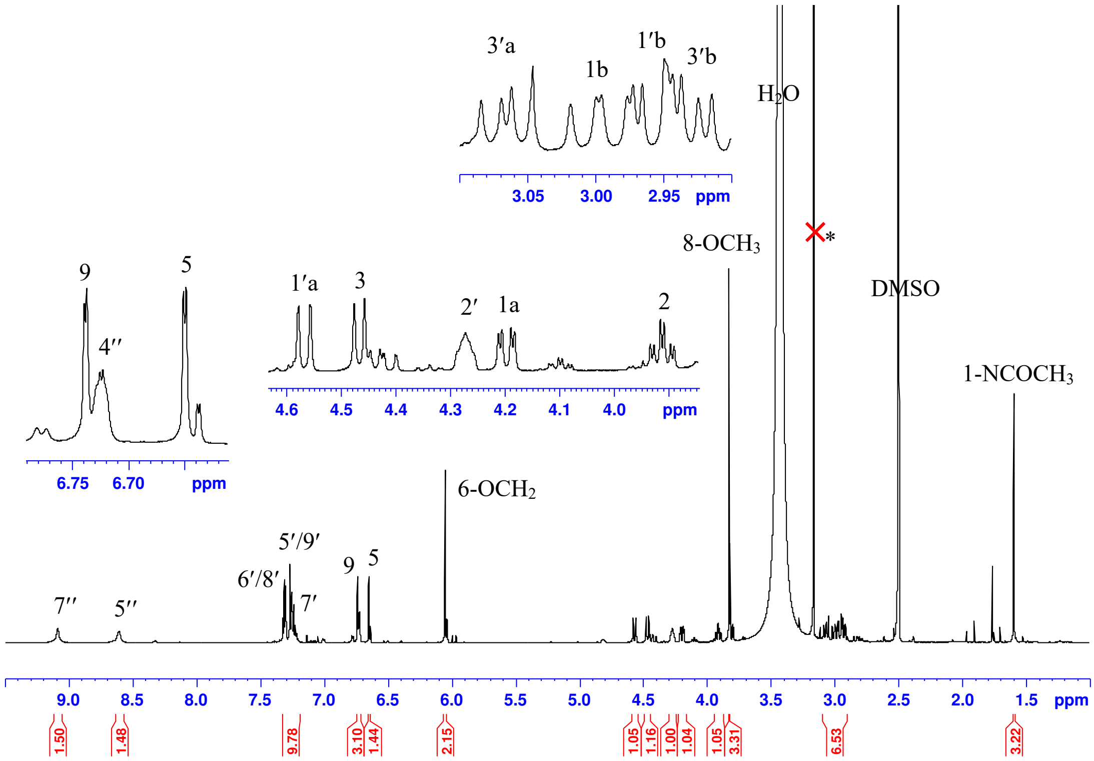

Figure S25. ^{1}H NMR (600 MHz, DMSO-d_6) spectrum for azachrysosporazine C2 (**5**) (major rotamer). * residual MeOH solvent.

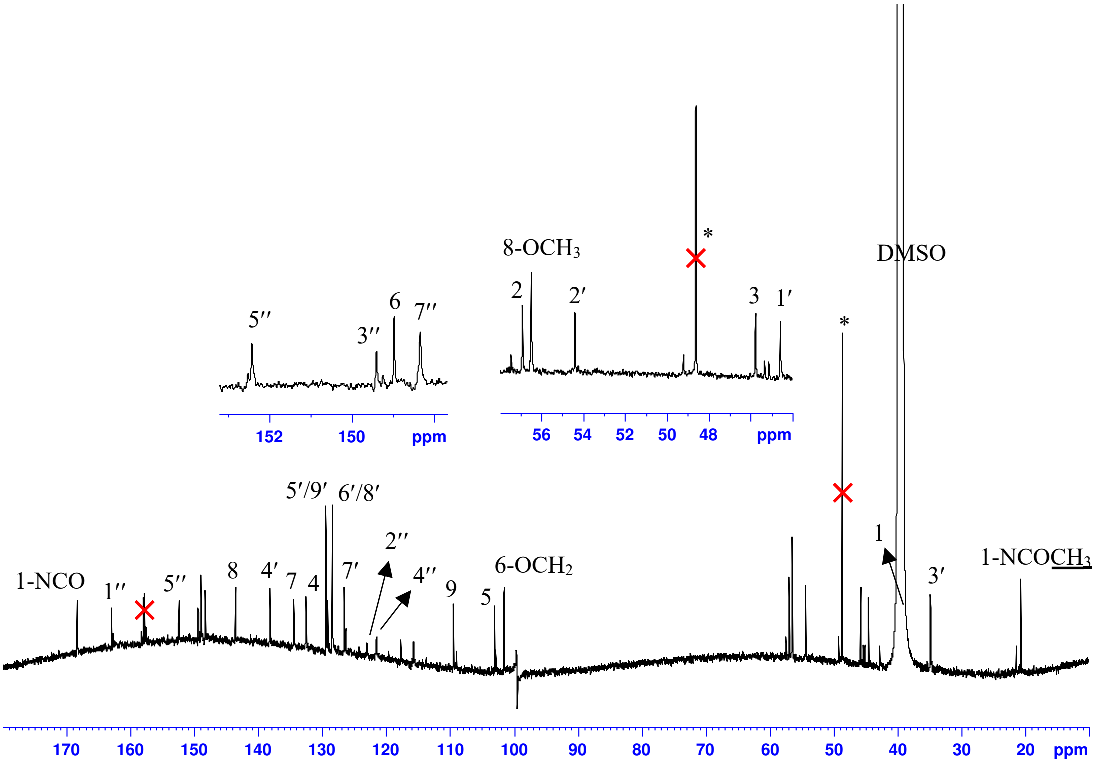

Figure S26. ^{13}C NMR (150 MHz, DMSO-d_6) spectrum for azachrysosporazine C2 (**5**) (major rotamer). * residual MeOH solvent.

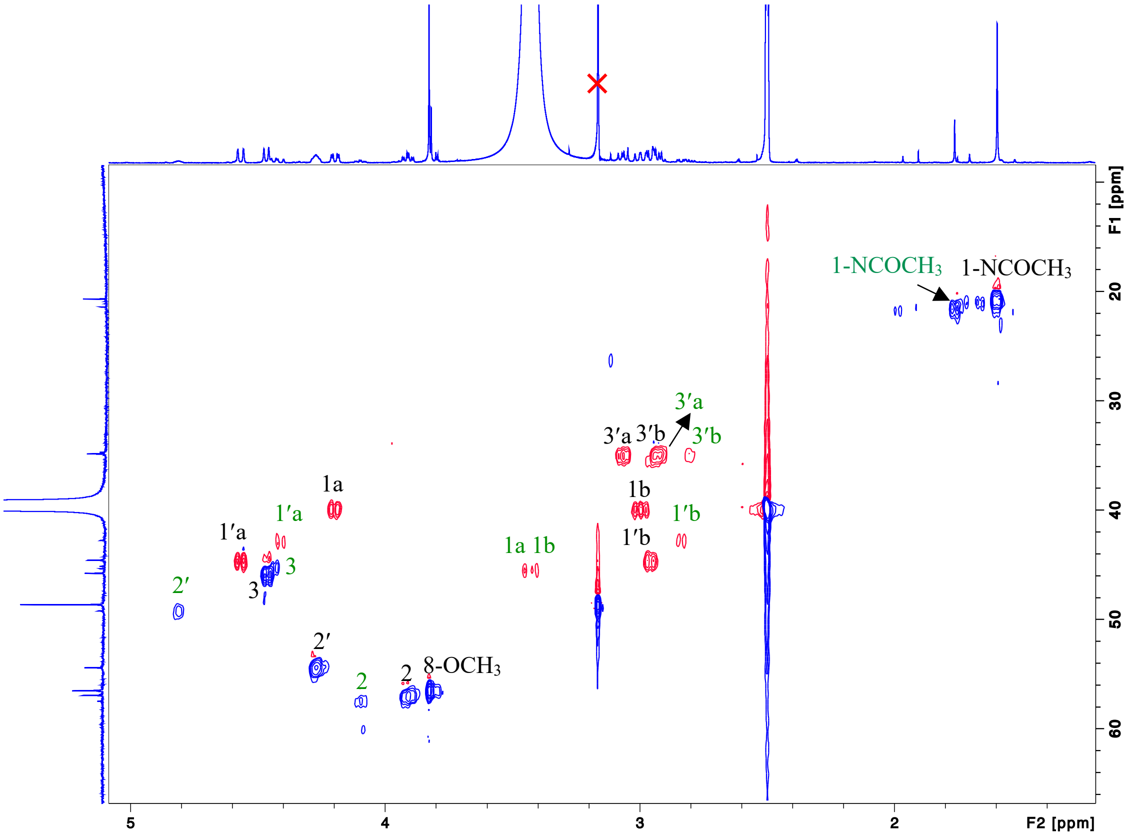

Figure S27. Expanded HSQC NMR (600 MHz, DMSO-d_6) spectrum (part 1) for azachrysosporazine C2 (**5**), major rotamer (labelled black); minor rotamer (labelled green).

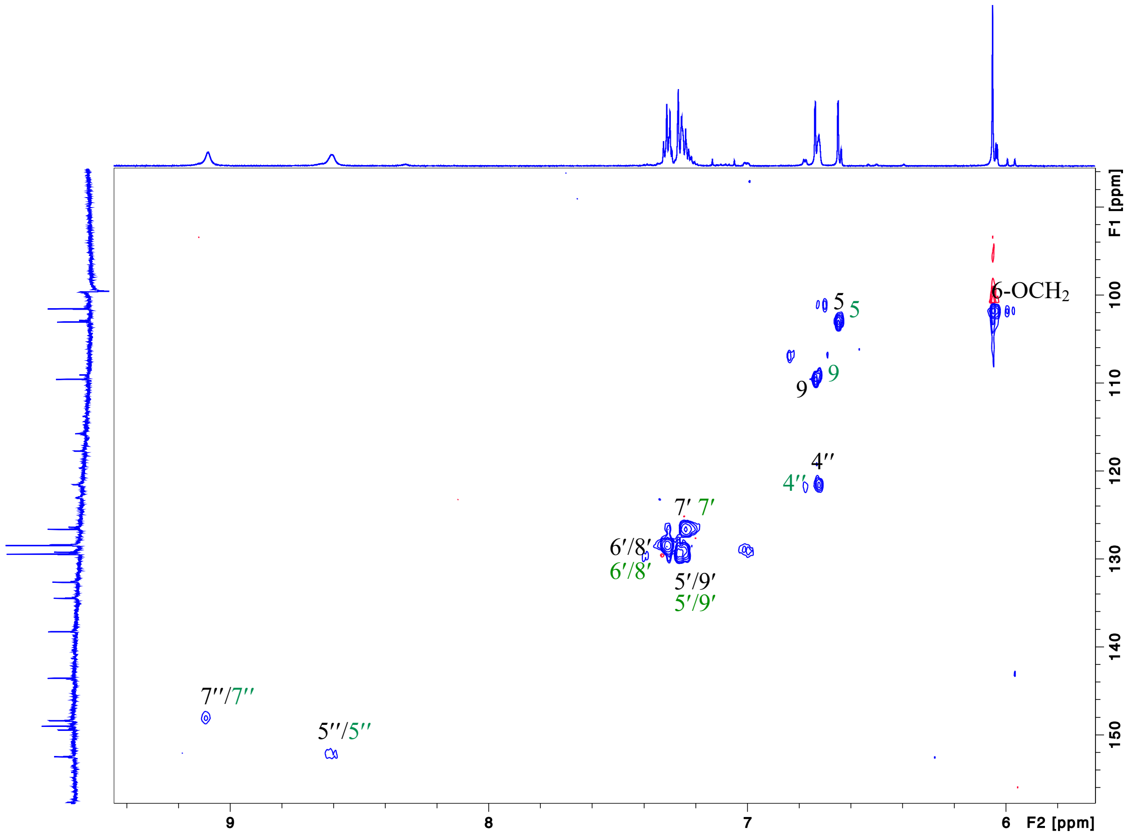

Figure S28. Expanded HSQC NMR (600 MHz, DMSO-d_6) spectrum (part 2) for azachrysosporazine C2 (**5**), major rotamer (labelled black); minor rotamer (labelled green).

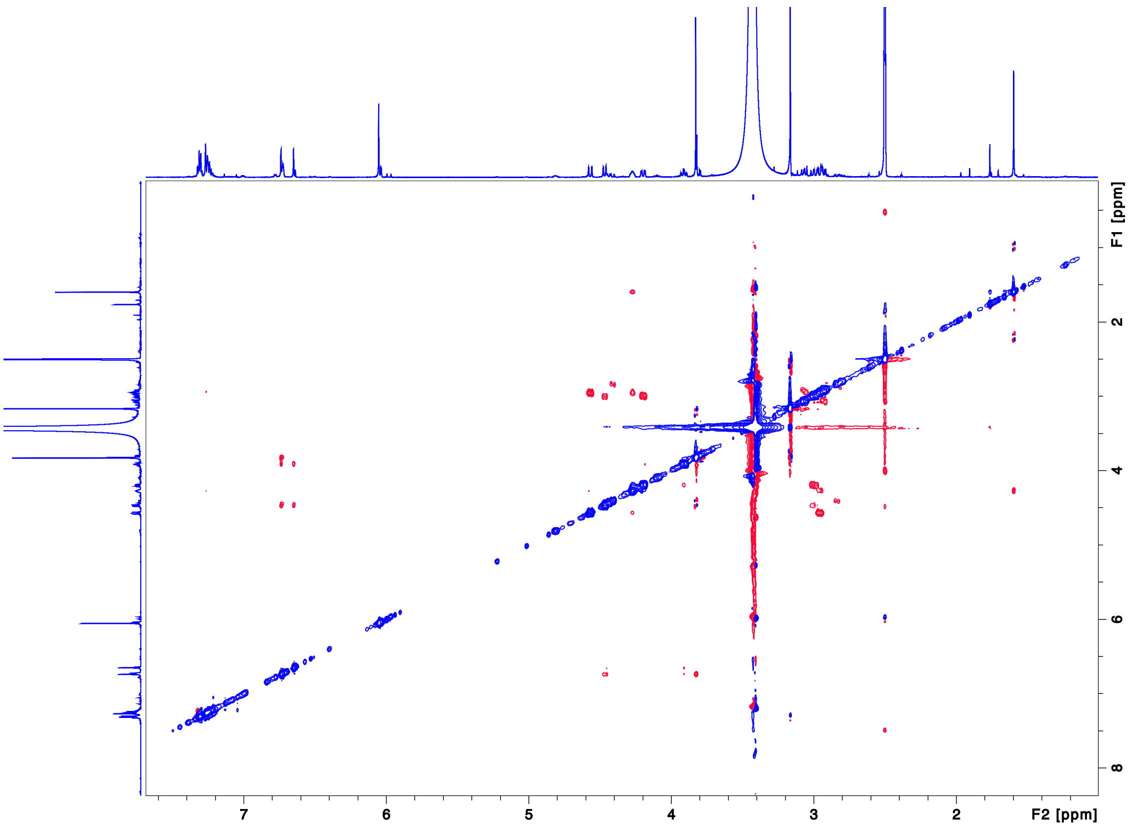

Figure S29. ROESY NMR (600 MHz, DMSO-d_6) spectrum for azachrysosporazine C2 (**5**), major rotamer (labelled black); minor rotamer (labelled green).

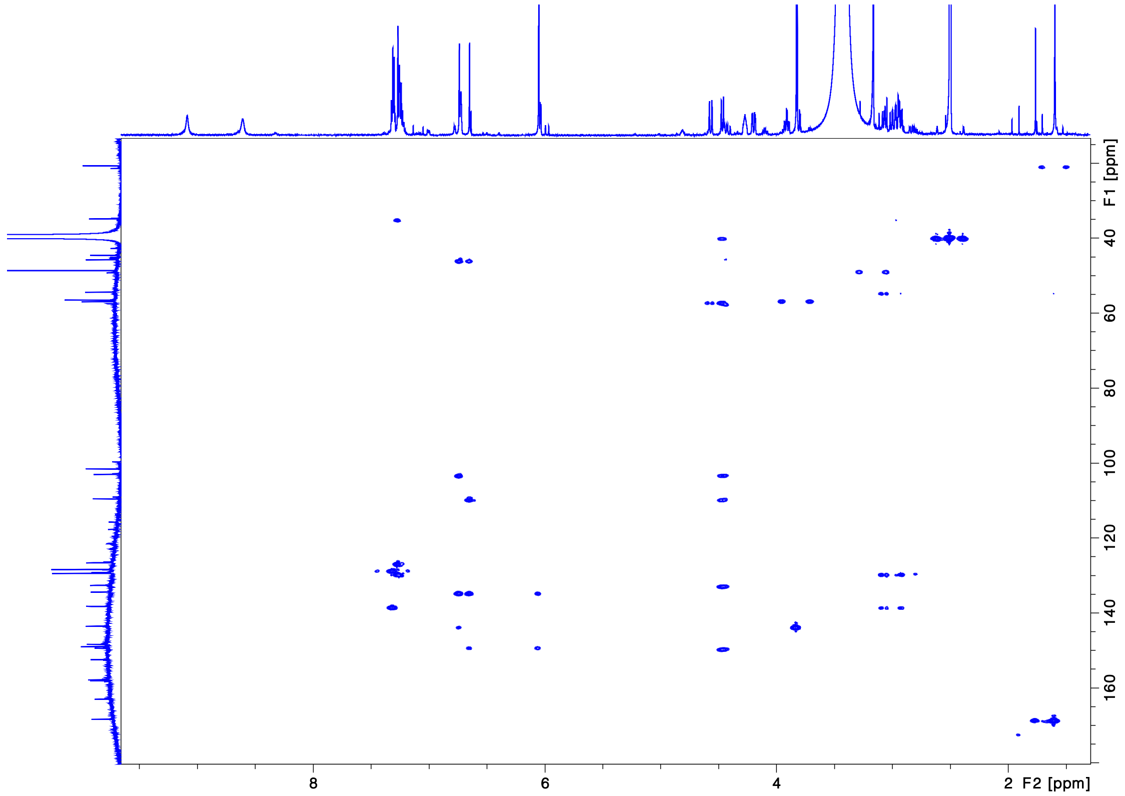

Figure S 30. HMBC NMR (600 MHz, DMSO-d_6) spectrum for azachrysosporazine C2 (**5**)

6.6 Azachrysosporazine D1 (6)

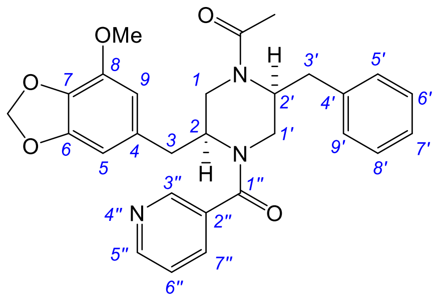

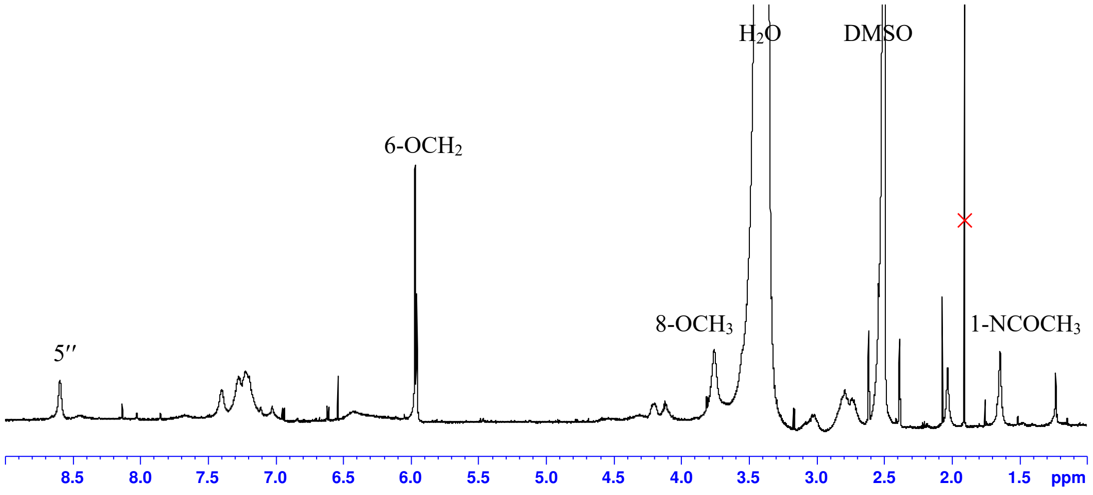

Figure S31. ^{1}H NMR (600 MHz, DMSO-d_6) spectrum for azachrysosporazine D1 (**6**).

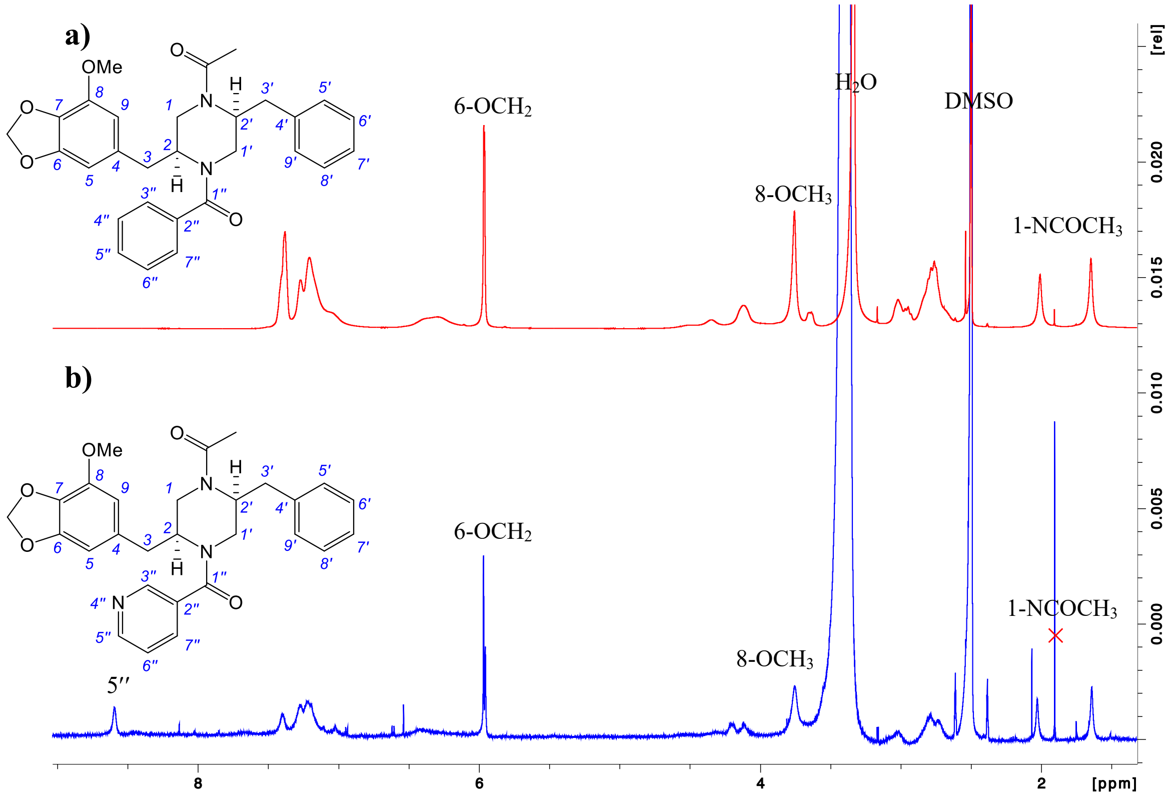

Figure S32. ^{1}H NMR (600 MHz, DMSO-d_6) spectra for a) chrysosporazine D and b) azachrysosporazine D1 (**6**)

6.7 chrysosporazine N (7)

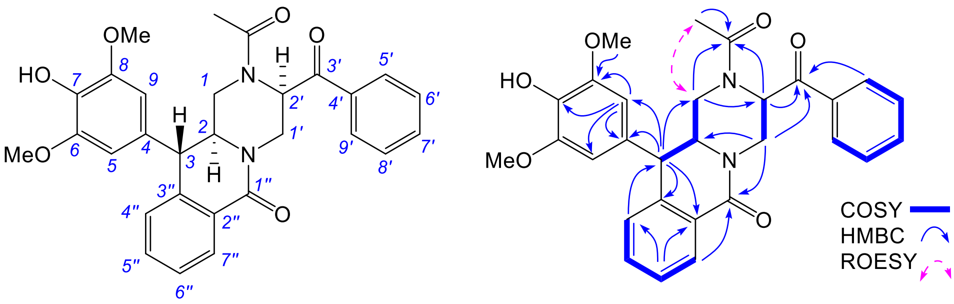

Table S6. 1D and 2D NMR (600 MHz, DMSO-d_6) data for chrysosporazine N (**7**) (major rotamer)

Position	δ_H, mult (*J* in Hz)	δ_C	COSY	^{1}H-^{13}C HMBC	ROESY
1	a 3.77, dd (*13.6, 4.1*)	46.0	1b, 2	2′	1-NCOCH$_3$
	b 3.43[a]		1a, 2	2, 3, 1-NCO	
2	4.27, ddd (*12.4, 9.0, 4.1*)	58.5	1a, 1b, 3		1b, 1′b, 5/9
3	4.36, d (*9.0*)	45.4	2	1, 2, 4, 5/9, 2″, 3″	1a, 1b, 5/9
4	---	130.1	---	---	---
5/9	6.54, s	106.3	---	3, 4, 5/9, 6/8, 7	2, 3, 6/8-OCH$_3$, 1-NCOCH$_3$
6/8	---	148.3	---	---	---
7	---	134.7[b]	---	---	---
1′	a 4.76, dd (*14.3, 1.9*)	40.9	1′b, 2′	2, 3′, 1″	1′b, 2′, 5′/9′
	b 3.52, dd (*14.3, 5.5*)		1′a, 2′	2′, 3′, 1″	1′a, 2′
2′	5.83, dd (*5.5, 1.9*)	55.1	1′a, 1′b	1, 1′, 3′, 1-NCO	1′a, 1′b, 5′/9′
3′	---	196.6	---	---	---
4′	---	134.7[b]	---	---	---
5′/9′	7.97, dd (*8.1, 1.0*)	128.2	6′/8′	3′, 5′/9′, 7′	1′a, 2′
6′/8′	7.55, dd (*8.1, 7.6*)	128.9	5′/9′, 7′	4′, 6′/8′	---
7′	7.67, t (*7.6*)	133.5	6′/8′	5′/9′	---
1″	---	162.8	---	---	---
2″	---	127.4	---	---	---
3″	---	140.9	---	---	---
4″	6.82, d (*8.7*)	127.7	5″	3, 2″, 6″	3, 5/9
5″	7.45, ddd (*8.7, 7.4, 1.5*)	132.4	4″, 6″	3″, 7″	4″
6″	7.35, dd (*7.7, 7.4*)	127.0	5″, 7″	2″/7″, 4″	---
7″	7.85, dd (*7.7, 1.5*)	127.5	6″	1″, 3″, 5″	---
1-NCO	---	169.9	---	---	---
1-NCOCH$_3$	1.91, s	21.2	---	1, 1-NCO	1a
6/8-OCH$_3$	3.69, s	56.1	---	6/8	5/9
7-OH	8.43, br s	---	---	6/8	---

(a) obscured by solvent signal, (b) signals are interchangeable within the same letter

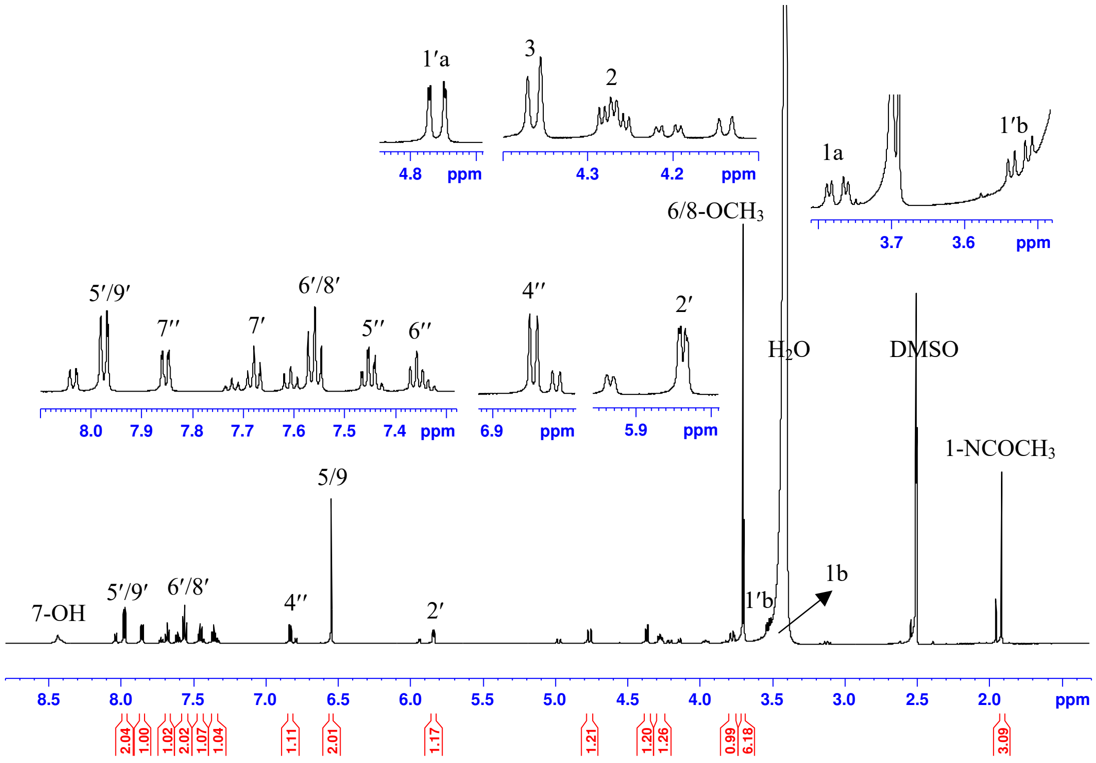

Figure S33. ^{1}H NMR (600 MHz, DMSO-d_6) spectrum for chrysosporazine N (**7**) (major rotamer).

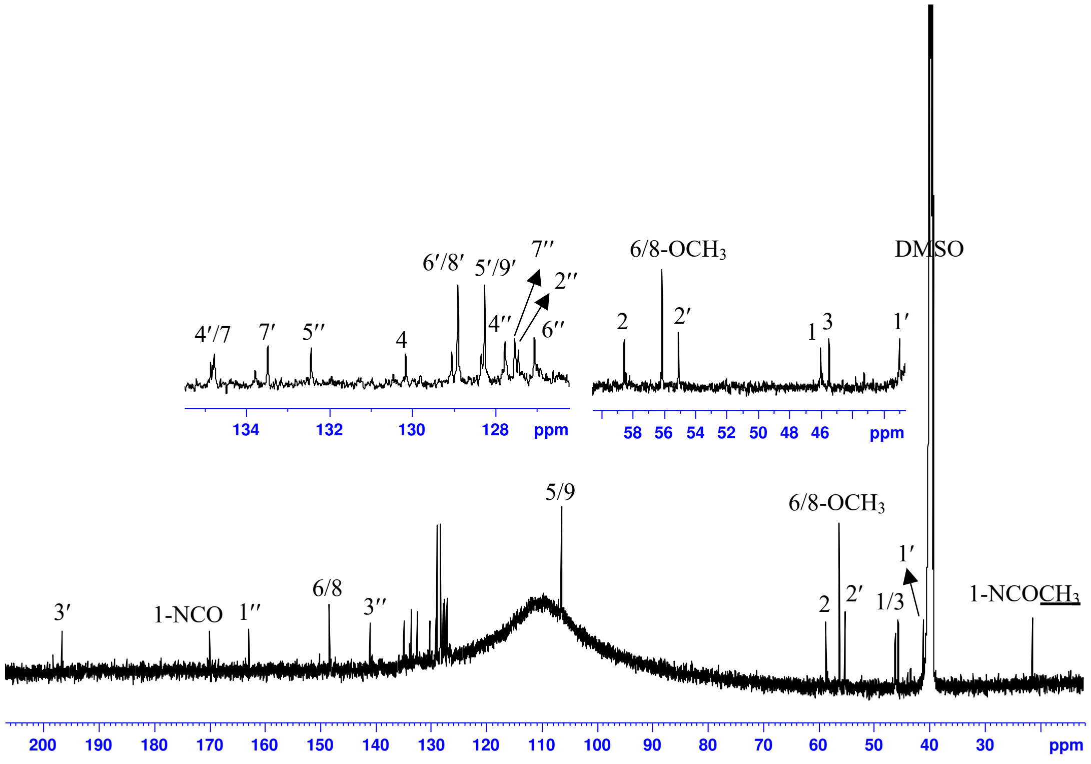

Figure S34. ^{13}C NMR (150 MHz, DMSO-d_6) spectrum for chrysosporazine N (**7**) (major rotamer).

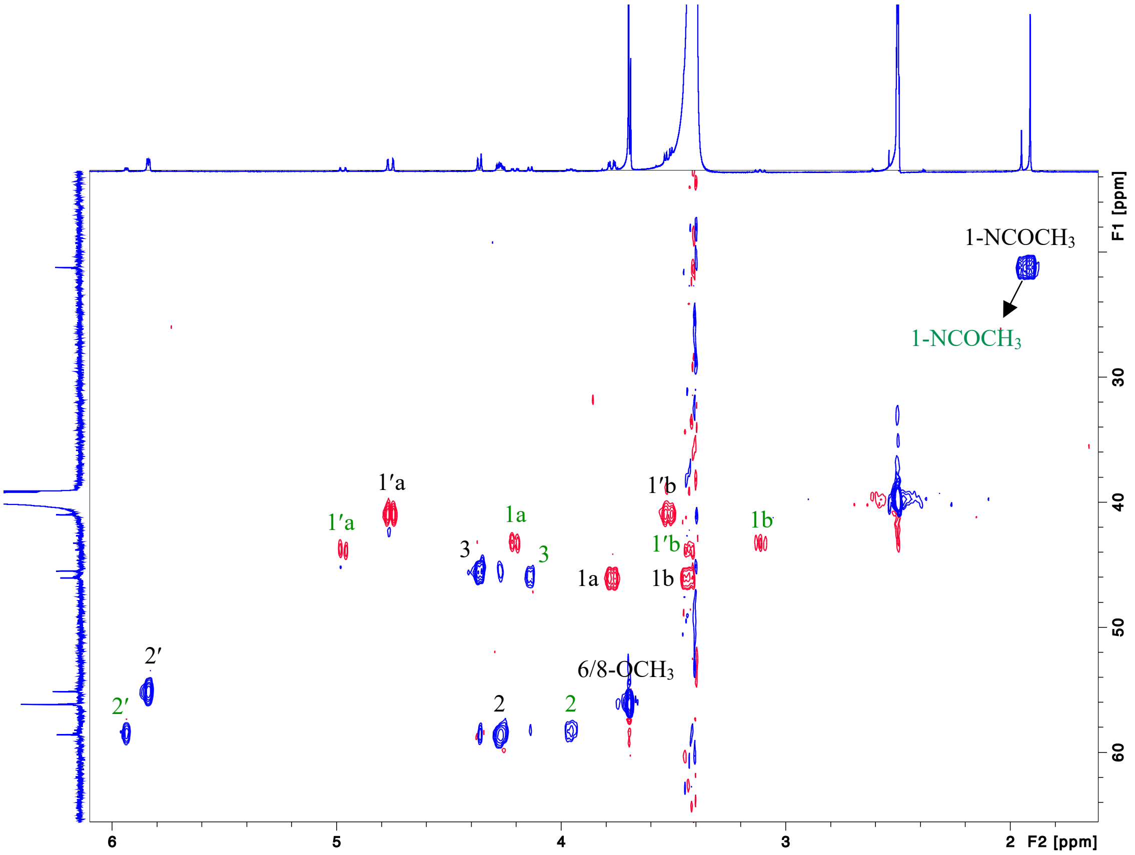

Figure S35. Expanded HSQC NMR (600 MHz, DMSO-d_6) spectrum (part 1) for chrysosporazine N (**7**), major rotamer (labelled black); minor rotamer (labelled green).

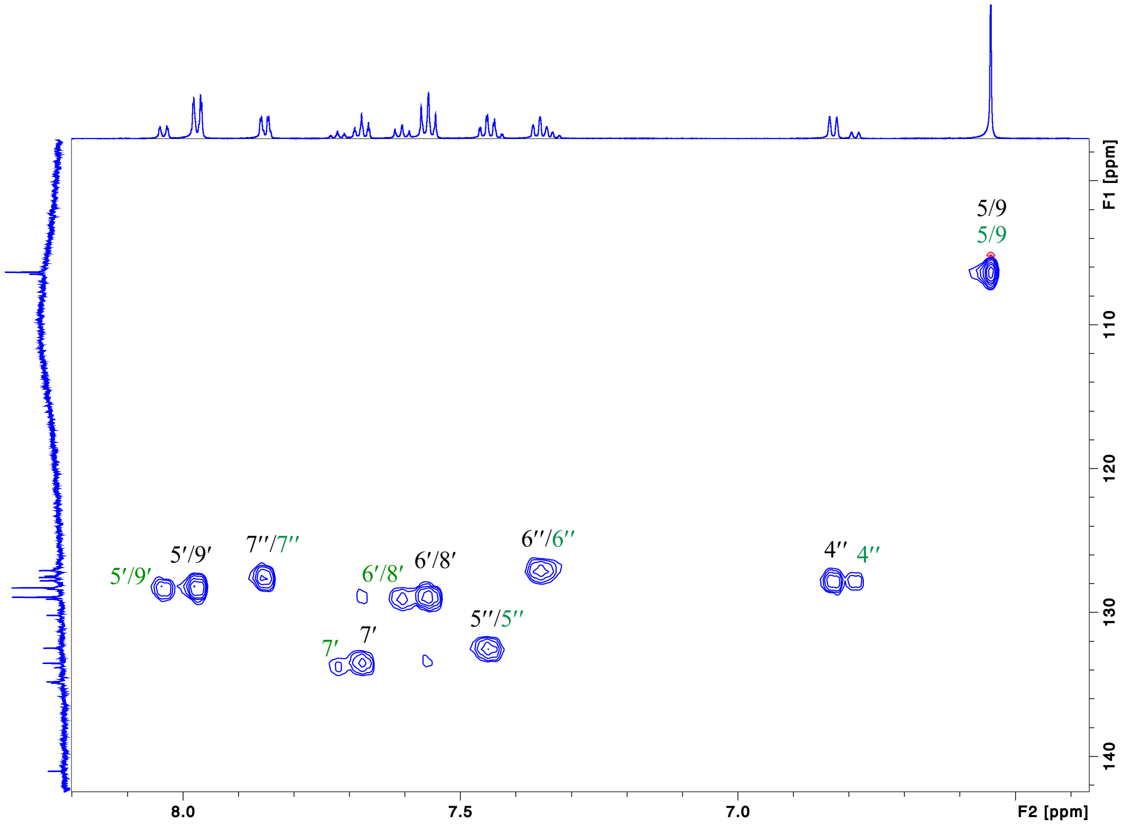

Figure S36. Expanded HSQC NMR (600 MHz, DMSO-d_6) spectrum (part 2) for chrysosporazine N (**7**), major rotamer (labelled black); minor rotamer (labelled green).

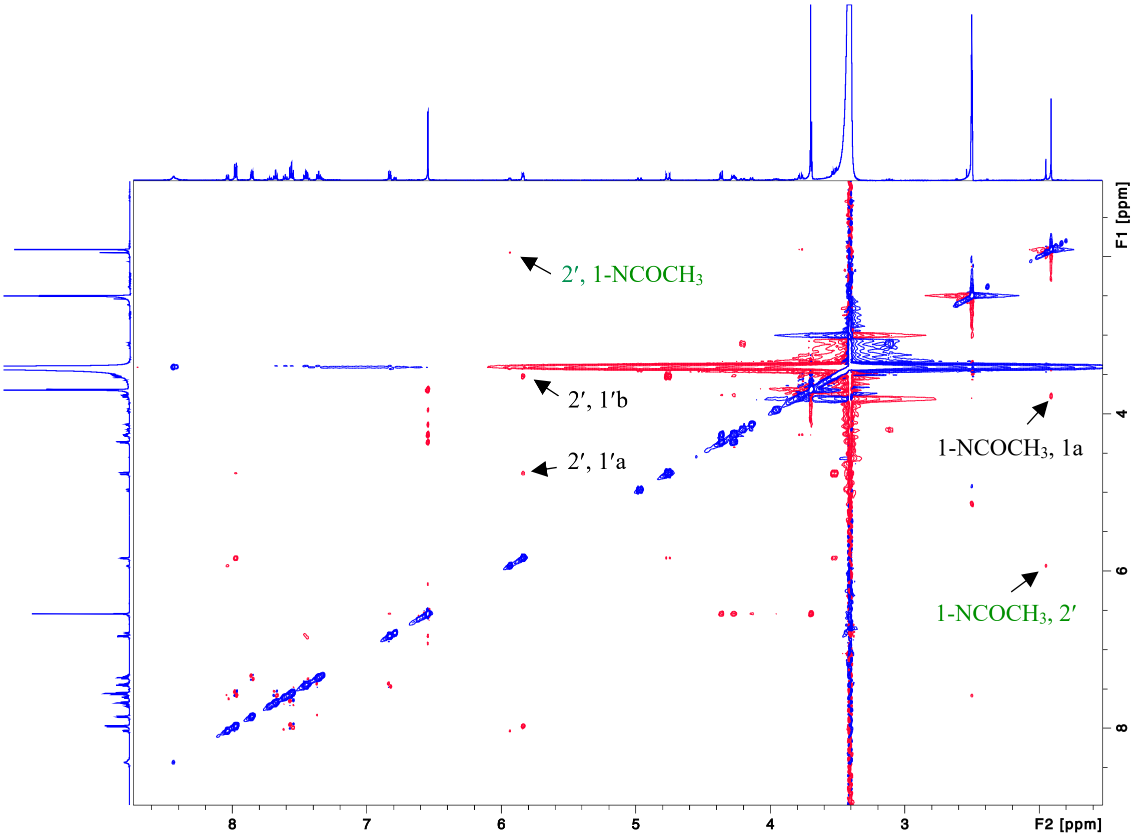

Figure S37. ROESY NMR (600 MHz, DMSO-d_6) spectrum for chrysosporazine N (**7**), major rotamer (labelled black); minor rotamer (labelled green).

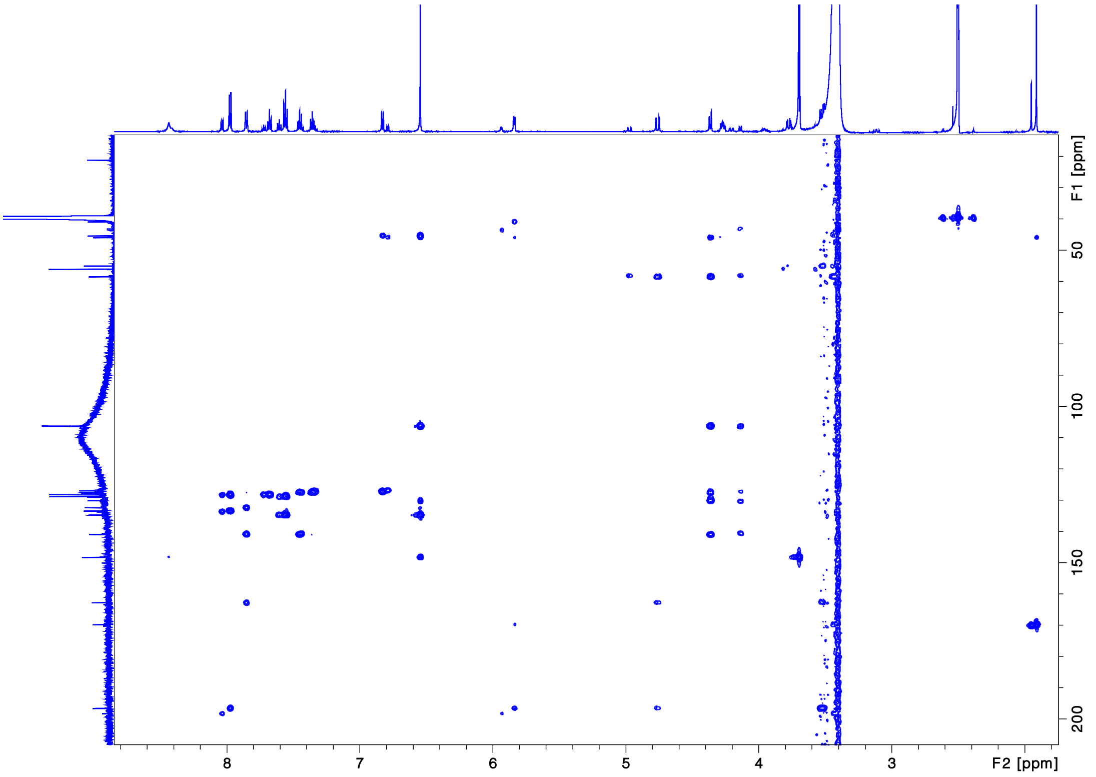

Figure S38. HMBC NMR (600 MHz, DMSO-d_6) spectrum for chrysosporazine N (**7**)

6.8 Chrysosporazine O (8)

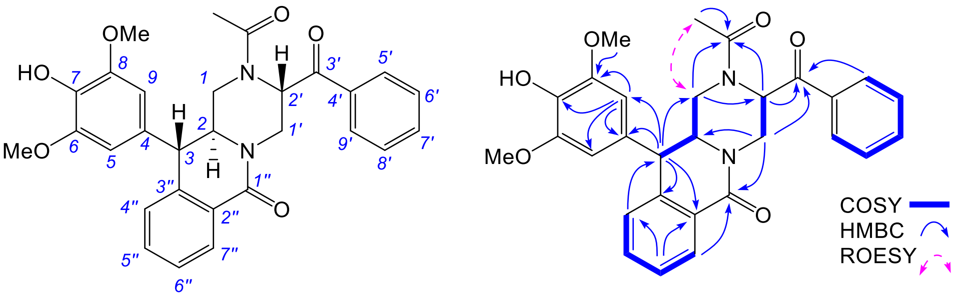

Table S7. 1D and 2D NMR (600 MHz, DMSO-d_6) data for chrysosporazine O (**8**) (major rotamer)

Position	δ_H, mult (*J* in Hz)	δ_C	COSY	^{1}H-^{13}C HMBC	ROESY
1	a 3.68, dd (*14.3, 4.1*)	44.6	1b, 2	2, 3, 2′, 1-NCO	1-NCOCH$_3$
	b 3.61, dd (*14.3, 6.8*)		1a, 2	2, 3, 2′	1-NCOCH$_3$
2	4.47, ddd (*12.4, 6.8, 4.1*)	57.7	1a, 1b, 3	4	1b, 1′b, 5/9
3	4.25, d (*12.4*)	46.9	2	1, 2, 4, 5/9, 2″, 3″	1a, 1b, 5/9
4	---	128.0	---	---	---
5/9	6.61, s	106.6	---	3, 4, 5/9, 6/8, 7	2, 3, 6/8-OCH$_3$
6/8	---	148.5	---	---	---
7	---	135.0	---	---	---
1′	a 4.37, dd (*14.1, 7.1*)	38.8	1′b, 2′	2, 2′, 3′, 1″	1′b
	b 3.98, dd (*14.1, 4.5*)		1′a, 2′	2, 2′, 3′, 1″	1′a, 2′
2′	5.67, dd (*6.1, 5.9*)	57.1	1′a, 1′b	1′, 3′, 1-NCO	1′b, 5′/9′
3′	---	196.3	---	---	---
4′	---	134.6	---	---	---
5′/9′	8.03, dd (*8.4, 1.3*)	128.2	6′/8′	3′, 5′/9′, 7′	2′
6′/8′	7.58, dd (*7.8, 7.6*)	129.1	5′/9′, 7′	4′, 6′/8′	---
7′	7.70, t (*7.8*)	133.8	6′/8′	5′/9′	---
1″	---	162.5	---	---	---
2″	---	127.5^a	---	---	---
3″	---	142.0	---	---	---
4″	6.65, d (*7.8*)	127.1^b	5″	3, 2″, 6″	5″
5″	7.42, ddd (*7.8, 7.4, 1.3*)	132.3	4″, 6″	3″, 7″	4″
6″	7.35, dd (*7.6, 7.4*)	127.1^b	5″, 7″	2″/7″, 4″	7″
7″	7.85, dd (*7.6, 1.3*)	127.5^a	6″	1″, 3″, 5″	6″
1-N<u>CO</u>	---	169.1	---	---	---
1-NCO<u>CH$_3$</u>	1.83, s	20.9	---	1-NCO	1a, 1b
6/8-OCH$_3$	3.74, s	56.2	---	6/8	5/9
7-OH	8.53, br s	---	---	---	---

(a–b) signals are interchangeable within the same letter

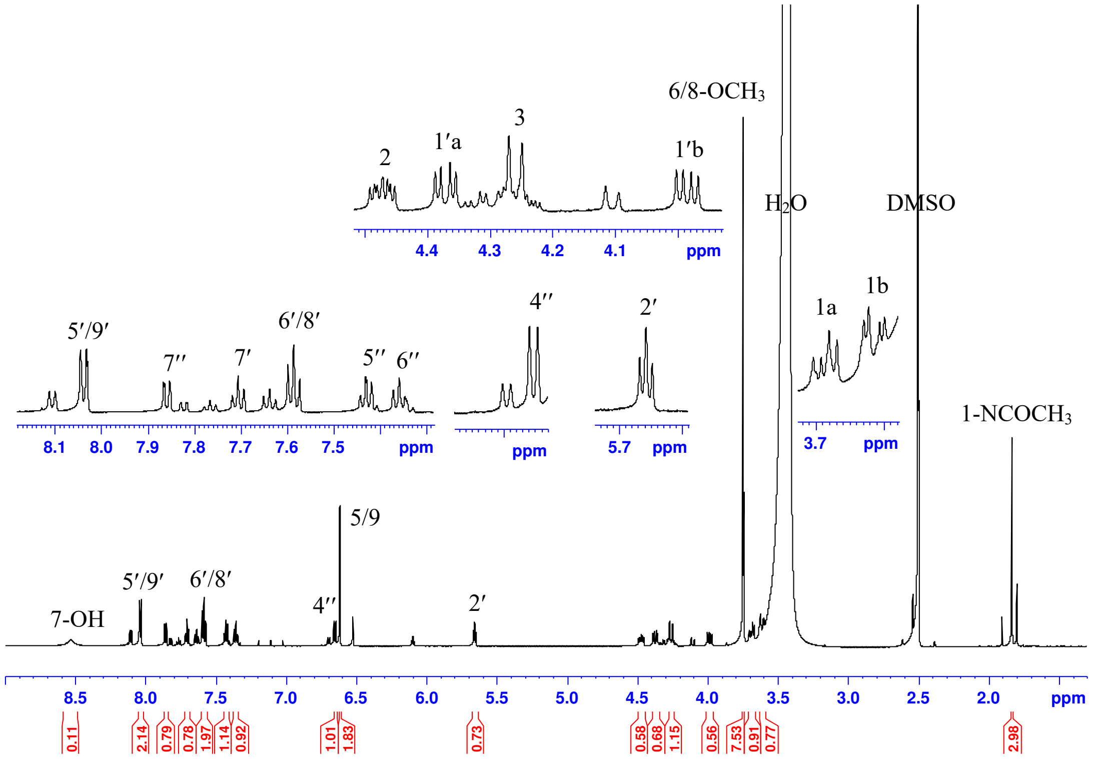

Figure S39. ^{1}H NMR (600 MHz, DMSO-d_6) spectrum for chrysosporazine O (**8**) (major rotamer).

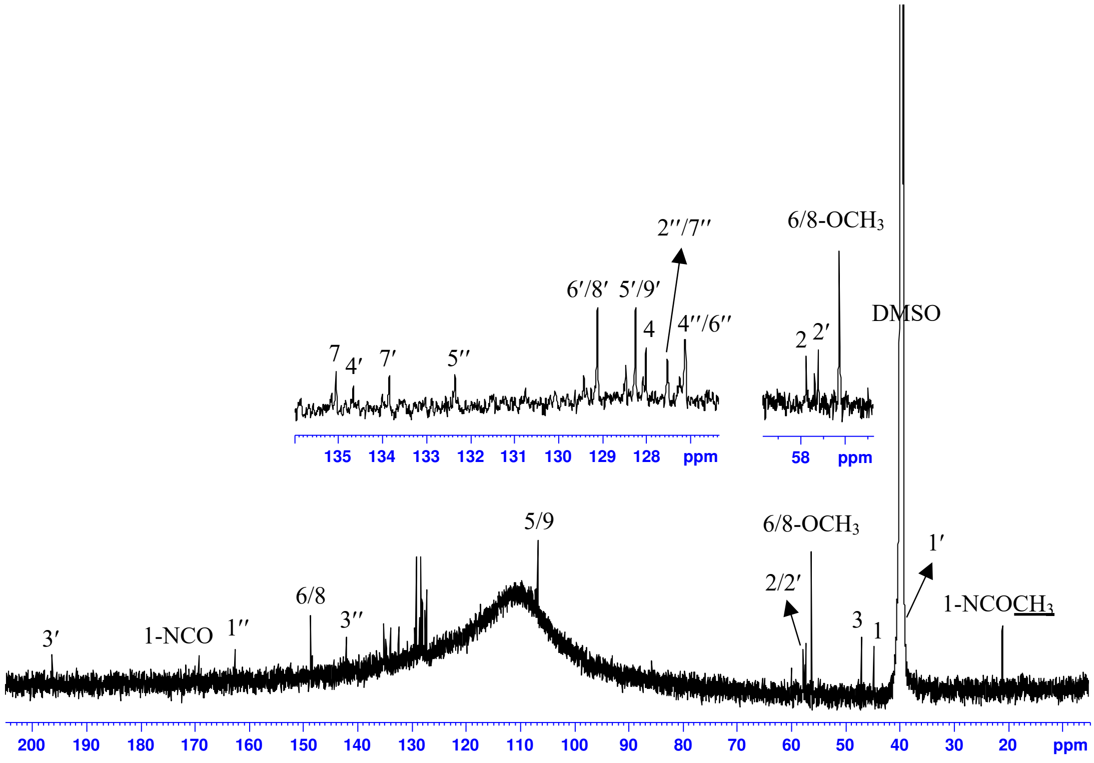

Figure S40. ^{13}C NMR (150 MHz, DMSO-d_6) spectrum for chrysosporazine O (**8**) (major rotamer).

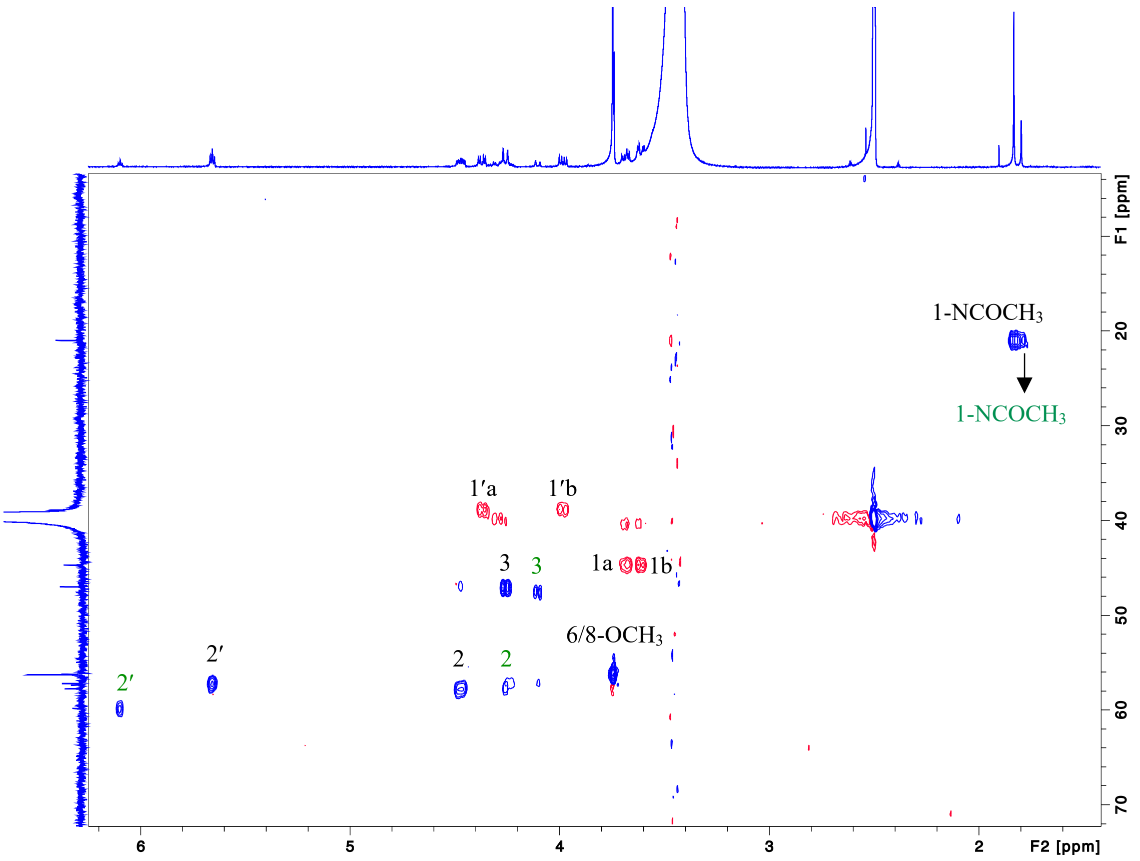

Figure S41. Expanded HSQC NMR (600 MHz, DMSO-d_6) spectrum (part 1) for chrysosporazine O (**8**), major rotamer (labelled black); minor rotamer (labelled green).

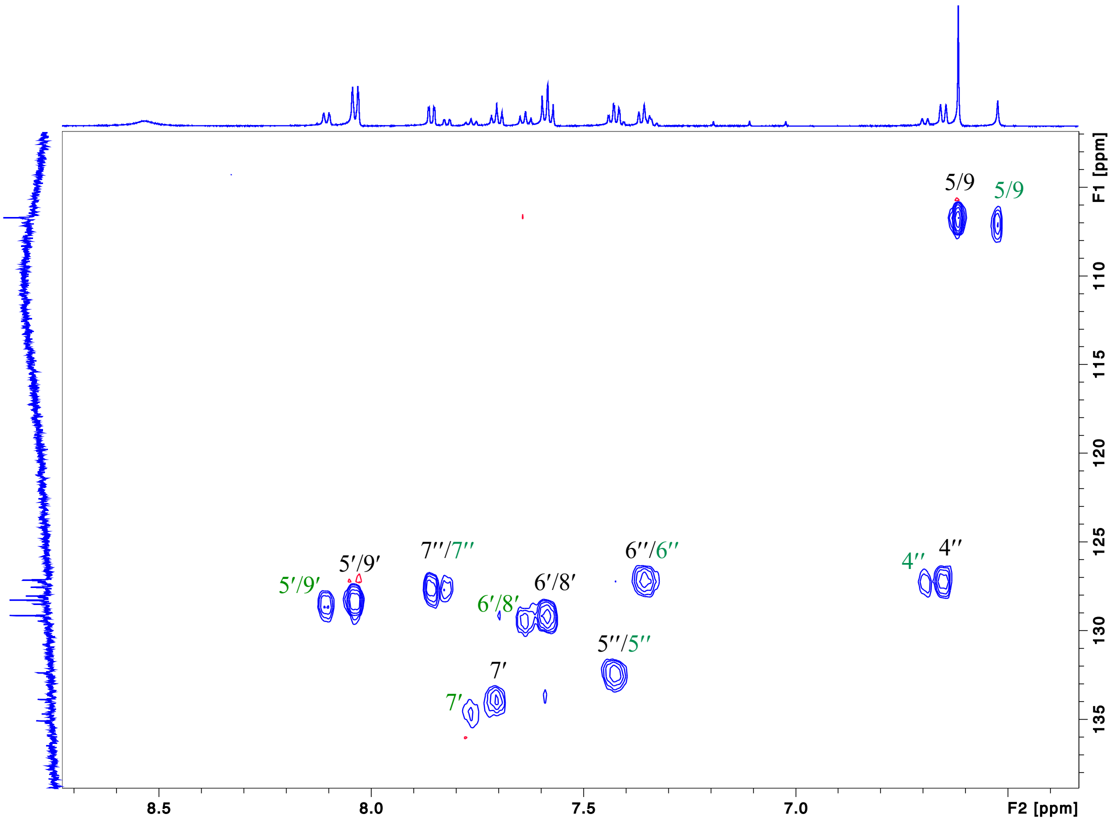

Figure S42. Expanded HSQC NMR (600 MHz, DMSO-d_6) spectrum (part 2) for chrysosporazine O (**8**), major rotamer (labelled black); minor rotamer (labelled green).

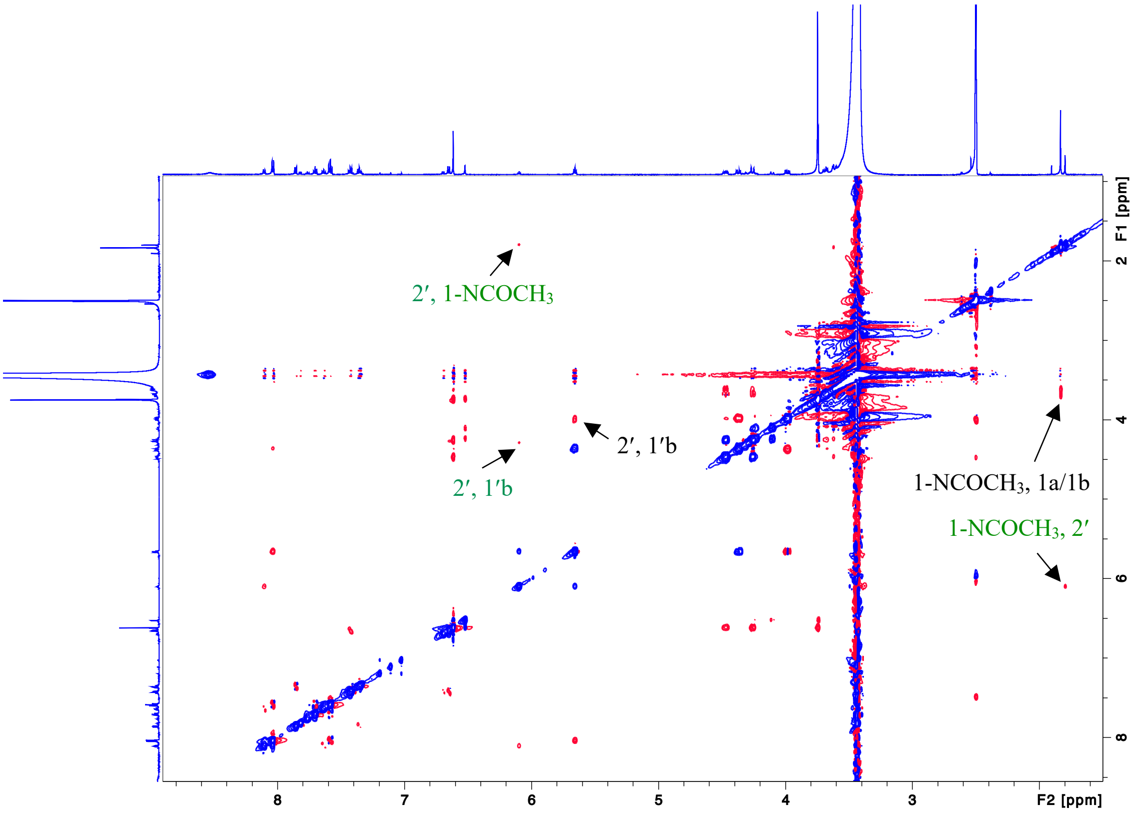

Figure S43. ROESY NMR (600 MHz, DMSO-d_6) spectrum for chrysosporazine O (**8**), major rotamer (labelled black); minor rotamer (labelled green).

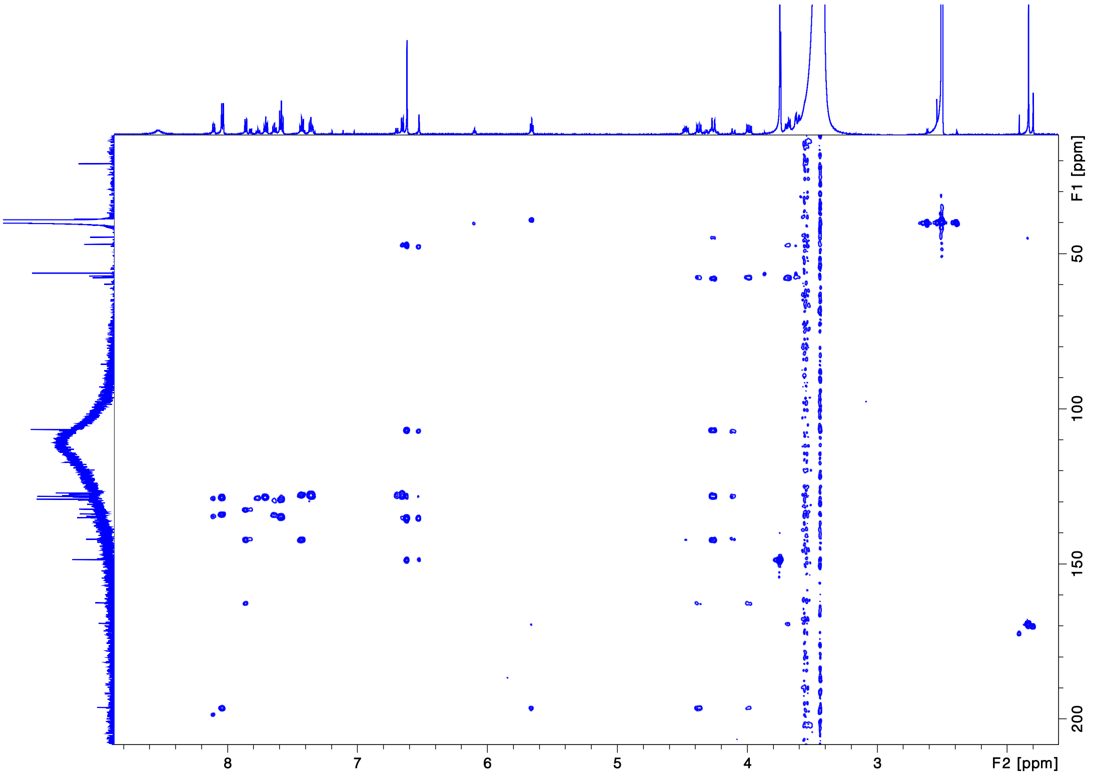

Figure S44. HMBC NMR (600 MHz, DMSO-d_6) spectrum for chrysosporazine O (**8**)

6.9 Chrysosporazine P (9)

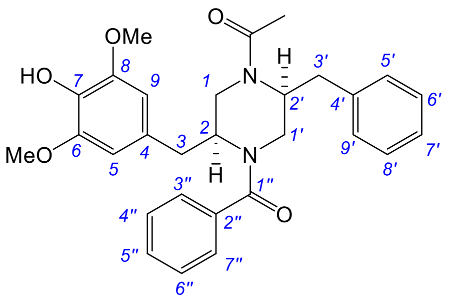

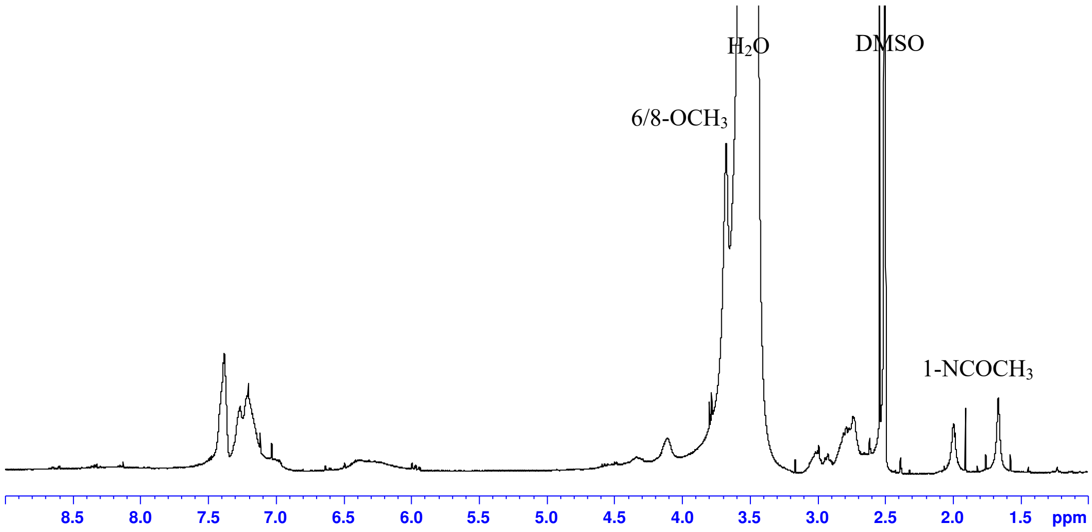

Figure S45. ^{1}H NMR (600 MHz, DMSO-d_6) spectrum for chrysosporazine P (**9**).

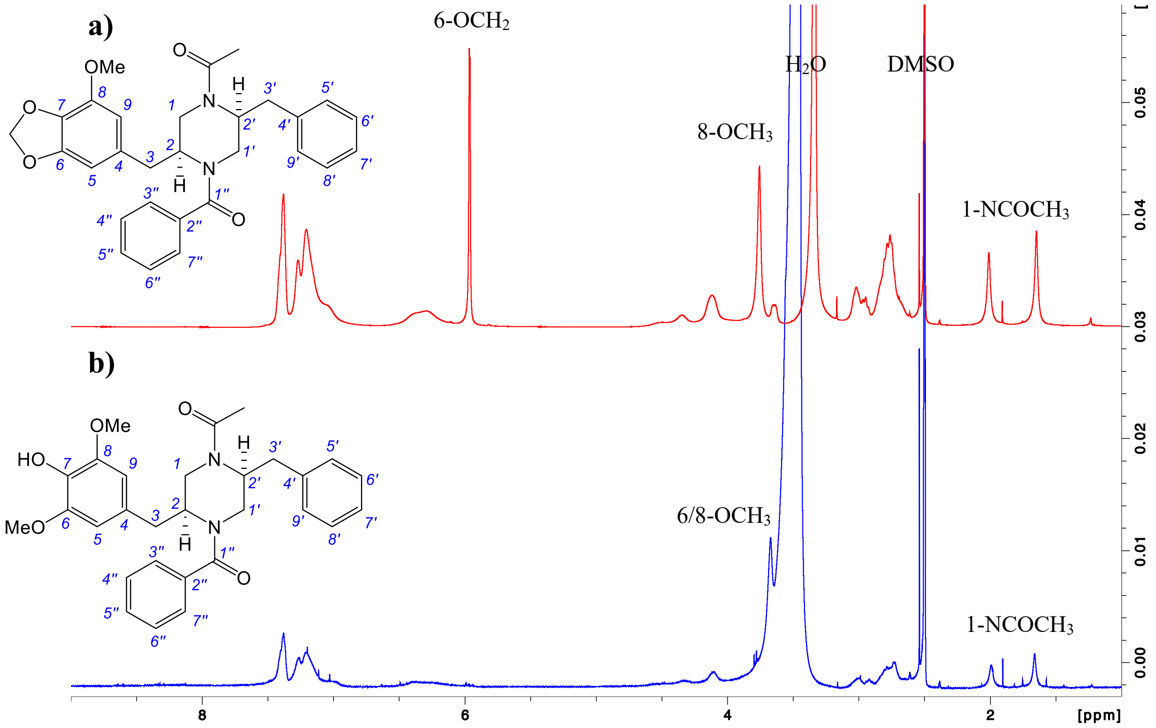

Figure S46. ^{1}H NMR (600 MHz, DMSO-d_6) spectra for a) chrysosporazine D (**14**) and b) chrysosporazine P (**9**).

6.10 Spirochrysosporazine A (10)

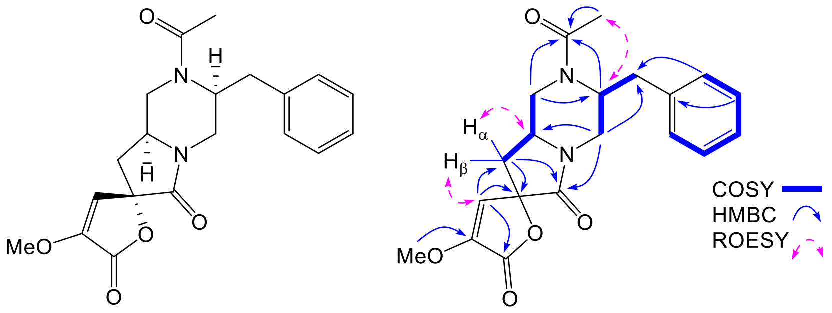

Table S8. 1D and 2D NMR (600 MHz, DMSO-d_6) data for spirochrysosporazine A (**10**) (major rotamer)

Position	δ_H, mult (*J* in Hz)	δ_C	COSY	^{1}H-^{13}C HMBC	ROESY
1	a 4.61, dd (*13.3*, *4.3*)	41.1	1b, 2	2′, 1-NCO	1b, 2
	b 2.77[a], dd (*13.3, 11.7*)		1a, 2	2, 3, 1-NCO	1a
2	3.60, m	51.1	1, 3	---	1a, 3α
3	α 2.51[c], dd (*14.8*, *7.2*)	32.6	2, 3β	1, 2, 4, 7	
	β 2.39, dd (*14.8*, *6.3*)		2, 3α	1, 2, 4, 7	
4	---	85.0	---	---	---
5	---	166.1	---	---	---
6	---	147.0	---	---	---
7	6.65, s	117.9	---	3, 4, 5	3β, 6-OCH_3
1′	a 3.79[b], d (*13.4*)	43.0	1′b	2, 2′, 3′, 1″	1′b
	b 3.18, dd (*13.4, 4.4*)		1′a, 2′	2′, 3′, 1″	1′a, 2′
2′	4.21, m	53.9	1′b, 3′	3′, 1-NCO	1-NCO<u>CH_3</u>, 1′a, 1′b
3′	a 2.93, dd (*13.2*, *6.9*)	34.7	2′, 3′b	1′, 2′, 4′, 5′/9′	3′b, 5′/9′
	b 2.80[a], dd (*13.2, 8.5*)		2′, 3′a	1′, 2′, 4′, 5′/9′	3′a, 5′/9′
4′	---	137.9	---	---	---
5′/9′	7.24 m	129.4	6′/8′	3′, 7′	2′, 3′a, 3′b
6′/8′	7.29, m	128.4	5′/9′, 7′	4′, 6′/8′	---
7′	7.23, m	126.6	6′/8′	5′/9′	---
1″	---	166.7			
1-N<u>CO</u>	---	168.7	---	---	---
1-NCO<u>CH_3</u>	1.69, s	21.0	---	2′, 1-NCO	2′
6-OCH_3	3.80[b], s	58.4	---	6	7

(a–b) overlapping signals, (c) obscured by solvent signal

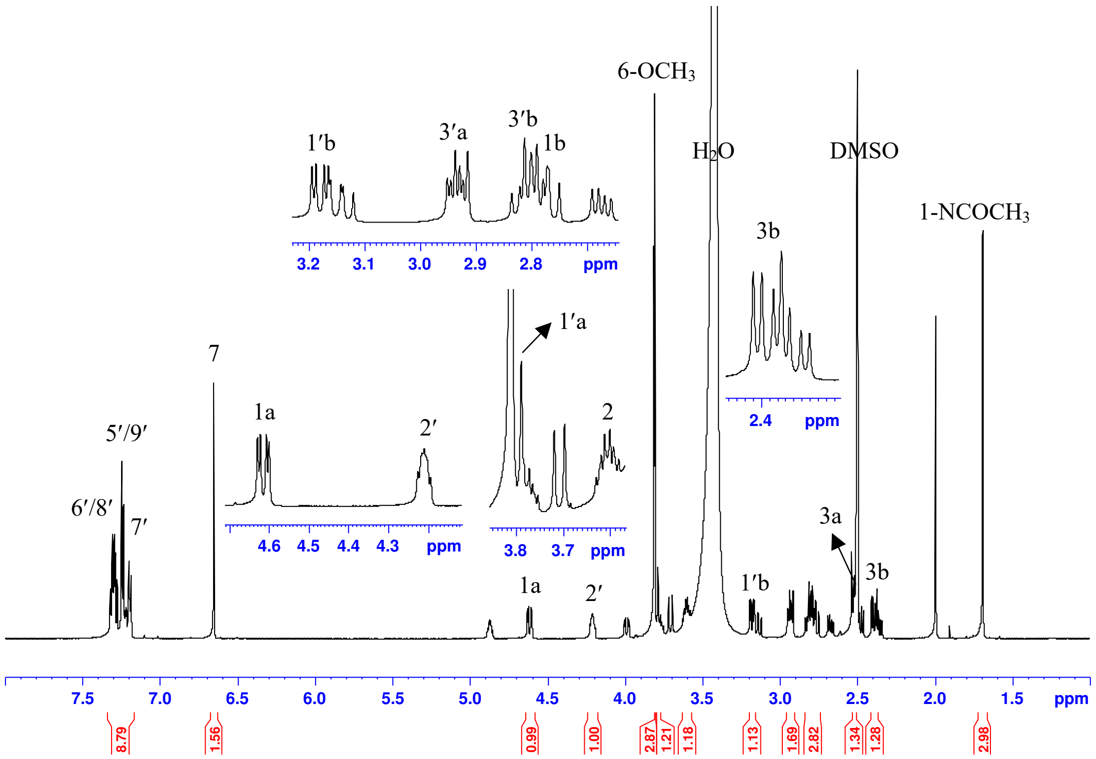

Figure S47. ^{1}H NMR (600 MHz, DMSO-d_6) spectrum for spirochrysosporazine A (**10**).

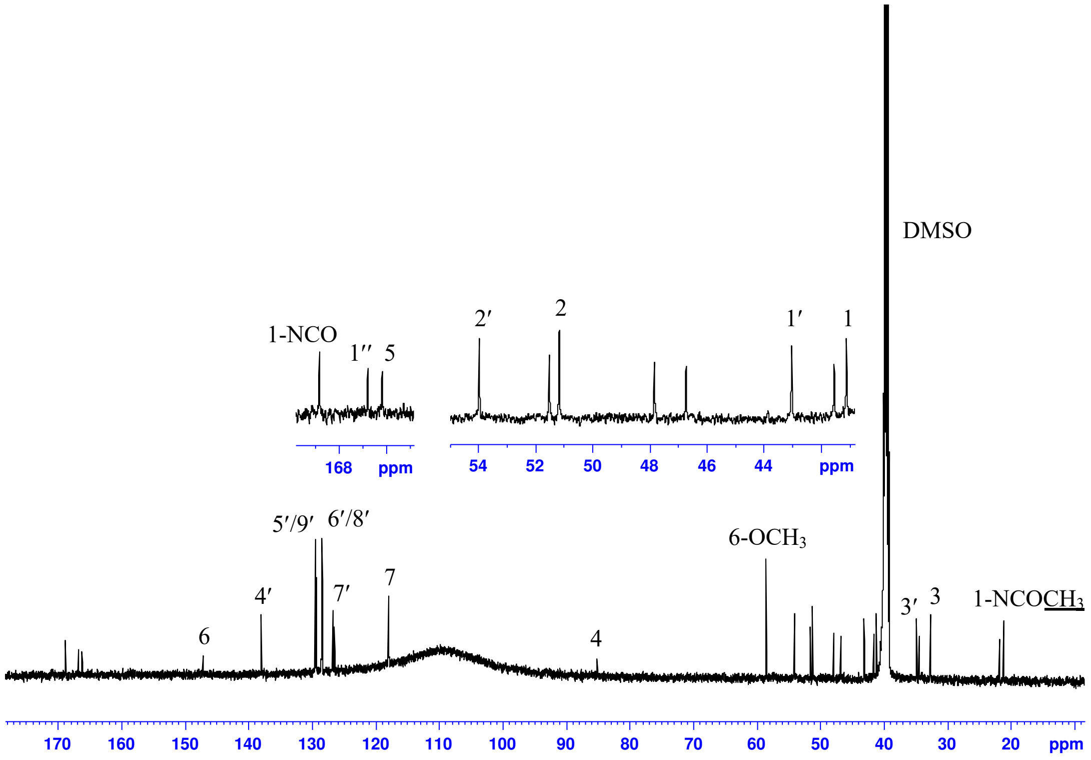

Figure S48. ^{13}C NMR (150 MHz, DMSO-d_6) spectrum for spirochrysosporazine A (**10**).

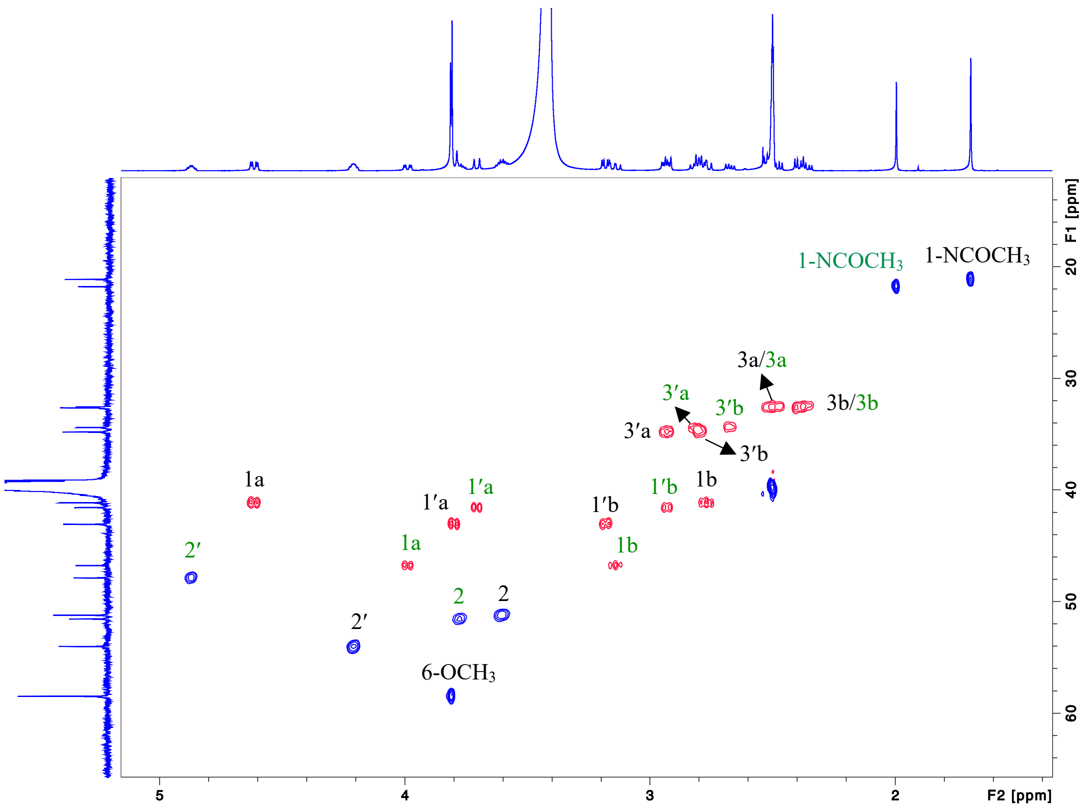

Figure S49. Expanded HSQC NMR (600 MHz, DMSO-d_6) spectrum (part 1) for spirochrysosporazine A (**10**), major rotamer (labelled black); minor rotamer (labelled green).

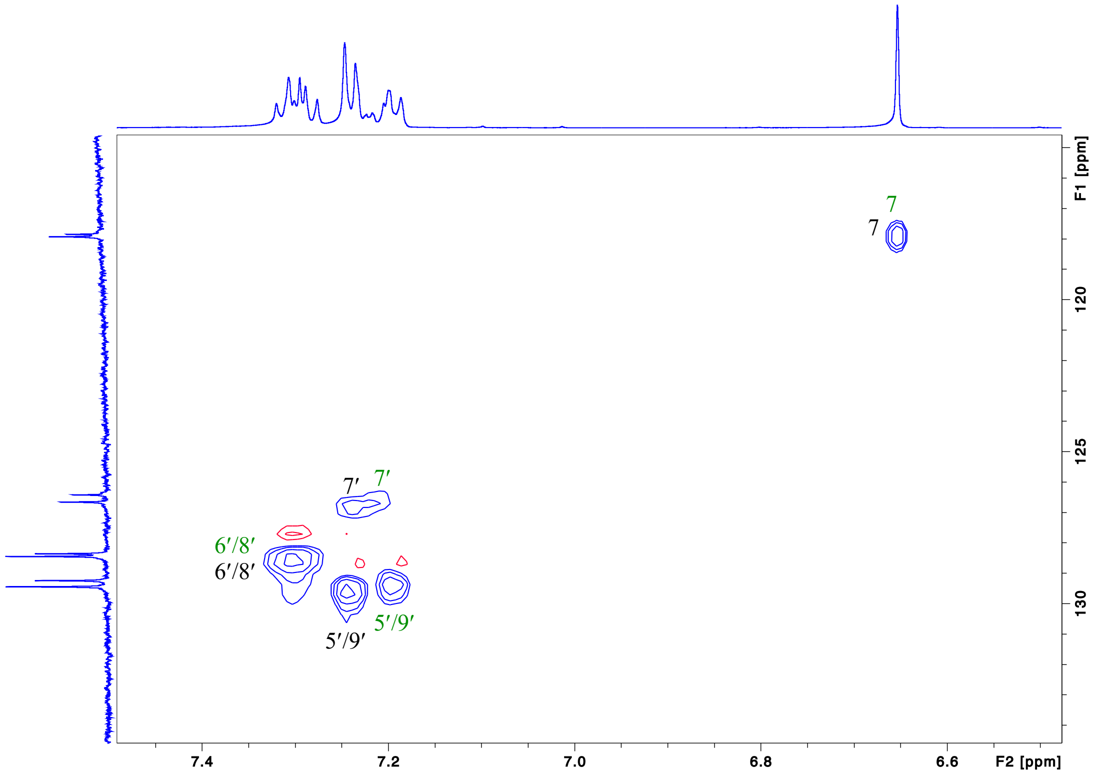

Figure S50. Expanded HSQC NMR (600 MHz, DMSO-d_6) spectrum (part 2) for spirochrysosporazine A (**10**), major rotamer (labelled black); minor rotamer (labelled green).

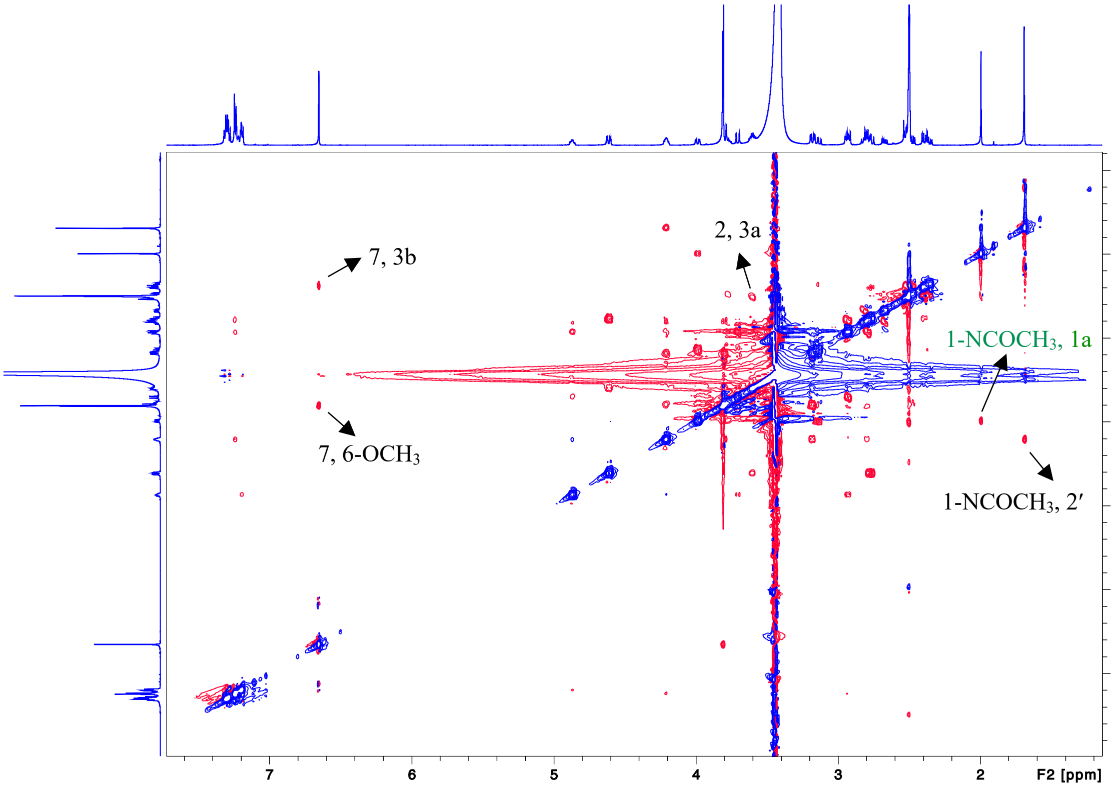

Figure S51. ROESY NMR (600 MHz, DMSO-d_6) spectrum for spirochrysosporazine A (**10**), major rotamer (labelled black); minor rotamer (labelled green).

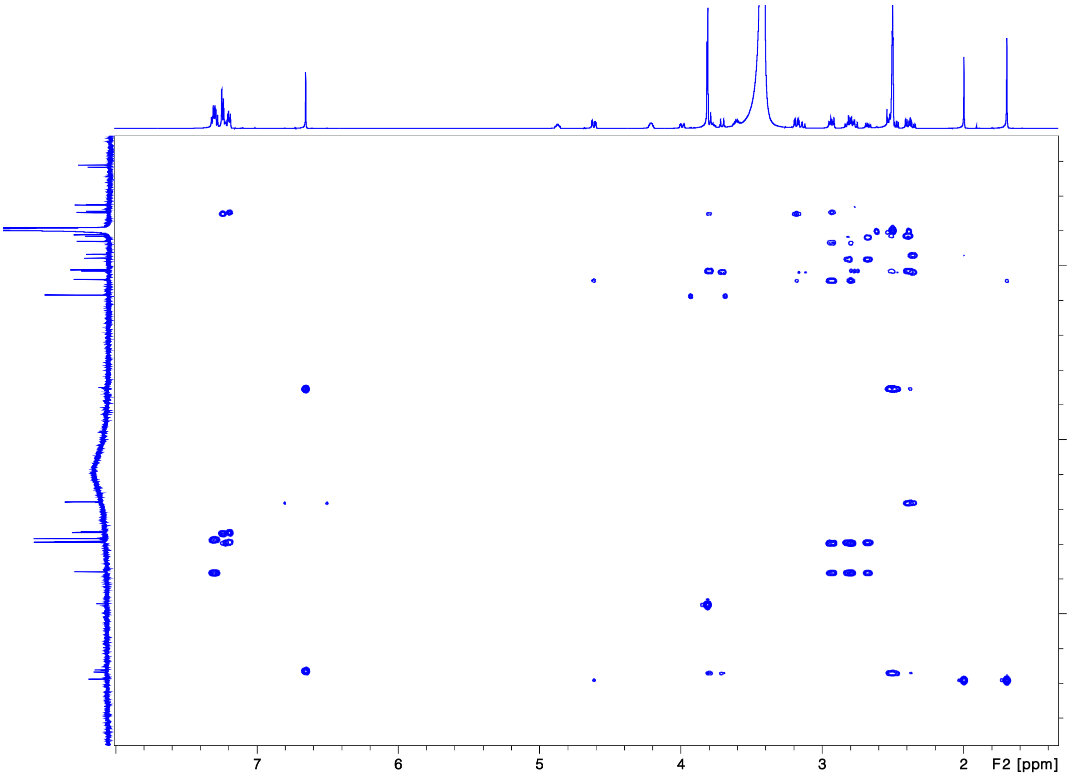

Figure S52. HMBC NMR (600 MHz, DMSO-d_6) spectrum for spirochrysosporazine (**10**)

6.11 Chrysosporazine Q (15)

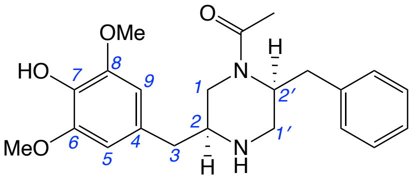

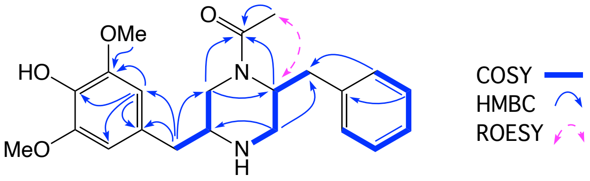

Table S9. 1D and 2D NMR (600 MHz, DMSO-d_6) data for chrysosporazine Q (**15**) (major rotamer)

Position	δ_H, mult (*J* in Hz)	δ_C	COSY	^{1}H-^{13}C HMBC	ROESY
1	a 4.48, dd (*14.5*, *3.2*)	37.1	1b, 2	2, 2′, 1-NCO	1b, 2
	b 2.92[a], m		1a	1-NCO	1a
2	3.27, m	55.9	1′-NH	---	1a, 5/9
3	a 2.88, dd (*13.0*, *6.2*)	36.1	2	1, 2, 4, 5/9	5/9
	b 2.85, dd (*13.0*, *8.0*)		2	1, 2, 4, 5/9	5/9
4	---	125.1	---	---	---
5/9	6.55, br s	106.6	3, 6/8-OCH_3	3, 4, 5/9, 6/8, 7	2, 3, 6/8-OCH_3
6/8	---	148.0	---	---	---
7	---	134.6	---	---	---
1′	a 3.29, m	45.9	1′b	2, 3′	---
	b 3.25, m		2′	---	2′
2′	4.30, m	52.3	3′a	1, 1′, 3′, 4′, 1-NCO	1-NCO<u>CH₃</u>, 1′b, 3′b, 5′/9′
3′	a 3.21, dd (*13.7*, *10.4*)	34.7	2′, 3′b	1′, 2′, 4′, 5′/9′	3′b, 5′/9′
	b 2.93[a], m		2′, 3′a	2′, 4′, 5′/9′	3′a, 5′/9′
4′	---	137.6	---	---	---
5′/9′	7.25 m	129.3	6′/8′	3′, 7′	2′, 3′a, 3′b
6′/8′	7.31, m	128.5	5′/9′, 7′	4′, 6′/8′	---
7′	7.25, m	126.8	6′/8′	5′/9′	---
1-N<u>CO</u>	---	168.4	---	---	---
1-NCO<u>CH$_3$</u>	1.46, s	20.3	---	2′, 1-NCO	2′
6/8-OCH_3	3.76, s	55.9	---	6/8	---
7-OH	8.03, br s	---	---	---	---
1′-NH	9.06, m	---	1′, 2	---	---

(a) overlapping signals

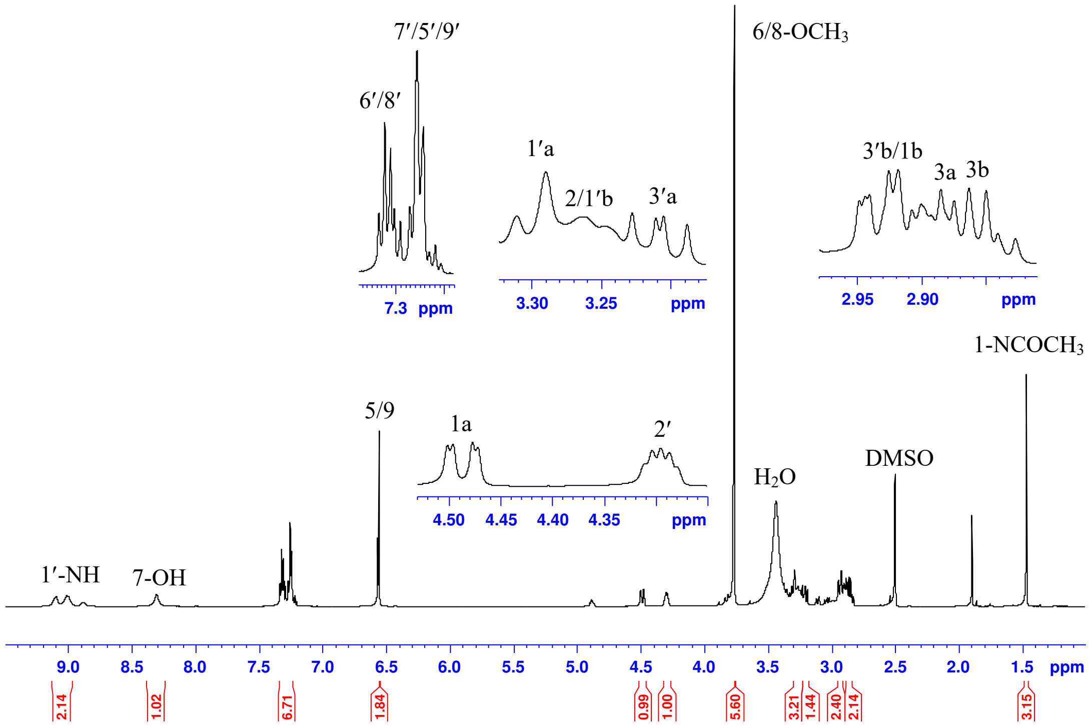

Figure S53. ^{1}H NMR (600 MHz, DMSO-d_6) spectrum for chrysosporazine Q (**15**).

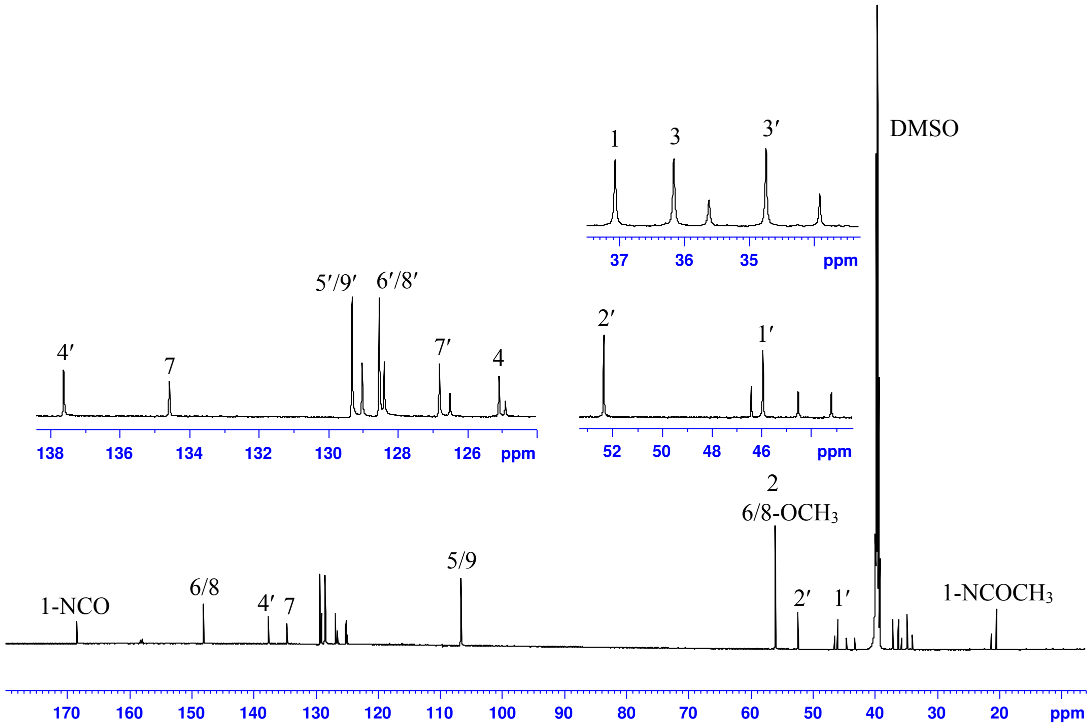

Figure S54. ^{13}C NMR (150 MHz, DMSO-d_6) spectrum for chrysosporazine Q (**15**).

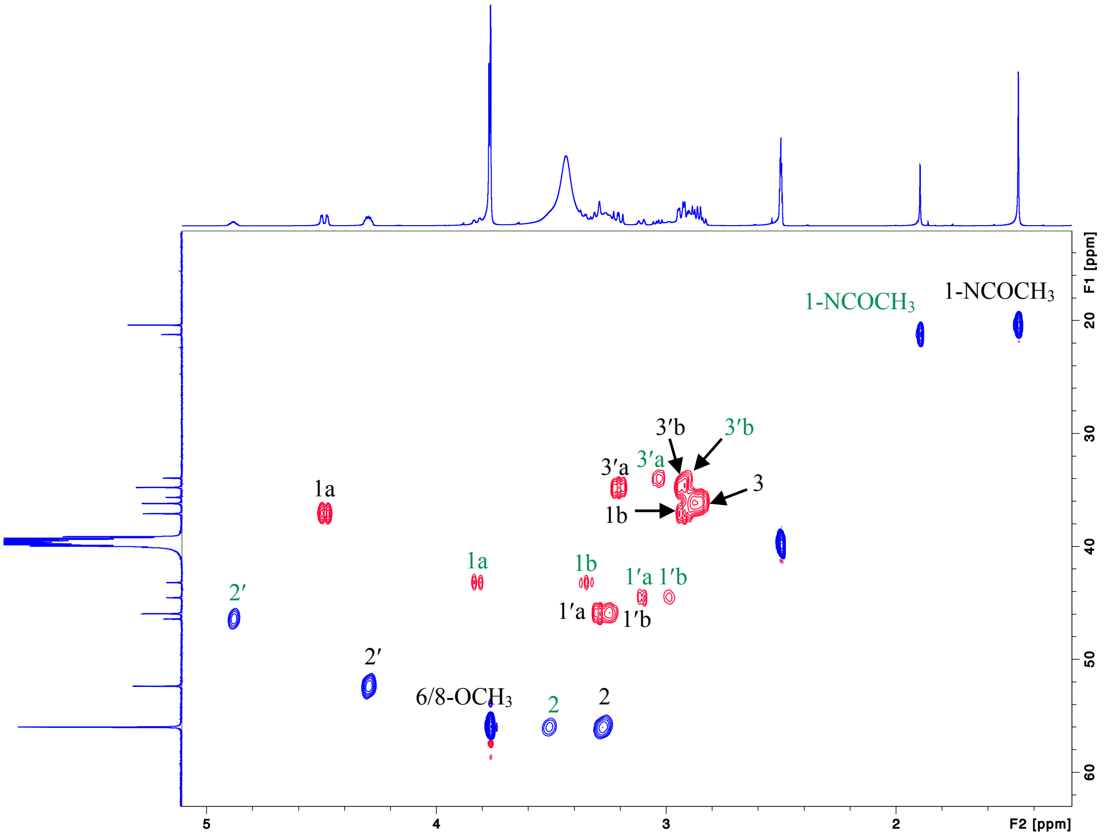

Figure S55. Expanded HSQC NMR (600 MHz, DMSO-d_6) spectrum (part 1) for chrysosporazine Q (**15**), major rotamer (labelled black); minor rotamer (labelled green).

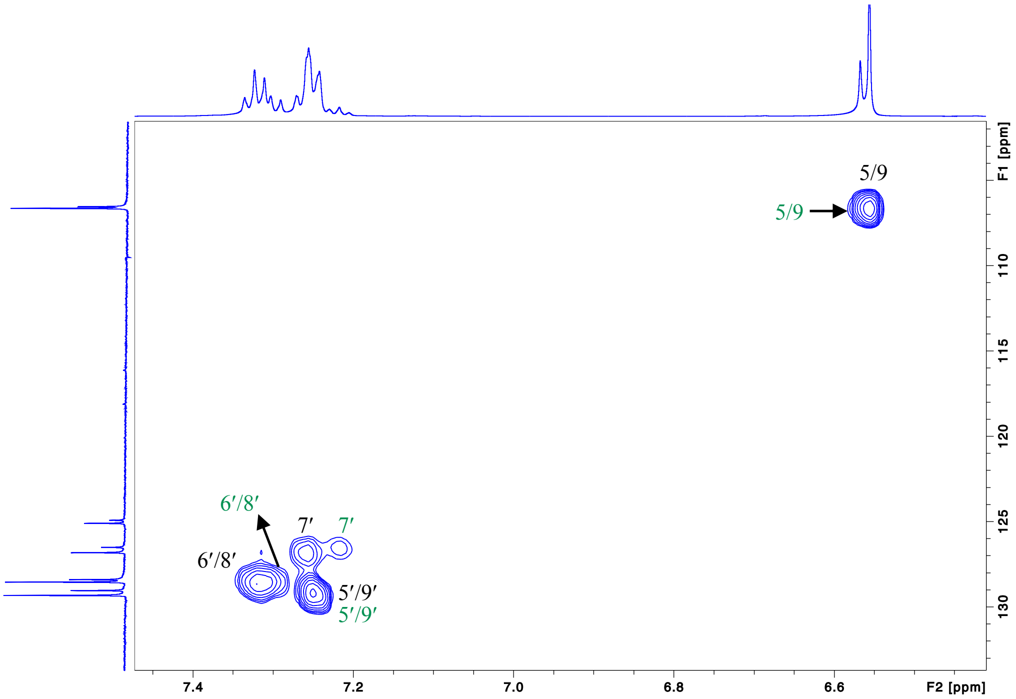

Figure S56. Expanded HSQC NMR (600 MHz, DMSO-d_6) spectrum (part 2) for chrysosporazine Q (**15**), major rotamer (labelled black); minor rotamer (labelled green).

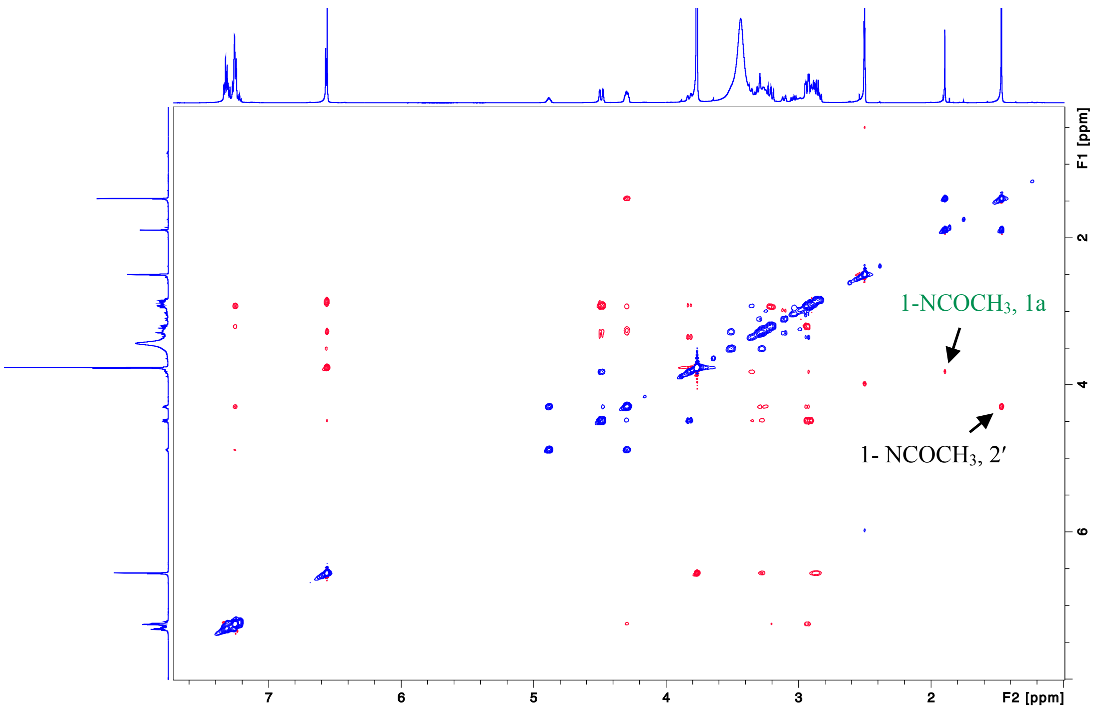

Figure S57. ROESY NMR (600 MHz, DMSO-d_6) spectrum for chrysosporazine Q (**15**), major rotamer (labelled black); minor rotamer (labelled green).

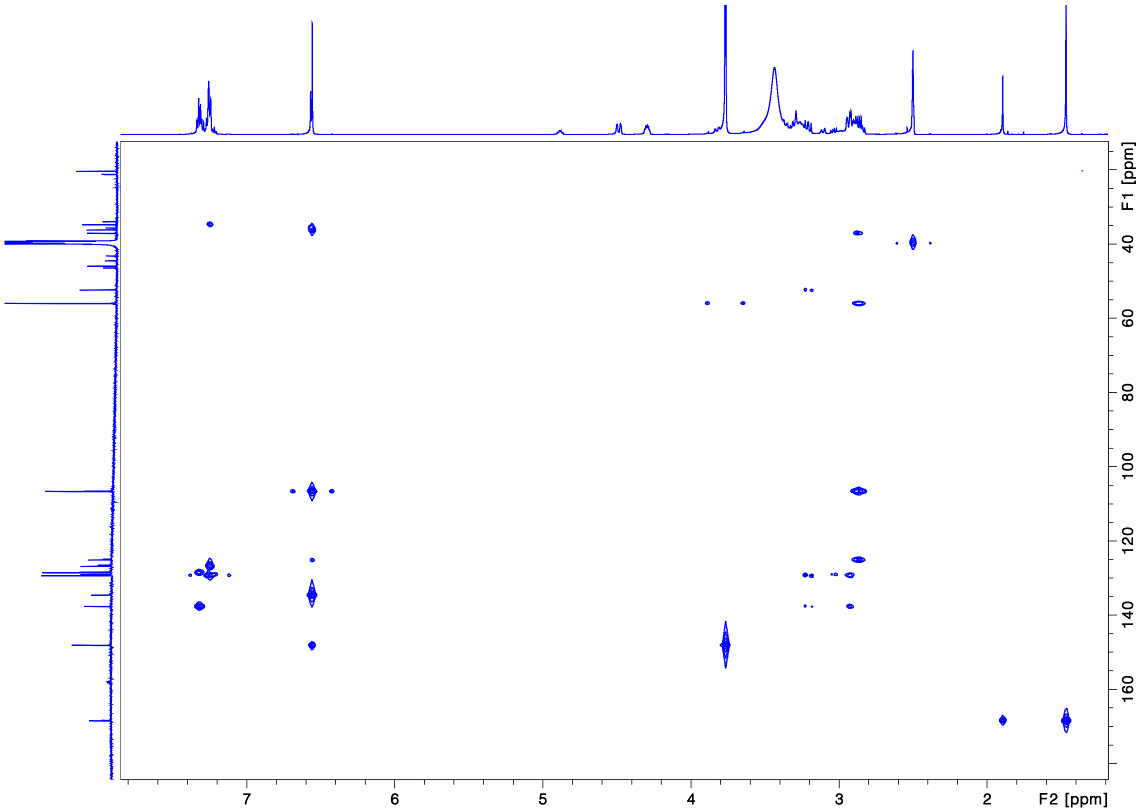

Figure S58. HMBC NMR (600 MHz, DMSO-d_6) spectrum for chrysosporazine Q (**15**)

7 Comparative NMR analysis of natural chrysosporazines to corresponding azachrysosporazines

Table S10. Chrysosporazine A (11), azachrysosporazines A1 (1) and A2 (2)

Position	11		1		2	
	δ_H, mult (*J* in Hz)	δ_C	δ_H, mult (*J* in Hz)	δ_C	δ_H, mult (*J* in Hz)	δ_C
1	a 4.21, dd (*13.1, 2.8*)<br>b 2.93, dd (*13.1, 10.1*)	42.3	a 4.26, ddd (*13.3,3.0, 1.2*)<br>b 2.99, dd (*13.3, 9.8*)	42.3	a 4.21, ddd (*13.1,3.0, 1.2*)<br>b 2.97, dd (*13.1, 9.8*)	42.1
2	4.44, ddd (*12.2, 10.4, 3.1*)	56.7	4.47, ddd (*12.3, 9.8, 3.0*)	56.9	4.49, ddd (*12.1, 9.8, 3.0*)	56.5[a]
3	4.36, d (*12.2*)	46.1	4.51, d (*12.3*)	48.8	4.46, d (*12.1*)	45.4
4	---	132.6	---	132.8	---	131.1
5	6.67, br s	102.9	6.57, d (*1.2*)	103.4	6.68, d (*0.9*)	103.0[b]
6	---	148.9	---	148.5	---	149.0
7	---	134.3	---	134.0	---	134.6
8	---	143.4	---	143.1	---	143.6
9	6.73, br s	109.6	6.65, d (*1.2*)	110.1	6.74, d (*0.9*)	109.6[b]
1′	6.83, d (*6.8*)	107.0	6.80, d (*6.8*)	106.7	6.81, d (*6.8*)	106.4
2′	6.51, d (*6.8*)	112.0	6.55, dd (*6.8, 1.2*)	112.6	6.56, dd (*6.8, 1.2*)	112.7
1″	---	158.7	---	158.5	---	157.4
2″	---	127.19[a]	---	123.2	---	123.1
3″	---	140.8	---	158.6	---	149.8
4″	6.61, d (*7.6*)	126.7	**N**	---	6.66, d (*5.0*)	121.5
5″	7.45, ddd (*7.6, 7.6, 1.3*)	132.6	8.56, dd (*4.8, 1.8*)	152.4	8.59, d (*5.0*)	152.5
6″	7.38, dd (*7.6, 7.6*)	127.21[a]	7.43, ddd (*7.8, 4.8, 0.8*)	122.9	**N**	---
7″	7.96, dd (*7.6, 1.3*)	127.6	8.26, dd (*7.8, 1.8*)	135.4	9.03, s	147.9
1-NCO	---	166.5	---	166.7	---	166.7
1-NCOCH$_3$	2.09, s	20.8	2.10, s	20.8	2.10, s	20.8
6-OCH$_2$	6.05/6.06, ABq	101.4	6.02/6.00, AB$_q$	101.2	6.06/6.05, AB$_q$	101.6
8-OCH$_3$	3.82, s	56.5	3.80, s	56.4	3.81, s	56.5[a]

(a) overlapping resonances within the same column, (b) signals determined from HMBC correlations

Table S11. Chrysosporazine B (12) and azachrysosporazine B1 (3)

	**12**		**3**	
**Position**	δ_H, mult (*J* in Hz)	δ_C	δ_H, mult (*J* in Hz)	δ_C
1	a 3.82, dd (*13.8, 3.9*)<br>b 3.46, dd (*13.8, 8.7*)	46.4	a 3.98, dd (*12.9, 3.8*)<br>b 3.51[a]	47.3
2	4.20, ddd (*8.5*, *8.0, 3.9*)	58.8	4.21, ddd (*9.8, 5.4, 3.8*)	59.2
3	4.39, d (*8.0*)	45.0	4.46, d (*5.4*)	47.2
4	---	135.2	---	135.3
5	6.45, br s	102.2	6.31, d (*1.4*)	102.0
6	---	148.8	---	148.6
7	---	134.0	---	133.8
8	---	143.3	---	143.1
9	6.63, d (*1.0*)	108.7	6.55, d (*1.4*)	108.6
1′	a 4.78, dd (*14.1, 1.3*)<br>b 3.48, dd (*14.1, 5.5*)	41.5	a 4.79, dd (*14.1*, *1.3*)<br>b 3.51[a]	42.6
2′	5.86, dd (*5.5, 1.3*)	54.8	5.89, dd (*5.7, 1.3*)	54.3
3′	---	196.9	---	197.6
4′	---	134.7	---	134.7
5′/9′	7.97, dd (*7.8, 1.1*)	128.2	7.97, m	128.2
6′/8′	7.56, dd (*7.8, 7.5*)	128.8	7.56, m	128.9
7′	7.68, t (*7.5*)	133.4	7.68, m	133.5
1″	---	162.6	---	161.9
2″	---	127.2	---	122.8
3″	---	140.2	---	158.1
4″	6.89, d (*7.6*)	127.9	**N**	---
5″	7.47, ddd (*7.6, 7.6, 1.2*)	132.5	8.64, dd (*4.7, 1.8*)	153.0
6″	7.37, dd (*7.6, 7.6*)	127.1	7.44, dd (*7.8, 4.7*)	123.1
7″	7.86, dd (*7.6, 1.2*)	127.5	8.18, dd (*7.8, 1.8*)	135.6
1-NCO	---	169.9	---	170.5
1-NCOCH_3	1.95, s	21.2	2.00, s	21.4
6-OCH_2	5.99, s	101.3	5.95/5.94, AB_q	101.3
8-OCH_3	3.80, s	56.4	3.78, s	56.4

(a) overlapping resonances and obscured by solvent signals

Table S12. Chrysosporazine C (13), azachrysosporazines C1 (4) and C2 (5)

	13		4		5	
**Position**	δ_H, mult (*J* in Hz)	δ_C	δ_H, mult (*J* in Hz)	δ_C	δ_H, mult (*J* in Hz)	δ_C
1	a 4.20, dd (*13.8, 3.9*)<br>b 2.95[a], m	39.9[b]	a 4.29, dd (*13.8, 3.8*)<br>b 3.04[a], dd (*13.8, 11.4*)	40.1[b]	a 4.19, dd (*13.8, 3.9*)<br>b 2.99, dd (*13.8, 11.0*)	39.8[b]
2	3.85, ddd (*11.0, 11.0, 3.9*)	57.3	3.88, ddd (*11.4, 10.0, 3.8*)	58.0	3.91, ddd (*11.2, 11.0, 3.9*)	56.9
3	4.38, d (*11.0*)	46.4	4.49, d (*10.0*)	48.8	4.46, d (*11.2*)	45.7
4	---	134.2	---	134.6	---	132.6
5	6.61, d (*1.2*)	102.8	6.49, d (*1.4*)	103.1	6.64, d (*1.3)*	103.0
6	---	148.8	---	148.5	---	148.9
7	---	134.1	---	133.8	---	134.4
8	---	143.3	---	143.1	---	143.5
9	6.72, d (*1.2*)	109.4	6.63, d (*1.4*)	109.6	6.73, d (*1.3)*	109.5
1′	a 4.58, dd (*13.5, 1.2*)<br>b 2.95[a], dd *(13.5, 4.2)*	44.7	a 4.56, dd (*13.4, 1.1*)<br>b 3.00, m	45.0	a 4.56, dd (*13.5, 1.2*)<br>b 2.95, dd (*13.5, 4.1*)	44.5
2′	4.25, m	54.5	4.25, m	54.6	4.27, m	54.3
3′	a 3.04, dd (*13.3, 8.9*)<br>b 2.92, dd *(13.3, 6.1)*	34.9	a 3.03[a], m<br>b 2.90, dd (*13.4, 5.8*)	34.9	a 3.06, dd (*13.4, 8.8*)<br>b 2.93, dd (*13.4, 5.8*)	34.8
4′	---	138.2	---	138.2	---	138.2
5′/9′	7.26, m	129.4	7.26, m	129.4	7.26, m	129.4
6′/8′	7.30, m	128.3	7.30, m	128.4	7.30, m	128.4
7′	7.24, m	126.5	7.22, m	126.5	7.23, m	126.5
1″	---	164.1	---	163.5	---	162.9
2″	---	127.3	---	122.9	---	123.0
3″	---	140.6	---	158.4	---	149.3
4″	6.71, dd (*7.7, 1.2*)	126.9	**N**	---	6.72, br s	121.5
5″	7.46, ddd (*7.7, 7.7, 1.3*)	132.3	8.60, dd (*4.7, 1.8*)	152.5	8.60, br s	152.4
6″	7.39, dd (*7.7, 7.7*)	126.8	7.45, dd (*7.8, 4.7*)	122.7	**N**	---
7″	8.03, dd (*7.7, 1.3*)	127.5	8.18, dd (*7.8, 1.8*)	135.5	9.08, br s	148.3
1-N<u>CO</u>	---	168.3	---	168.4	---	168.3
1-NCO<u>CH$_3$</u>	1.59, s	20.6	1.60, s	20.7	1.59, s	20.6
6-OCH$_2$	6.04, s	101.4	5.99/5.99, AB$_q$	101.2	6.05, br s	101.5
8-OCH$_3$	3.83, s	56.4	3.80, s	56.4	3.82, s	56.4

(a) overlapping resonances within the same column, (b) obscured by solvent signal

Table S13. chrysosporazine N (7) and chrysosporazine O (8)

	**7**		**8**	
**Position**	δ_H, mult (*J* in Hz)	δ_C	δ_H, mult (*J* in Hz)	δ_C
1	δ_H, mult (*J* in Hz)	46.0	a 3.68, dd (*14.3, 4.1*)	44.6
	a 3.77, dd (*13.6, 4.1*)		b 3.61, dd (*14.3, 6.8*)	
2	b 3.43[a]	58.5	4.47, ddd (*12.4, 6.8, 4.1*)	57.7
3	4.27, ddd (*12.4, 9.0, 4.1*)	45.4	4.25, d (*12.4*)	46.9
4	4.36, d (*9.0*)	130.1	---	128.0
5/9	---	106.3	6.61, s	106.6
6/8	6.54, s	148.3	---	148.5
7	---	134.7[b]	---	135.0
1′	---	40.9	a 4.37, dd (*14.1, 7.1*)	38.8
	a 4.76, dd (*14.3, 1.9*)		b 3.98, dd (*14.1, 4.5*)	
2′	b 3.52, dd (*14.3, 5.5*)	55.1	5.67, dd (*7.1, 4.5*)	57.1
3′	5.83, dd (*5.5, 1.9*)	196.6	---	196.3
4′	---	134.7[b]	---	134.6
5′/9′	---	128.2	8.03, dd (*8.4, 1.3*)	128.2
6′/8′	7.97, dd (*8.1, 1.0*)	128.9	7.58, dd (*7.8, 7.6*)	129.1
7′	7.55, dd (*8.1, 7.6*)	133.5	7.70, t (*7.8*)	133.8
1″	7.67, t (*7.6*)	162.8	---	162.5
2″	---	127.4	---	127.5[b]
3″	---	140.9	---	142.0
4″	---	127.7	6.65, d (*7.8*)	127.1[c]
5″	6.82, d (*8.7*)	132.4	7.42, ddd (*7.8, 7.4, 1.3*)	132.3
6″	7.45, ddd (*8.7, 7.4, 1.5*)	127.0	7.35, dd (*7.6, 7.4*)	127.1[c]
7″	7.35, dd (*7.7, 7.4*)	127.5	7.85, dd (*7.6, 1.3*)	127.5[b]
1-N<u>CO</u>	7.85, dd (*7.7, 1.5*)	169.9	---	169.1
1-NCO<u>CH$_3$</u>	---	21.2	1.83, s	20.9
6/8-OCH$_3$	1.91, s	56.1	3.74, s	56.2
7-OH	3.69, s	---	8.53, br s	---

(a) obscured by solvent signal, (b–c) overlapping resonances within the same column

8 CD spectra for chrysosporazines A–D (11–14) and 1–11

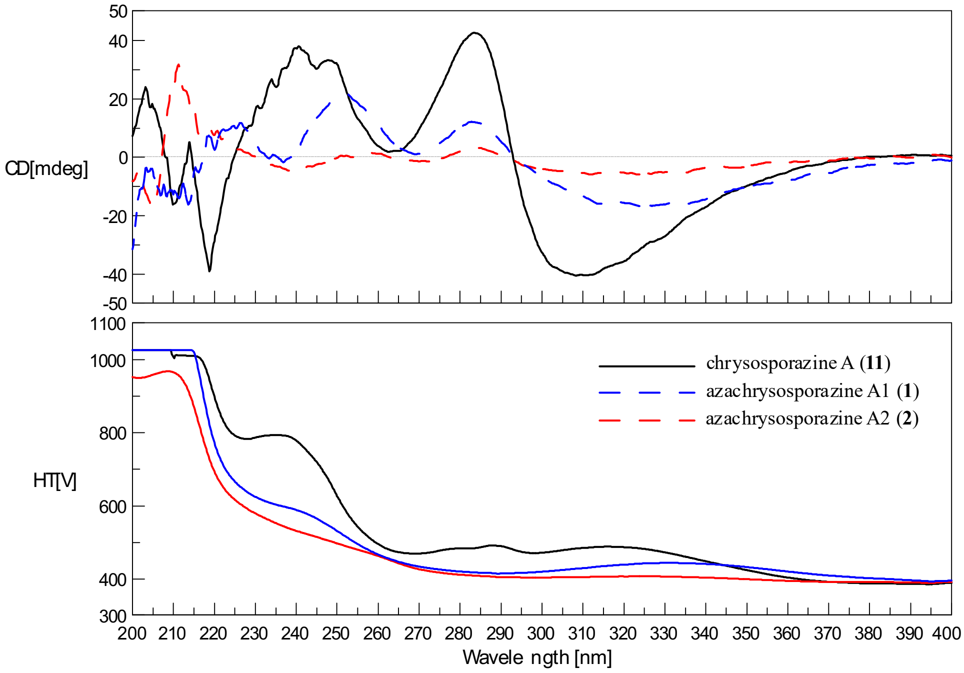

Figure S59. CD (top) and UV (bottom) spectra for chrysosporazine A (**11**), azachrysosporazine A1 (**1**) and azachrysosporazine A2 (**2**) in MeOH (0.05 g%).

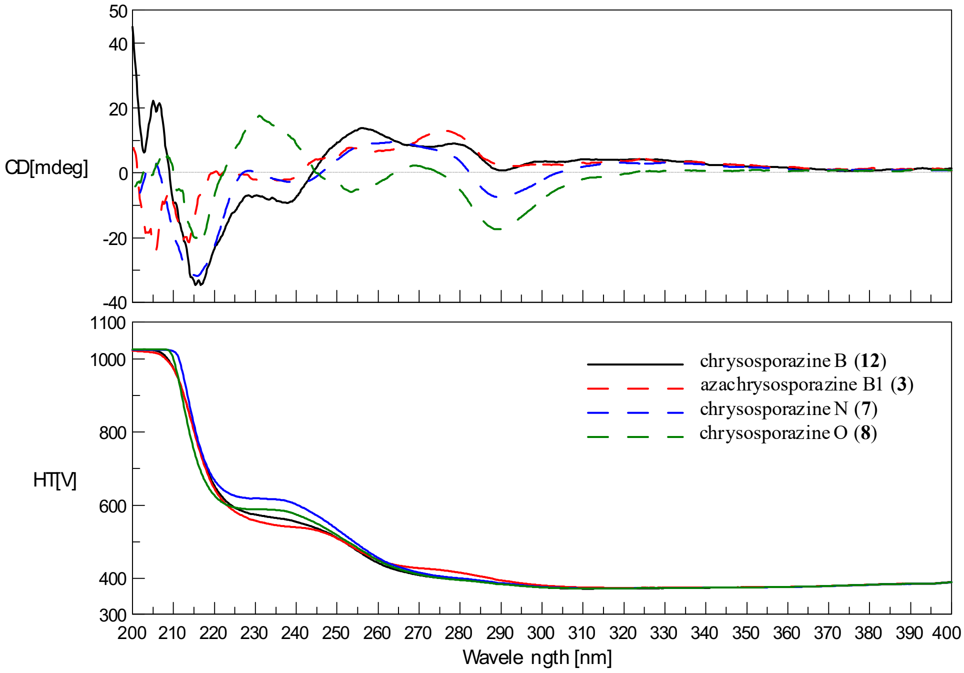

Figure S60. CD (top) and UV (bottom) spectra for chrysosporazine B (**12**), azachrysosporazine B1 (**3**), chrysosporazine N (**7**) and chrysosporazine O (**8**) in MeOH (0.05 g%).

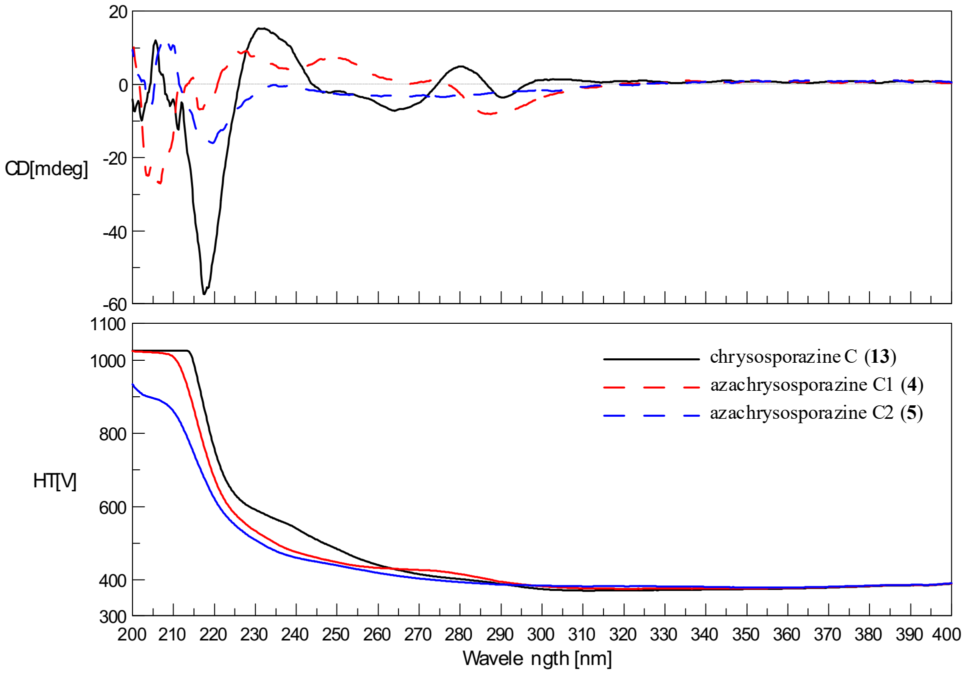

Figure S61. CD (top) and UV (bottom) spectra for chrysosporazine C (**13**), azachrysosporazine C1 (**4**) and azachrysosporazine C2 (**5**) in MeOH (0.05 g%).

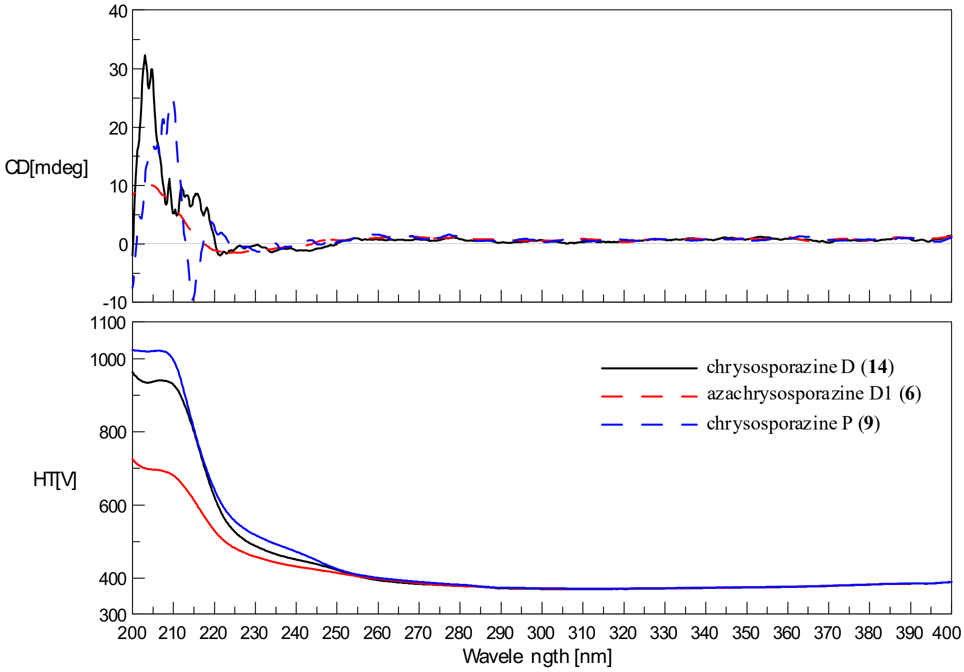

Figure S62. CD (top) and UV (bottom) spectra for chrysosporazine D (**14**), azachrysosporazine D1 (**6**) and chrysosporazine P (**9**) in MeOH (0.05 g%).

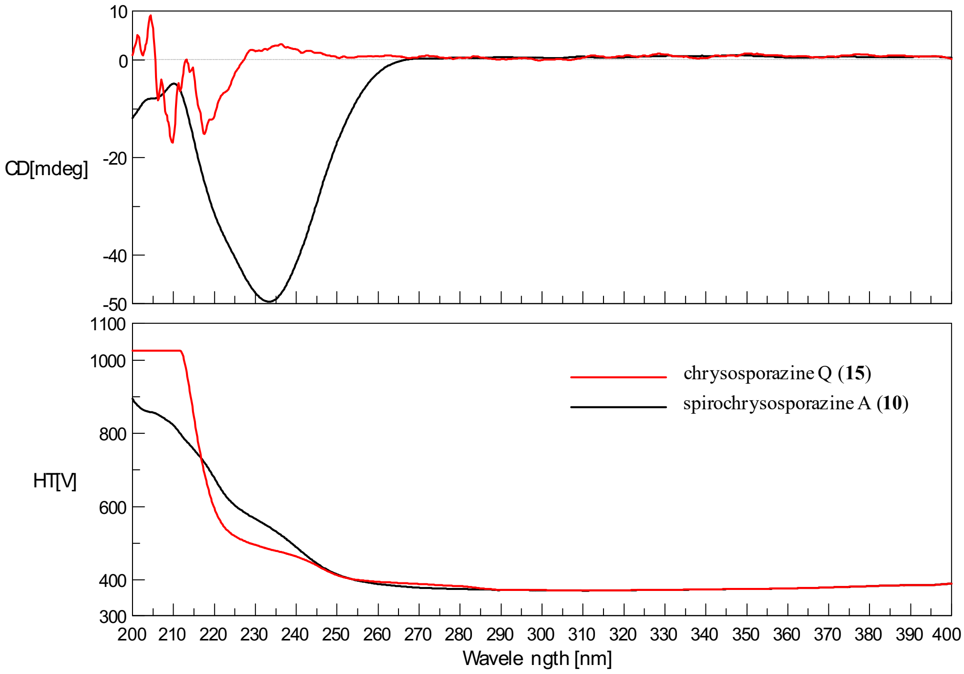

Figure S63. CD (top) and UV (bottom) spectra for chrysosporazines Q (**15**) and spirochrysosporazine A (**10**) in MeOH (0.05 g%).

9 Acid hydrolysis of azachrysosporazine D1 (6) and chrysosporazine P (9)

9.1 Azachrysosporazine D1 (6)

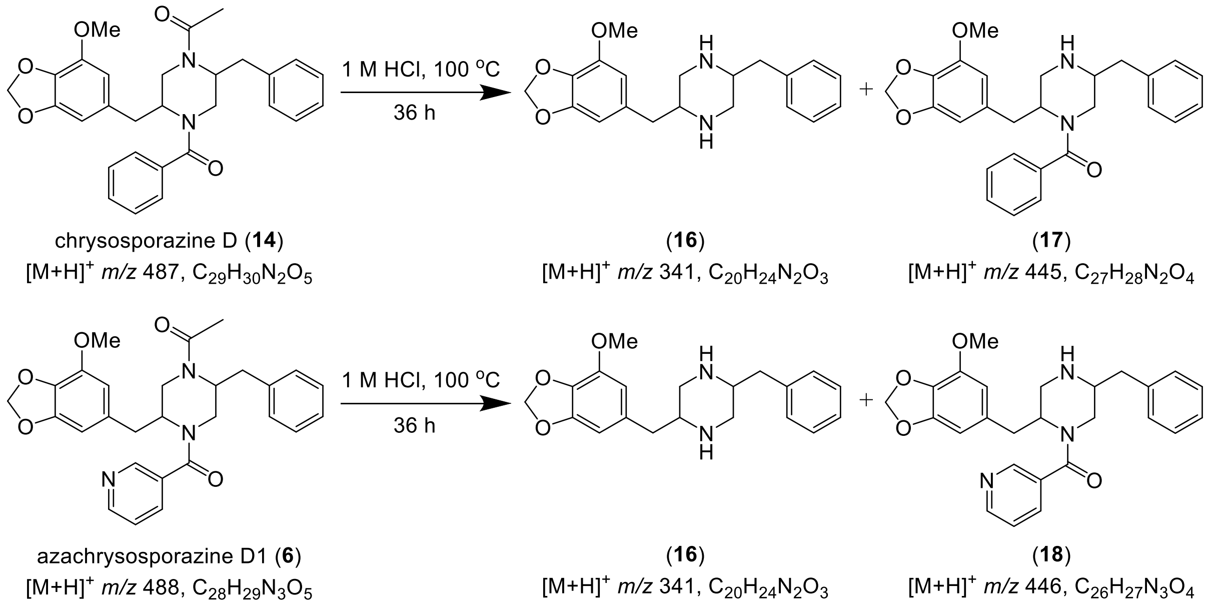

Scheme S3. Acid hydrolysis schemes of azachrysosporazine D1 (**6**) in comparison to chrysosporazine D (**14**).

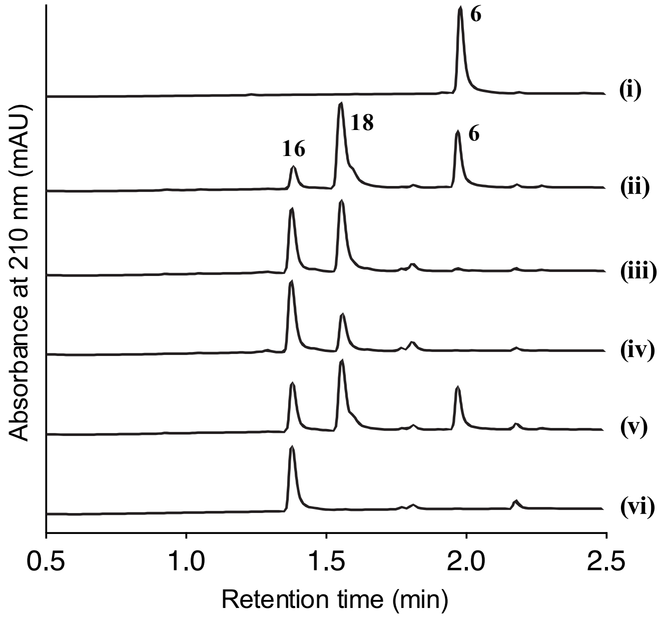

Figure S64. UPLC-DAD (210 nm) of acid hydrolysis of azachrysosporazine D1 (**6**). (i) purified **6**; acid hydrolysis of **6** at (ii) 12 hr, (iii) 24 hr and (iv) 36 hr; (v) acid hydrolysis of **6** at 12 hr co-injected with purified **16** obtained from acid hydrolysis of **14**; (vi) purified **16**.

9.2 Chrysosporazine P (9)

The final hydrolysis product (**19**) with $[M+H]^+$ at *m/z* 343 was minor and detected in UPLC-QTOF analysis by SIE.

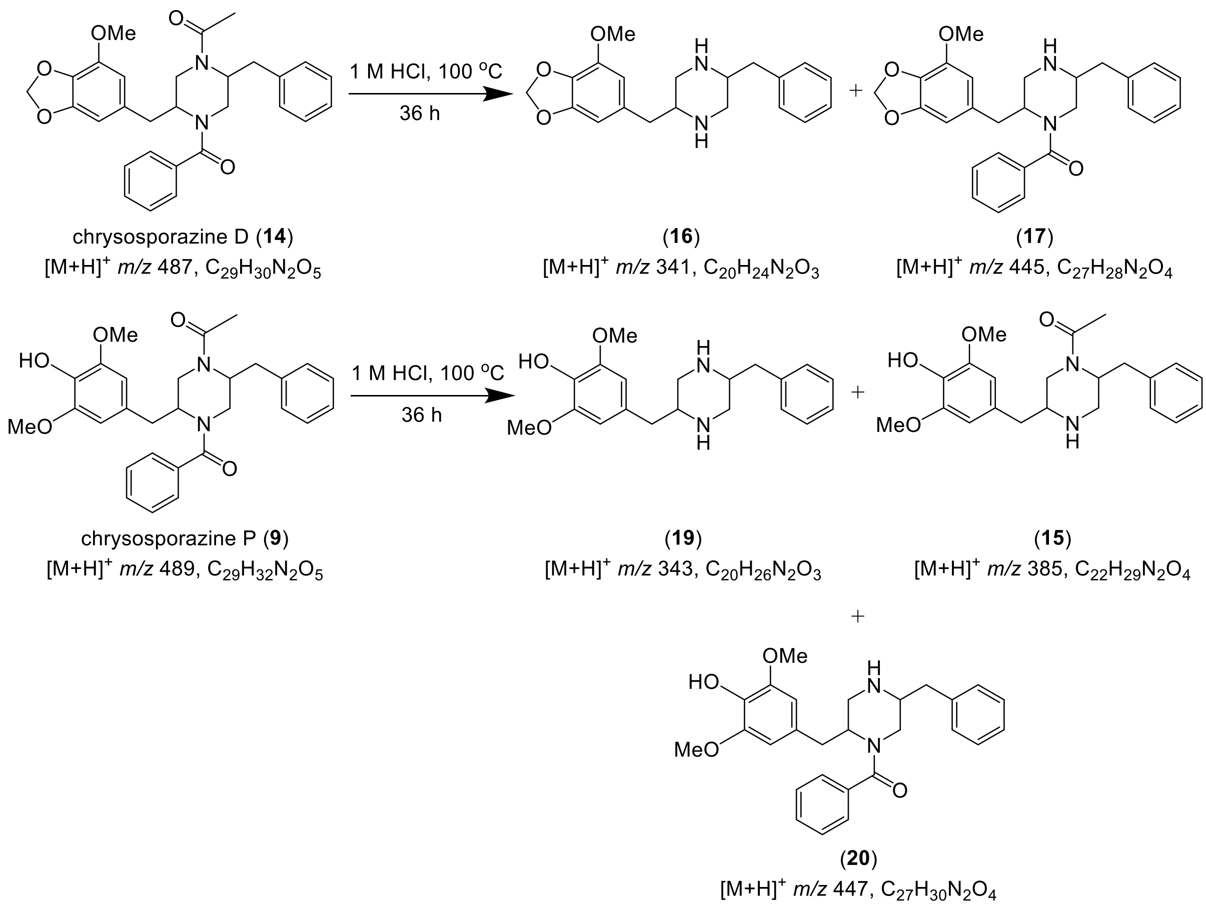

Scheme S4. Acid hydrolysis scheme of chrysosporazine P (**9**) in comparison to chrysosporazine D (**14**).

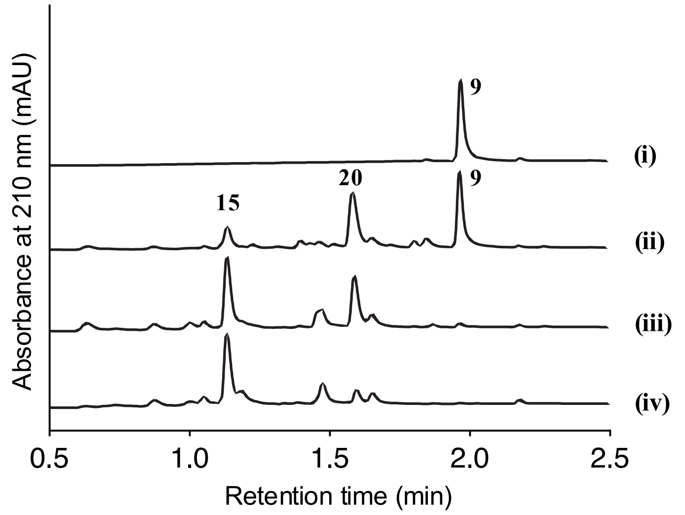

Figure S65. UPLC-DAD (210 nm) of acid hydrolysis of chrysosporazine P (**9**). (i) purified **9**; acid hydrolysis of **9** at (ii) 12 hr, (iii) 24 hr and (iv) 36 hr.

10 UPLC-QTOF-SIE study

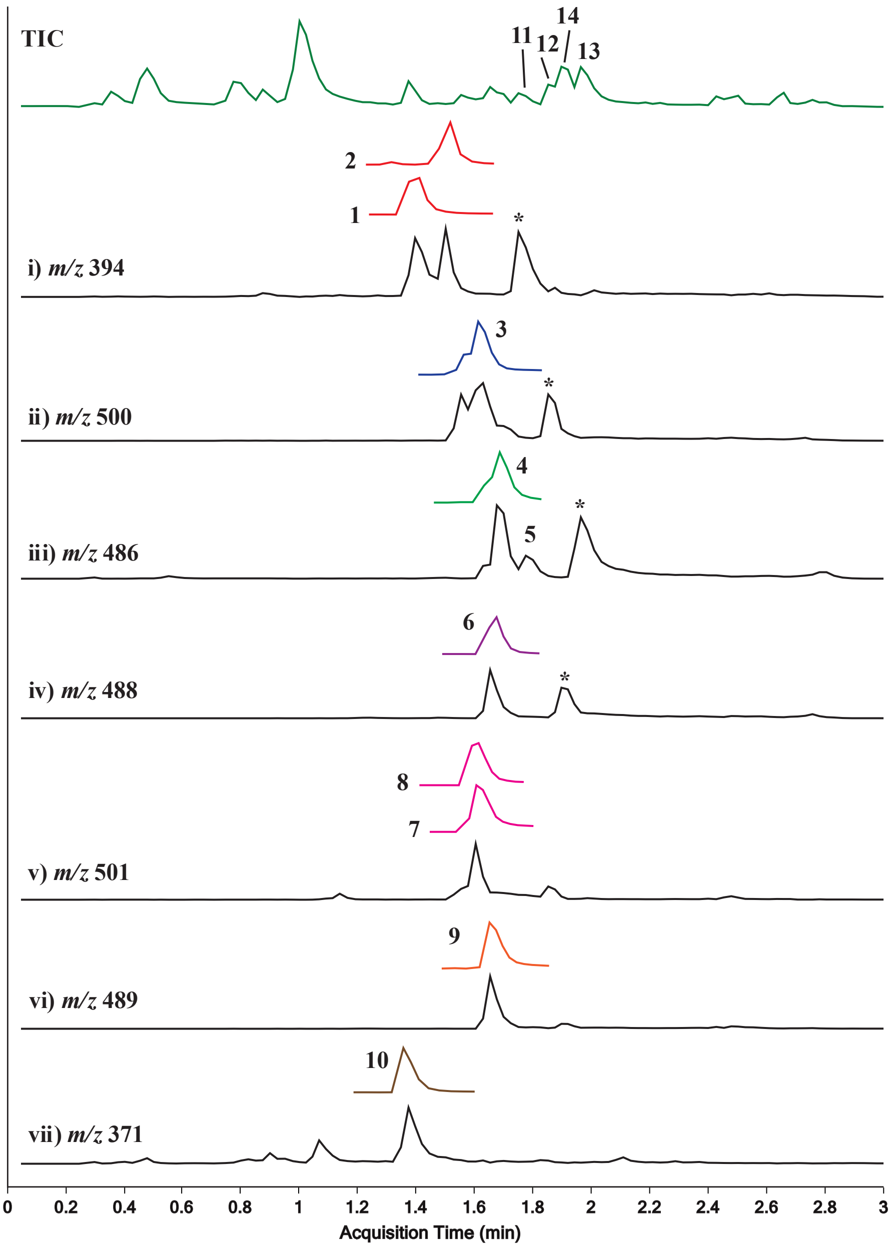

Figure S66. UPLC-QTOF-SIE analysis of an M1 agar culture of CMB-F214 (without sodium nicotinate feeding), with SIE at i) *m/z* 394, **1–2**; ii) *m/z* 500, **3**; iii) *m/z* 486, **4–5**; iv) *m/z* 488, **6**; v) *m/z* 501, **7–8**; vi) *m/z* 489, **9**; vii) *m/z* 371, **10**, along with a co-injection of authentic **1–4** and **6–10**. * refers to $[M+H+1]^+$ of corresponding natural chrysosporazines A–D (**11–14**).

11 Antimicrobial assay

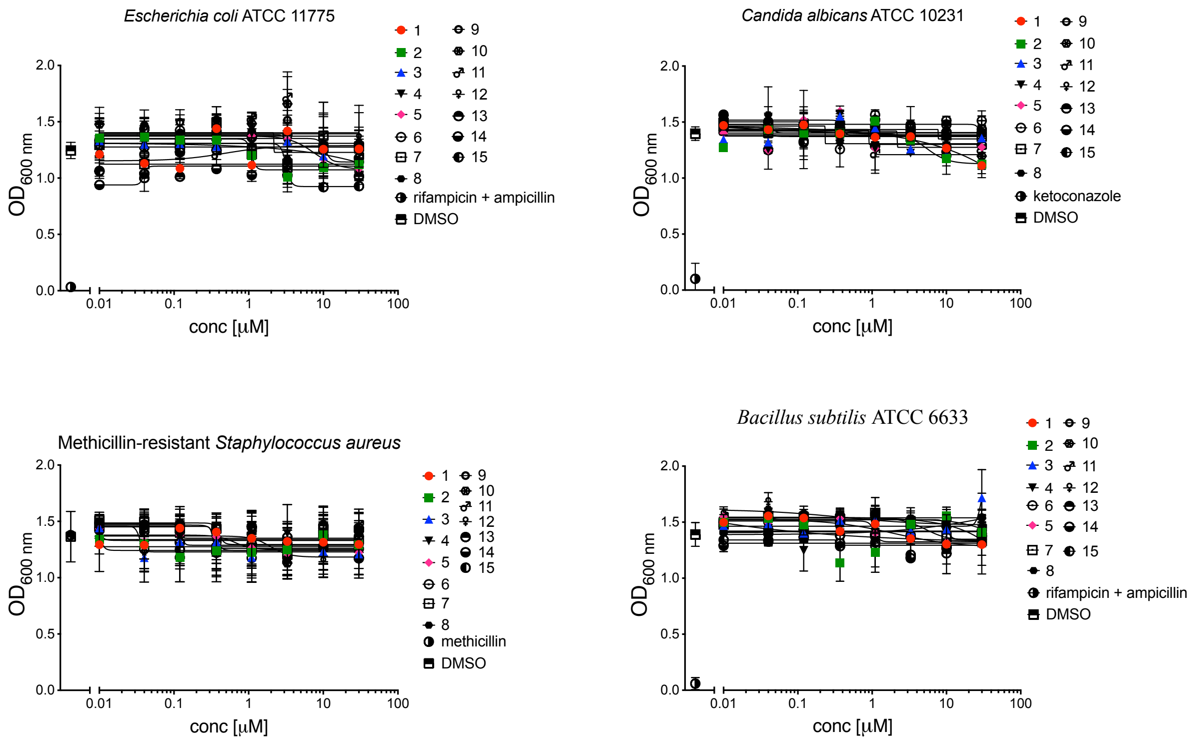

Figure S67. Growth inhibitory activity of **1-15**.

12 References

1. Elbanna, A.H.; Khalil, Z.G.; Bernhardt, P.V.; Capon, R.J. Chrysosporazines A-E: P-Glycoprotein Inhibitory Piperazines from an Australian Marine Fish Gastrointestinal Tract-Derived Fungus, *Chrysosporium* Sp. CMB-F214. *Org. Lett.* **2019**, *21*, 8097–8100.
